# Supplementary material for: Migration Testing of GPPS and HIPS Polymers: Swelling Effect Caused by Food Simulants Compared to Real Foods
Source: Molecules. 2022 Jan 26;27(3):823. doi: 10.3390/molecules27030823 (PMC8839621; doi:10.3390/molecules27030823)
Supplement: Supplementary file 1 [file molecules-27-00823-s001.zip › Suppmaterial_1_tabdata.pdf]

Article

Migration testing of GPPS and HIPS polymers: swelling effect caused by food simulants compared to real foods

Valeria Guazzotti *, Anita Gruner, Mladen Juric, Veronika Hendrich, Angela Störmer and Frank Welle

Fraunhofer Institute for Process Engineering and Packaging, IVV. Giggenhauser Straße 35, 85354 Freising. Germany

*correspondence: valeria.guazzotti@ivv.fraunhofer.de

Supplementary material -1 (Tabulated data)

Index

◎ GPPS 3
1 Migration and weight increase in isooctane at 60 °C 3
2 Migration and weight increase in isooctane at 40 °C 7
3 Migration in isooctane at 20 °C 11
4 Migration and weight increase in 95% ethanol at 60 °C 13
5 Migration and weight increase in 95% ethanol at 40 °C 17
6 Migration and weight increase in 95% ethanol at 20 °C 20
7 Migration and weight increase in 50% ethanol at 60 °C 23
8 Migration and weight increase in 50% ethanol at 40 °C 26
9 Weight increase in 50% ethanol at 20 °C 28
10 Weight increase in 20% ethanol at 60. 40 and 20 °C 29
◎ HIPS 30
11 Migration and weight increase in isooctane at 60 °C 30
12 Migration and weight increase in isooctane at 40 °C 33
13 Migration and weight increase in isooctane at 20 °C 36
14 Migration and weight increase in 95% ethanol at 60 °C 39
15 Migration and weight increase in 95% ethanol at 40 °C 43
16 Migration and weight increase in 95% ethanol at 20 °C 47
17 Migration and weight increase in 50% ethanol at 60 °C 51
18 Migration and weight increase in 50% ethanol at 40 °C 55
19 Migration and weight increase in 50% ethanol at 20 °C 57
20 Weight increase in 20% ethanol at 60. 40 and 20 °C 59
21 Weight increase in 10% ethanol at 60. 40 and 20 °C 60
22 Weight increase in 3% acetic acid at 60. 40 and 20 °C 61

❖ GPPS

1 Migration and weight increase in isooctane at 60 °C

Migration Kinetics - Results in µg/dm². Average values and standard deviation (N=3)

**Migration time [days]**		**Toluene**	**Chloro-benzene**	**Styrene**	**Phenyl-cyclo-hexane**	**Benzo-phenone**	**Methyl-stearate**
**0.02**	average	6.0	7.4	13.5	14.3	27.4	27.6
	SD	2.7	4.3	2.4	2.6	4.3	4.6
**0.04**	average	11.40	15.23	17.29	23.60	41.67	44.5
	SD	0.32	4.87	1.25	2.87	4.64	5.2
**0.08**	average	17.79	33.96	27.26	55.52	82.56	89.5
	SD	3.37	5.43	3.65	7.67	11.83	9.3
**0.17**	average	99.15	88.08	65.00	141.69	183.54	196.5
	SD	19.94	6.86	4.07	9.26	11.44	12.4
**0.25**	average	123.33	116.63	86.38	185.61	235.97	245.1
	SD	30.65	8.02	6.79	11.91	16.51	12.0
**0.33**	average	159.67	145.77	106.83	227.28	280.17	291.8
	SD	5.78	7.37	4.45	9.53	15.02	15.7
**0.42**	average	170.66	174.02	126.49	262.31	317.66	330.0
	SD	27.08	9.91	5.50	12.86	17.42	15.8
**1**	average	287.62	328.75	237.13	464.03	541.76	557.7
	SD	28.73	19.92	12.49	23.98	36.31	25.9
**2**	average	481.11	549.89	382.71	728.94	822.10	840.7
	SD	28.70	26.43	17.96	36.31	42.88	31.3
**3**	average	614.09	671.81	464.73	880.10	983.10	1005.0
	SD	66.75	32.67	20.35	36.28	62.17	40.7
**6**	average	811.46	944.60	651.99	1248.38	1380.15	1407.0
	SD	55.83	52.05	35.13	87.74	132.06	87.5
**14**	average	995.95	1105.77	764.07	1434.11	1505.88	1600.4
		73.65	50.32	36.18	94.28	98.38	116.4
**17**	average	1076.05	1109.34	767.51	1453.79	1519.64	1623.1
	SD	28.06	30.19	20.62	69.17	67.91	91.7
**21**	average	1073.85	934.59	764.64	1449.57	1533.01	1638.6
	SD	35.48	452.75	32.56	73.53	61.95	90.4
**28**	average	1062.02	1098.83	759.76	1463.71	1534.59	1649.7
	SD	29.10	34.78	22.67	59.70	59.47	72.5
**35**	average	1063.49	1097.74	759.12	1457.08	1528.58	1655.9
	SD	35.66	44.09	28.82	62.14	46.23	60.5
**42**	SD	1062.86	1105.37	765.07	1479.35	1537.12	1674.2
		43.08	52.43	35.48	67.31	64.48	77.9

Relative migration - Results in %. Average values

Migration time [days]	Toluene	Chloro-benzene	Styrene	Phenyl-cyclo-hexane	Benzo-phenone	Methyl-stearate
**0.02**	0.7	0.7	1.9	1.0	2.2	1.9
**0.04**	1.3	1.5	2.4	1.6	3.3	3.0
**0.08**	2.1	3.3	3.8	3.8	6.6	6.1
**0.17**	11.6	8.5	9.0	9.8	14.7	13.3
**0.25**	14.4	11.3	12.0	12.8	18.9	16.6
**0.33**	18.7	14.1	14.8	15.7	22.4	19.8
**0.42**	20.0	16.8	17.5	18.1	25.4	22.4
**1**	33.6	31.8	32.9	32.1	43.3	37.9
**2**	56.3	53.2	53.0	50.4	65.7	57.1
**3**	71.8	65.0	64.4	60.9	78.6	68.3
**6**	94.9	91.4	90.4	86.4	110.4	95.6
**14**	116.5	107.0	105.9	99.2	120.4	108.7
**17**	125.9	107.4	106.4	100.6	121.5	110.3
**21**	125.6	104.0	106.0	100.3	122.6	111.3
**28**	124.2	106.4	105.3	101.3	122.7	112.1
**35**	124.4	106.2	105.2	100.8	122.2	112.5
**42**	124.3	107.0	106.0	102.4	122.9	113.7

Migration Kinetics - Results in µg/dm². Average values

Migration time [days]	Oligo-1	Oligo-2	Oligo-3	Oligo-4	Oligo-5	Oligo-6	Oligo-7	Oligo-8
**0.02**	06.2	24.9	61.8	73.5	166.0	59.5	18.9	07.4
**0.04**	10.7	43.2	107.1	125.9	289.2	93.4	31.1	11.4
**0.08**	24.9	95.5	216.7	249.9	565.1	174.0	62.8	21.6
**0.17**	56.9	211.7	409.1	462.6	1056.8	314.9	120.4	36.7
**0.25**	69.7	260.4	487.1	540.5	1231.4	367.4	142.4	44.5
**0.33**	83.3	309.2	560.7	618.8	1412.0	421.2	162.9	50.6
**0.42**	94.2	349.0	622.5	682.7	1559.9	465.0	180.8	56.7
**1**	157.2	576.9	956.7	1038.1	2386.9	705.6	278.4	88.8
**2**	247.4	897.8	1406.9	1516.2	3502.9	1024.8	414.3	134.1
**3**	301.6	1093.8	1706.6	1842.6	4240.1	1250.2	474.2	158.1
**6**	388.0	1403.5	2147.5	2319.4	5326.9	1562.8	628.3	203.6
**14**	483.1	1769.1	2928.0	3220.0	7345.9	2144.9	858.0	281.2
**17**	512.8	1887.2	3227.8	3585.8	8111.5	2360.2	944.6	299.7
**21**	519.8	1893.7	3341.7	3736.9	8386.5	2431.8	974.5	308.7
**28**	503.7	1849.0	3267.8	3724.7	8272.2	2388.3	940.5	301.1
**35**	499.1	1836.7	3329.7	3819.2	8439.3	2433.0	950.7	301.4
**42**	498.4	1840.8	3342.7	3853.3	8490.3	2444.6	958.5	303.2

Relative migration - Results in %. Average values

Migration time [days]	Oligo-1	Oligo-2	Oligo-3	Oligo-4	Oligo-5	Oligo-6	Oligo-7	Oligo-8
**0.02**	1.7	2.2	2.2	3.3	3.5	4.4	3.6	4.3
**0.04**	2.9	3.8	3.8	5.6	6.1	6.9	5.8	6.5
**0.08**	6.7	8.5	7.6	11.2	11.9	12.9	11.8	12.4
**0.17**	15.4	18.8	14.4	20.7	22.2	23.4	22.6	21.1
**0.25**	18.9	23.1	17.1	24.1	25.8	27.3	26.7	25.5
**0.33**	22.6	27.4	19.7	27.6	29.6	31.3	30.6	29.0
**0.42**	25.5	30.9	21.9	30.5	32.7	34.5	33.9	32.5
**1**	42.6	51.2	33.6	46.4	50.1	52.4	52.2	50.9
**2**	67.0	79.6	49.4	67.7	73.5	76.1	77.7	76.9
**3**	81.7	97.0	59.9	82.3	89.0	92.8	89.0	90.7
**6**	105.1	124.5	75.4	103.6	111.8	116.0	117.9	116.8
**14**	130.9	156.9	102.8	143.8	154.2	159.3	161.0	161.4
**17**	139.0	167.4	113.4	160.2	170.3	175.2	177.2	172.0
**21**	140.9	168.0	117.4	166.9	176.0	180.6	182.8	177.2
**28**	136.5	164.0	114.8	166.4	173.6	177.3	176.5	172.8
**35**	135.3	162.9	116.9	170.6	177.1	180.6	178.4	173.0
**42**	135.1	163.3	117.4	172.1	178.2	181.5	179.8	174.0

Weight increase - Results in %. Average values and standard deviation (N=3)

Time contact [days]	Weight increase [%]	SD
**0.02**	0.0	0.01
**0.04**	0.0	0.01
**0.08**	0.3	0.01
**0.13**	0.6	0.01
**0.17**	0.9	0.13
**0.25**	2.7	0.12
**0.33**	3.9	0.07
**1**	11.8	0.15
**2**	17.1	0.08
**3**	21.0	0.12
**6**	30.4	0.17
**8**	35.0	0.07
**10**	45.0	0.14
**15**	49.9	0.83
**23**	57.0	0.73
**29**	63.9	0.04
**45**	71.8	0.45
**65**	80.3	0.48
**73**	87.0	0.17
**99**	88.1	3.43
**114**	92.5	3.57
**143**	95.5	0.36
**192**	96.4	2.05

2 Migration and weight increase in isooctane at 40 °C

Migration Kinetics - Results in µg/dm². Average values and standard deviation (N=3)

**Migration time [days]**		**Toluene**	**Chloro-benzene**	**Styrene**	**Phenyl-cyclo-hexane**	**Benzo-phenone**	**Methyl-stearate**
**0.25**	average		4.3	3.7	1.5	3.2	6.7
	SD		5.8	1.3	0.3	1.6	1.9
**0.5**	average		6.6	4.2	2.1	3.3	6.3
	SD		7.9	2.7	0.4	0.5	0.3
**1**	average		16.2	8.3	4.4	6.7	8.1
	SD		2.7	1.3	0.8	1.2	0.7
**2**	average		25.1	11.9	8.1	15.9	13.2
	SD		4.5	2.0	0.7	9.6	1.8
**6**	average		40.3	17.5	13.8	20.3	21.3
	SD		5.5	2.4	2.0	1.6	2.3
**14**	average		60.2	24.4	20.4	30.2	32.3
	SD		7.5	3.2	2.4	2.7	3.1
**17**	average		66.9	29.2	22.4	35.9	38.5
	SD		7.3	3.0	2.6	5.0	3.4
**21**	average		77.2	32.3	25.5	40.2	42.9
	SD		7.4	3.2	2.1	4.2	3.2
**28**	average		89.8	37.0	30.4	45.2	48.0
	SD		7.5	4.2	3.3	4.7	2.7
**35**	average		100.0	42.4	34.0	47.7	52.6
	SD		7.8	3.5	2.1	2.4	2.1
**42**	average		112.7	47.6	38.3	53.7	61.2
	SD		8.0	3.6	2.6	3.3	4.3
**49**	average		122.6	52.7	41.9	59.5	67.4
	SD		6.3	3.3	2.4	4.2	4.2
**56**	average		131.9	44.9	45.9	60.9	61.6
	SD		8.1	24.9	3.2	3.8	4.4
**63**	average		138.7	57.9	48.7	61.6	48.4
	SD		11.9	5.5	4.6	5.0	7.6
**70**	average		143.0	60.2	51.2	67.6	62.3
	SD		5.8	2.8	2.8	3.1	3.3
**80**	average		156.7	64.4	56.6	79.3	76.6
	SD		9.4	4.0	3.4	4.1	3.8
**91**	average		169.5	70.0	62.2	87.2	84.0
	SD		11.2	4.9	4.0	6.7	6.2
**101**	average		183.3	76.5	67.7	88.2	67.8
	SD		14.4	6.8	5.8	5.7	18.9
**108**	average		197.4	82.8	73.6	105.0	110.2
	SD		7.7	3.7	3.2	5.4	12.6
**122**	average		124.7	49.5	43.9	64.5	65.9
	SD		5.5	2.3	1.8	2.2	2.5

**139**	average	153.8	54.3	49.1	71.2	73.9
	SD	36.5	2.4	2.4	3.7	3.1

Note: Due to analytical interferences. migration of toluene from GPPS isooctane at 40 °C can not be measured

Relative migration - Results in %. Average values

**Migration time [days]**	**Toluene**	**Chloro-benzene**	**Styrene**	**Phenyl-cyclo-hexane**	**Benzo-phenone**	**Methyl-stearate**
**0.25**		0.4	0.5	0.1	0.3	0.5
**0.5**		0.6	0.6	0.1	0.3	0.4
**1**		1.6	1.1	0.3	0.5	0.6
**2**		2.4	1.6	0.6	1.3	0.9
**6**		3.9	2.4	1.0	1.6	1.4
**14**		5.8	3.4	1.4	2.4	2.2
**17**		6.5	4.0	1.6	2.9	2.6
**21**		7.5	4.5	1.8	3.2	2.9
**28**		8.7	5.1	2.1	3.6	3.3
**35**		9.7	5.9	2.4	3.8	3.6
**42**		10.9	6.6	2.6	4.3	4.2
**49**		11.9	7.3	2.9	4.8	4.6
**56**		12.8	6.2	3.2	4.9	4.2
**63**		13.4	8.0	3.4	4.9	3.3
**70**		13.8	8.3	3.5	5.4	4.2
**80**		15.2	8.9	3.9	6.3	5.2
**91**		16.4	9.7	4.3	7.0	5.7
**101**		17.7	10.6	4.7	7.1	4.6
**108**		19.1	11.5	5.1	8.4	7.5
**122**		12.1	6.9	3.0	5.2	4.5
**139**		14.9	7.5	3.4	5.7	5.0

Migration Kinetics - Results in µg/dm². Average values

**Migration time [days]**	**Oligo-1**	**Oligo-2**	**Oligo-3**	**Oligo-4**	**Oligo-5**	**Oligo-6**	**Oligo-7**	**Oligo-8**
**0.25**	n.d.	6.6	7.5	9.9	26.1	1.8	3.4	n.d.
**0.5**	n.d.	7.3	11.9	10.9	25.0	10.4	3.7	1.6
**1**	n.d.	10.5	18.2	20.1	45.2	24.6	5.1	1.6
**2**	3.6	15.4	28.8	26.8	86.7	33.5	8.6	3.3
**6**	6.3	24.9	54.7	67.8	149.0	55.5	16.1	4.5
**14**	9.2	35.4	70.1	105.4	228.7	77.9	24.6	8.1
**17**	12.7	40.2	131.1	126.7	276.1	95.5	32.5	9.9
**21**	13.4	44.5	130.9	140.8	306.3	104.1	34.0	11.2
**28**	13.5	49.0	131.9	121.5	329.6	106.3	35.3	9.3
**35**	14.6	55.9	150.0	177.6	384.4	125.7	34.5	13.1

**42**	15.9	60.0	163.8	193.6	418.7	130.6	44.4	14.3
**49**	18.7	67.5	189.2	181.0	476.4	146.8	51.1	15.9
**56**	16.6	66.1	163.9	205.5	444.9	134.3	46.7	14.3
**63**	18.7	75.8	182.1	228.2	488.9	145.2	52.3	15.4
**70**	20.3	79.6	194.3	243.1	522.6	155.7	55.9	16.7
**80**	23.5	86.3	213.7	264.1	571.6	171.3	42.5	18.8
**91**	24.0	92.5	230.3	283.8	613.3	181.3	55.0	20.3
**101**	25.9	101.0	241.0	301.9	649.2	188.9	64.0	21.3
**108**	26.9	105.8	265.3	624.5	667.0	201.5	70.5	23.9
**122**	31.3	115.3	342.9	435.8	947.7	286.8	111.2	35.5
**139**	33.1	123.7	365.1	461.0	1011.7	302.1	114.4	37.6

Relative migration - Results in %. Average values

**Migration time [days]**	**Oligo-1**	**Oligo-2**	**Oligo-3**	**Oligo-4**	**Oligo-5**	**Oligo-6**	**Oligo-7**	**Oligo-8**
**0.25**	0.0	0.6	0.3	0.4	0.5	0.1	0.6	n.d.
**0.5**	0.0	0.6	0.4	0.5	0.5	0.8	0.7	0.9
**1**	0.0	0.9	0.6	0.9	0.9	1.8	1.0	0.9
**2**	1.0	1.4	1.0	1.2	1.8	2.5	1.6	1.9
**6**	1.7	2.2	1.9	3.0	3.1	4.1	3.0	2.6
**14**	2.5	3.1	2.5	4.7	4.8	5.8	4.6	4.7
**17**	3.5	3.6	4.6	5.7	5.8	7.1	6.1	5.7
**21**	3.6	3.9	4.6	6.3	6.4	7.7	6.4	6.4
**28**	3.7	4.3	4.6	5.4	6.9	7.9	6.6	5.3
**35**	4.0	5.0	5.3	7.9	8.1	9.3	6.5	7.5
**42**	4.3	5.3	5.8	8.6	8.8	9.7	8.3	8.2
**49**	5.1	6.0	6.6	8.1	10.0	10.9	9.6	9.1
**56**	4.5	5.9	5.8	9.2	9.3	10.0	8.8	8.2
**63**	5.1	6.7	6.4	10.2	10.3	10.8	9.8	8.8
**70**	5.5	7.1	6.8	10.9	11.0	11.6	10.5	9.6
**80**	6.4	7.7	7.5	11.8	12.0	12.7	8.0	10.8
**91**	6.5	8.2	8.1	12.7	12.9	13.5	10.3	11.6
**101**	7.0	9.0	8.5	13.5	13.6	14.0	12.0	12.2
**108**	7.3	9.4	9.3	27.9	14.0	15.0	13.2	13.7
**122**	8.5	10.2	12.0	19.5	19.9	21.3	20.9	20.4
**139**	9.0	11.0	12.8	20.6	21.2	22.4	21.5	21.6

Weight increase- Results in %. Average values and standard deviation (N=3)

**Time contact [days]**	**Weight increase [%]**	**SD**
**0.17**	0.1	0.01
**0.35**	0.1	0.01
**1**	0.1	0.01
**2**	0.1	0.02
**3**	0.1	0.02
**6**	0.1	0.02
**8**	0.2	0.01
**13**	0.3	0.01
**17**	0.3	0.01
**23**	0.4	0.01
**29**	0.6	0.03
**45**	0.9	0.03
**65**	1.1	0.01
**99**	1.7	0.03
**114**	1.9	0.05

3 Migration in isooctane at 20 °C

Migration Kinetics - Results in µg/dm². Average values and standard deviation (N=3)

Migration time [days]		Toluene	Chloro-benzene	Styrene	Phenyl-cyclo-hexane	Benzo-phenone	Methyl-stearate
**1**	average			3.88	0.78	1.06	1.30
	SD			1.96	0.00	0.00	0.50
**3**	average		4.96	4.35	1.87	2.34	2.93
	SD		3.28	2.18	0.14	1.08	0.62
**6**	average		6.45	4.56	2.16	3.14	3.82
	SD		3.01	3.38	0.00	0.89	0.37
**14**	average		15.24	8.30	2.97	4.66	5.12
	SD		1.19	0.84	0.28	0.40	0.51
**21**	average		10.89	10.68	3.78	7.78	6.58
	SD		1.28	0.75	0.57	4.49	0.41
**28**	average		16.53	10.88	4.07	6.18	6.02
	SD		5.43	1.78	0.19	0.50	0.40
**35**	average		19.99	11.15	4.53	7.22	6.75
	SD		5.60	2.99	0.14	0.56	0.84
**42**	average		21.76	13.06	4.59	7.86	6.75
	SD		7.38	1.03	0.62	1.86	0.48
**49**	average		29.87	14.28	4.64	7.94	7.32
	SD		2.92	1.48	0.34	0.66	0.53
**56**	average		32.44	12.99	5.05	6.26	5.93
	SD		3.05	1.25	0.18	0.36	0.78
**63**	average		30.96	10.88	4.93	6.50	6.10
	SD		2.90	5.25	0.66	0.58	0.67
**70**	average		32.34	11.36	5.28	6.98	6.10
	SD		2.96	5.60	0.58	0.78	0.41
**80**	average		34.91	13.33	5.45	7.86	6.18
	SD		3.47	1.50	0.75	1.53	0.91
**91**	average		37.97	14.55	5.57	7.54	6.34
	SD		3.41	1.17	0.51	0.59	0.31
**101**	average		41.43	14.76	5.91	8.18	3.98
	SD		4.43	1.85	0.55	1.59	0.37
**108**	average		43.80	16.52	1.87	1.06	
	SD		4.13	2.26	2.18	0.00	
**122**	average		27.00	8.23	3.95	7.62	4.80
	SD		2.79	1.05	0.34	1.08	2.59
**139**	average		29.18	8.57	4.35	7.46	5.93
	SD		3.51	1.32	0.42	0.39	0.20

Note: Due to analytical interferences. migration of toluene from GPPS isooctane at 20 °C can not be measured

Relative migration - Results in %. Average values

Migration time [days]	Toluene	Chloro-benzene	Styrene	Phenyl-cyclo-hexane	Benzo-phenone	Methyl-stearate
**1**		0.0	0.5	0.1	0.1	0.0
**3**		0.5	0.6	0.1	0.2	0.2
**6**		0.6	0.6	0.1	0.3	0.3
**14**		1.5	1.1	0.2	0.4	0.3
**21**		1.1	1.5	0.3	0.6	0.4
**28**		1.6	1.5	0.3	0.5	0.4
**35**		1.9	1.5	0.3	0.6	0.5
**42**		2.1	1.8	0.3	0.6	0.5
**49**		2.9	2.0	0.3	0.6	0.5
**56**		3.1	1.8	0.3	0.5	0.4
**63**		3.0	1.5	0.3	0.5	0.4
**70**		3.1	1.6	0.4	0.6	0.4
**80**		3.4	1.8	0.4	0.6	0.4
**91**		3.7	2.0	0.4	0.6	0.4
**101**		4.0	2.0	0.4	0.7	0.3
**108**		4.2	2.3	0.1	0.1	0.0
**122**		2.6	1.1	0.3	0.6	0.3
**139**		2.8	1.2	0.3	0.6	0.4

Migration of oligomers from GPPS into isooctane at 20 °C was not detectable.

The weight increase of GPPS in isooctane at 20 °C remains < 0.01% until 73 days.

4 Migration and weight increase in 95% ethanol at 60 °C

Migration Kinetics - Results in µg/dm². Average values and standard deviation (N=3)

Migration time [days]		Toluene	Chloro-benzene	Styrene	Phenyl-cyclo-hexane	Benzo-phenone	Methyl-stearate
**0.04**	average	20.3		28.6	152.2	5.9	23.8
	SD	0.0		1.1	2.7	0.5	1.2
**0.08**	average	24.7		41.9	154.7	9.4	31.2
	SD	0.6		1.4	5.7	0.6	3.1
**0.17**	average	31.5	47.3	57.5	153.3	14.6	41.3
	SD	1.0	1.9	2.4	4.0	1.5	3.3
**0.25**	average	36.1	62.3	65.4	153.3	17.3	44.7
	SD	3.5	1.9	1.9	3.4	1.0	2.2
**0.33**	average	59.6	76.6	73.1	152.5	18.7	49.5
	SD	5.6	4.0	2.5	3.3	3.1	4.1
**0.42**	average	67.6	90.1	81.1	151.7	31.6	53.4
	SD	6.0	3.6	2.1	1.3	22.5	3.1
**1**	average	141.6	197.8	138.8	155.2	43.5	86.7
	SD	8.1	5.1	3.9	4.6	1.1	3.2
**2**	average	270.5	346.2	220.4	154.1	74.4	134.0
	SD	26.9	9.7	5.6	2.4	2.0	4.4
**3**	average	388.2	383.7	283.9	156.6	100.3	171.4
	SD	35.9	188.1	9.0	5.1	2.1	6.6
**7**	average	666.0	777.0	461.5	154.7	169.6	272.8
	SD	18.2	21.5	12.2	4.6	1.3	7.3
**10**	average	720.3	914.2	554.9	151.1	210.3	329.6
	SD	45.7	40.3	20.7	0.0	6.7	12.1
**14**	average	861.1	1011.7	631.8		260.1	393.9
	SD	87.7	27.6	21.5		8.5	16.1
**21**	average	942.7	1091.1	708.1	151.1	326.9	487.5
	SD	30.1	20.6	15.5	0.0	6.8	22.8
**28**	average	1016.7	957.7	749.4	151.1	387.9	581.4
	SD	140.7	396.0	16.0	0.0	9.2	19.1
**35**	average	1014.5	1132.0	772.3	151.1	440.0	665.2
	SD	117.9	33.0	26.6	0.0	17.2	32.0
**42**	average	921.9	1080.6	863.9	151.1	495.0	664.9
	SD	107.3	57.6	23.6	0.0	19.2	25.6
**60**	average	880.9	1051.9	728.8	151.1	505.2	668.4
	SD	151.5	56.7	40.2	0.0	19.4	41.3
**73**	average	845.4	1102.0	768.3	151.1	579.0	785.7
	SD	47.2	83.7	62.8	0.0	45.6	65.1
**84**	average	805.3	1011.9	704.0	151.1	592.8	856.1
	SD	101.5	55.2	27.5	0.0	16.6	41.9
**94**	average	816.3	802.7	680.5	151.1	611.7	888.7
	SD	61.0	400.2	42.2	0.0	31.6	49.8

**105**	average	841.0	1003.1	713.4	151.1	673.0	958.8
	SD	76.7	61.3	49.5	0.0	51.2	64.0

Relative migration - Results in %. Average values

Migration time [days]	Toluene	Chloro-benzene	Styrene	Phenyl-cyclo-hexane	Benzo-phenone	Methyl-stearate
**0.04**	2.4	0.0	4.0	0.3	1.9	4.4
**0.08**	2.4	0.0	4.0	0.4	1.9	4.4
**0.17**	2.9	0.9	5.8	0.7	2.5	4.8
**0.25**	3.7	4.6	8.0	1.0	3.3	5.5
**0.33**	4.2	6.0	9.1	1.2	3.6	5.5
**0.42**	7.0	7.4	10.1	1.3	4.0	5.8
**1**	7.9	8.7	11.2	2.2	4.3	5.9
**2**	16.6	19.1	19.2	3.0	6.9	7.4
**3**	31.6	33.5	30.5	5.1	10.7	9.3
**7**	45.4	37.1	39.3	6.9	13.7	11.2
**10**	77.9	75.2	64.0	11.7	21.8	14.9
**14**	84.3	88.5	76.9	14.5	26.4	20.0
**21**	100.7	97.9	87.6	18.0	31.5	19.0
**28**	109.1	104.5	96.5	21.8	37.6	23.0
**35**	118.9	92.7	103.8	26.8	46.5	30.3
**42**	118.7	109.6	107.0	30.4	53.2	41.0
**60**	107.8	104.6	119.7	34.2	53.2	31.3
**73**	103.0	101.8	101.0	35.0	53.4	36.8
**84**	98.9	106.7	106.5	40.1	62.8	42.2
**94**	94.2	97.9	97.6	41.0	68.5	44.2
**105**	95.5	77.7	94.3	42.3	71.1	49.9

Migration Kinetics - Results in μg/dm². Average values

Migration time [days]	Oligo-1	Oligo-2	Oligo-3	Oligo-4	Oligo-5	Oligo-6	Oligo-7	Oligo-8
**0.04**	n.d.	5.4	3.1	3.4	10.7	n.d.	n.d.	n.d.
**0.08**	n.d.	8.2	4.0	4.9	15.9	4.0	n.d.	n.d.
**0.17**	2.6	11.8	5.8	7.0	20.0	5.5	n.d.	n.d.
**0.25**	5.3	13.3	6.5	7.6	19.9	5.8	n.d.	n.d.
**0.33**	4.9	14.5	7.2	8.0	21.2	6.3	n.d.	n.d.
**0.42**	5.3	15.6	7.6	7.6	21.9	6.5	2.5	n.d.
**1**	10.5	25.5	11.5	n.d.	33.1	10.0	3.7	n.d.
**2**	18.0	39.6	17.2	14.9	47.8	14.3	6.1	n.d.
**3**	24.0	51.4	21.4	21.5	58.0	17.8	7.9	2.7
**7**	35.6	80.1	31.4	29.9	83.4	25.0	11.5	4.7
**10**	43.4	96.8	37.1	35.2	96.2	29.1	13.2	5.5
**14**	51.7	117.2	42.7	40.7	112.2	33.6	15.0	6.9

**21**	67.2	152.3	57.7	53.1	150.0	43.7	20.6	8.5
**28**	81.1	180.9	66.0	62.0	167.0	52.6	25.8	9.4
**35**	91.5	201.8	76.6	69.0	191.3	62.6	24.7	10.4
**42**	107.3	235.1	106.7	78.2	217.4	75.9	27.7	17.0
**60**	122.8	267.4	96.8	84.3	240.6	75.6	36.8	16.0
**73**	141.8	313.2	110.6	95.6	280.1	84.9	39.7	27.6
**84**	153.2	335.0	119.2	103.4	291.8	92.6	49.7	19.4
**94**	158.6	347.3	133.8	116.2	328.9	104.6	44.3	20.9
**105**	165.4	363.8	138.7	120.3	336.3	103.2	49.5	21.4

Relative migration - Results in %. Average values

**Migration time [days]**	**Oligo-1**	**Oligo-2**	**Oligo-3**	**Oligo-4**	**Oligo-5**	**Oligo-6**	**Oligo-7**	**Oligo-8**
**0.04**	n.d.	0.4	n.d.	0.1	0.2	0.2	n.d.	n.d.
**0.08**	n.d.	0.5	0.1	0.2	0.2	n.d.	n.d.	n.d.
**0.17**	n.d.	0.7	0.1	0.2	0.3	0.3	n.d.	n.d.
**0.25**	0.7	1.0	0.2	0.3	0.4	0.4	n.d.	n.d.
**0.33**	1.4	1.2	0.2	0.3	0.4	0.4	n.d.	n.d.
**0.42**	1.3	1.3	0.3	0.4	0.4	0.5	n.d.	n.d.
**1**	1.4	1.4	0.3	0.3	0.5	0.5	0.5	n.d.
**2**	2.9	2.3	0.4	n.d.	0.7	0.7	0.7	n.d.
**3**	4.9	3.5	0.6	0.7	1.0	1.1	1.2	n.d.
**7**	6.5	4.6	0.8	1.0	1.2	1.3	1.5	1.5
**10**	9.6	7.1	1.1	1.3	1.7	1.9	2.2	2.7
**14**	11.8	8.6	1.3	1.6	2.0	2.2	2.5	3.1
**21**	14.0	10.4	1.5	1.8	2.4	2.5	2.8	4.0
**28**	18.2	13.5	2.0	2.4	3.1	3.2	3.9	4.9
**35**	22.0	16.0	2.3	2.8	3.5	3.9	4.8	5.4
**42**	24.8	17.9	2.7	3.1	4.0	4.6	4.6	5.9
**60**	29.1	20.9	3.7	3.5	4.6	5.6	5.2	9.7
**73**	33.3	23.7	3.4	3.8	5.0	5.6	6.9	9.2
**84**	38.4	27.8	3.9	4.3	5.9	6.3	7.4	15.8
**94**	41.5	29.7	4.2	4.6	6.1	6.9	9.3	11.1
**105**	43.0	30.8	4.7	5.2	6.9	7.8	8.3	12.0

Weight increase - Results in %. Average values and standard deviation (N=3)

Time contact [days]	Weight increase [%]	SD
**0.02**	0.2	0.07
**0.04**	0.3	0.05
**0.08**	0.4	0.05
**0.13**	0.6	0.05
**0.17**	0.7	0.09
**0.25**	1.0	0.06
**0.38**	1.1	0.05
**1**	3.0	0.10
**2**	3.4	0.09
**5**	3.8	0.04
**8**	3.9	0.08
**12**	4.1	0.05
**15**	4.1	0.04
**19**	4.4	0.20
**22**	4.4	0.18
**26**	4.4	0.08
**44**	5.5	0.13
**48**	5.4	0.11
**57**	6.1	0.04
**72**	6.9	0.91
**100**	8.1	0.95
**127**	11.0	0.10
**154**	11.9	0.20
**169**	13.3	0.25
**198**	15.9	0.05

5 Migration and weight increase in 95% ethanol at 40 °C

Migration Kinetics - Results in µg/dm². Average values and standard deviation (N=3)

Migration time [days]		Toluene	Chloro-benzene	Styrene	Phenyl-cyclo-hexane	Benzo-phenone	Methyl-stearate
**0.21**	average	28.3	0.7	27.7	3.8	20.0	58.8
	SD	1.2	3.5	0.0	0.6	1.0	1.0
**0.42**	average	29.9	10.0	27.7	4.9	24.0	58.8
	SD	1.1	12.4	0.0	0.4	2.4	1.8
**1**	average	24.7	28.1	39.9	7.5	27.3	60.3
	SD	1.0	1.9	0.8	0.7	3.5	1.5
**2**	average	37.9	51.6	51.2	10.2	31.7	61.5
	SD	3.1	1.7	1.7	1.1	1.7	1.3
**3**	average	44.1	72.3	62.8	12.3	35.6	67.2
	SD	9.2	2.5	1.7	0.8	2.1	2.4
**7**	average	86.4	137.1	94.6	18.7	45.9	71.3
	SD	5.9	3.6	1.5	0.8	1.3	2.3
**10**	average	109.1	176.8	115.0	22.6	53.1	74.3
	SD	1.6	3.7	0.4	0.8	2.6	1.1
**14**	average	129.2	266.3	132.5	26.0	60.6	81.9
	SD	5.6	102.7	6.4	5.4	2.3	1.0
**21**	average	162.6	280.6	161.1	30.9	69.3	92.5
	SD	6.3	9.3	7.5	8.0	3.3	1.5
**28**	average	248.9	349.3	197.8	39.3	80.0	103.7
	SD	4.5	12.2	18.2	1.7	3.5	2.5
**35**	average	274.7	396.1	211.1	42.0	85.9	100.8
	SD	17.6	12.5	33.5	2.8	2.1	2.3
**42**	average	251.5	377.1	265.0	47.8	87.1	93.1
	SD	4.3	22.6	2.6	1.7	7.7	5.0
**62**	average	349.7	484.6	266.9	48.5	96.8	116.2
	SD	64.5	20.1	10.2	2.4	3.5	1.8
**73**	average	387.2	557.6	303.0	53.7	106.2	122.1
	SD	31.0	38.8	17.1	2.9	2.4	2.1
**84**	average	443.0	573.5	310.8	54.4	110.8	123.1
	SD	42.2	47.6	23.3	2.2	2.8	1.7
**94**	average	505.3	599.6	324.1	58.3	118.6	122.1
	SD	29.2	29.2	14.5	2.9	4.0	4.3
**105**	average	451.8	612.7	333.3	60.4	124.5	129.3
	SD	18.0	28.5	13.8	2.1	3.5	2.7

Relative migration - Results in %. Average values

Migration time [days]	Toluene	Chloro-benzene	Styrene	Phenyl-cyclo-hexane	Benzo-phenone	Methyl-stearate
**0.21**	3.31	0.07	3.84	0.26	1.60	4.00
**0.42**	3.50	0.97	3.84	0.34	1.92	4.00
**1**	2.89	2.72	5.52	0.52	2.18	4.10
**2**	4.43	4.99	7.10	0.70	2.53	4.18
**3**	5.16	7.00	8.70	0.85	2.85	4.56
**7**	10.11	13.27	13.11	1.30	3.67	4.84
**10**	12.76	17.11	15.94	1.56	4.25	5.05
**14**	15.11	25.77	18.37	1.80	4.85	5.56
**21**	19.03	27.15	22.32	2.14	5.54	6.28
**28**	29.11	33.80	27.41	2.72	6.40	7.04
**35**	32.14	38.34	29.25	2.91	6.87	6.85
**42**	29.42	36.50	36.72	3.31	6.97	6.33
**62**	40.91	46.90	36.99	3.36	7.74	7.90
**73**	45.30	53.96	41.99	3.72	8.50	8.29
**84**	51.82	55.51	43.08	3.77	8.86	8.36
**94**	59.11	58.04	44.92	4.03	9.49	8.29
**105**	52.85	59.30	46.19	4.18	9.96	8.78

Migration Kinetics - Results in µg/dm². Average values

Migration time [days]	Oligo-1	Oligo-2	Oligo-3	Oligo-4	Oligo-5	Oligo-6	Oligo-7	Oligo-8
**0.21**		4.99		2.68	7.68	1.51		
**0.42**		5.46		3.32	8.46	2.25		
**1**								
**2**		8.01		4.06	11.10	2.86		
**3**		9.14		4.76	11.44			
**7**								
**10**								
**14**	4.90	15.55	3.64	6.00	16.39	4.37		
**21**	4.90	18.57	4.57	7.11	25.37	5.72	2.30	
**28**		22.11	4.64	7.18	21.19	6.28	1.86	
**35**	7.91	21.65	4.58	7.32	19.17	6.74	1.26	
**42**		15.07	18.70	7.64	19.71	7.30		
**62**	14.16	27.54	7.78	8.84	30.77	7.95	4.11	1.38
**73**	15.70	29.61	6.95	8.76	26.24	4.24	3.39	1.76
**84**	16.40	31.35	7.73	9.71	28.58	8.16	4.20	1.87
**94**	17.22	32.71	7.80	9.78	29.22	9.98	4.11	2.40
**105**	16.50	32.44	8.17	9.01	27.95	8.08	4.78	2.73

Relative migration - Results in %. Average values

Migration time [days]	Oligo-1	Oligo-2	Oligo-3	Oligo-4	Oligo-5	Oligo-6	Oligo-7	Oligo-8
**0.21**		0.44		0.12	0.16	0.11		
**0.42**		0.48		0.15	0.18	0.17		
**1**								
**2**		0.71		0.18	0.23	0.21		
**3**		0.81		0.21	0.24			
**7**								
**10**								
**14**		1.38	0.13	0.27	0.34	0.32		
**21**	1.33	1.65	0.16	0.32	0.53	0.42	0.43	
**28**		1.96	0.16	0.32	0.44	0.47	0.35	
**35**	2.14	1.92	0.16	0.33	0.40	0.50	0.24	
**42**		1.34	0.66	0.34	0.41	0.54		
**62**	3.84	2.44	0.27	0.40	0.65	0.59	0.77	0.79
**73**	4.25	2.63	0.24	0.39	0.55	0.31	0.64	1.01
**84**	4.44	2.78	0.27	0.43	0.60	0.61	0.79	1.07
**94**	4.67	2.90	0.27	0.44	0.61	0.74	0.77	1.38
**105**	4.47	2.88	0.29	0.40	0.59	0.60	0.90	1.56

Weight increase - Results in %. Average values and standard deviation (N=3)

Time contact [days]	Weight increase [%]	SD
**0.16**	0.6	0.09
**0.33**	0.7	0.04
**1**	1.3	0.05
**2**	1.6	0.05
**5**	2.2	0.33
**8**	2.6	0.34
**12**	2.8	0.05
**15**	2.9	0.22
**19**	3.0	0.21
**22**	3.1	0.08
**26**	3.1	0.34
**44**	3.1	0.33
**48**	3.1	0.06
**57**	3.2	0.24
**72**	3.0	0.22
**100**	2.9	0.07
**127**	2.9	0.18
**154**	2.9	0.17
**169**	2.9	0.05

6 Migration and weight increase in 95% ethanol at 20 °C

Migration Kinetics - Results in µg/dm². Average values and standard deviation (N=3)

Migration time [days]		Toluene	Chloro-benzene	Styrene	Phenyl-cyclo-hexane	Benzo-phenone	Methyl-stearate
**1**	average	37.5	8.9		2.2	12.7	37.5
	SD	4.5	0.0		0.7	1.2	1.0
**2**	average	38.1	8.9		3.2	13.8	39.3
	SD	1.5	0.0		0.4	1.9	2.7
**3**	average	37.5	8.9		3.7	16.0	37.9
	SD	1.2	0.0		0.7	3.0	1.4
**7**	average	38.1	8.9		4.2	15.2	39.1
	SD	0.5	0.0		0.4	3.3	1.6
**10**	average	35.7	24.1		4.0	15.7	40.7
	SD	3.6	7.5		1.3	2.0	2.3
**14**	average	35.3	47.7		7.8	16.1	41.1
	SD	1.3	33.3		5.2	1.3	1.8
**21**	average	31.1	40.5	3.2	5.6	19.3	45.2
	SD	0.5	4.7	8.3	0.4	1.5	2.0
**28**	average	36.9	52.3	13.9	6.1	13.3	40.9
	SD	0.9	1.9	1.8	0.4	2.7	4.5
**35**	average	40.4	59.9	19.9	8.7	13.2	39.7
	SD	1.9	3.7	1.4	2.8	2.4	4.0
**42**	average	41.1	41.4	25.9	4.6	12.2	39.1
	SD	0.9	4.4	0.9	3.0	0.0	3.1
**62**	average	65.9	91.5	31.5	8.7	26.6	50.8
	SD	3.2	2.3	17.5	0.7	0.8	0.7
**73**	average	73.9	105.9	48.7	9.1	28.1	51.0
	SD	2.5	6.2	4.5	0.4	0.9	0.6
**84**	average	93.8	107.5	49.1	8.3	29.0	52.6
	SD	14.3	6.3	5.0	2.3	1.1	0.8
**94**	average	113.6	122.5	55.3	9.9	30.2	54.6
	SD	10.9	5.3	3.5	0.4	1.4	1.4
**105**	average	101.2	131.7	64.1	10.7	31.0	53.0
	SD	9.3	9.5	5.0	0.6	0.9	0.6

Relative migration - Results in %. Average values

Migration time [days]	Toluene	Chloro-benzene	Styrene	Phenyl-cyclo-hexane	Benzo-phenone	Methyl-stearate
**1**	4.4	0.9		0.2	1.0	2.5
**2**	4.5	0.9		0.2	1.1	2.7
**3**	4.4	0.9		0.3	1.3	2.6
**7**	4.5	0.9		0.3	1.2	2.7

**10**	4.2	2.3		0.3	1.3	2.8
**14**	4.1	4.6		0.5	1.3	2.8
**21**	3.6	3.9	0.4	0.4	1.5	3.1
**28**	4.3	5.1	1.9	0.4	1.1	2.8
**35**	4.7	5.8	2.8	0.6	1.1	2.7
**42**	4.8	4.0	3.6	0.3	1.0	2.7
**62**	7.7	8.9	4.4	0.6	2.1	3.5
**73**	8.6	10.2	6.7	0.6	2.2	3.5
**84**	11.0	10.4	6.8	0.6	2.3	3.6
**94**	13.3	11.9	7.7	0.7	2.4	3.7
**105**	11.8	12.7	8.9	0.7	2.5	3.6

Migration Kinetics - Results in µg/dm². Average values

Migration time [days]	Oligo-1	Oligo-2	Oligo-3	Oligo-4	Oligo-5	Oligo-6	Oligo-7	Oligo-8
**1**								
**2**		2.8		2.4	7.8	2.0		
**3**		4.2		2.9	7.1	1.9		
**7**		4.1		3.3	9.0	2.2		
**10**		4.8		3.6	9.4	2.8		
**14**		4.7		3.6	10.3	2.8		
**21**		5.1	3.6	4.3	18.8	3.0		
**28**		5.2	7.0	4.4	7.0	3.9		
**35**		4.7	6.0	4.5	7.9	4.4		
**42**		6.6	11.4	4.8	10.4	4.3		
**62**	6.2	7.0	3.6	6.2	20.0	5.5		
**73**	2.4	7.8	5.4	6.6	16.8	5.6	1.27	
**84**	3.1	8.4	3.6	6.8	18.2	5.0	2.55	
**94**	3.1	8.6	3.7	6.5	17.6	5.2		
**105**	2.6	8.7	3.7	6.4	17.0	4.5		

Relative migration - Results in %. Average values

Migration time [days]	Oligo-1	Oligo-2	Oligo-3	Oligo-4	Oligo-5	Oligo-6	Oligo-7	Oligo-8
**1**	0.0	0.0		0.0	0.0	0.0	0.0	
**2**	0.0	0.2		0.1	0.2	0.2	0.0	
**3**	0.0	0.4		0.1	0.1	0.1	0.0	
**7**	0.0	0.4		0.1	0.2	0.2	0.0	
**10**	0.0	0.4		0.2	0.2	0.2	0.0	
**14**	0.0	0.4		0.2	0.2	0.2	0.0	
**21**	0.0	0.5	0.1	0.2	0.4	0.2	0.0	

**28**	0.0	0.5	0.2	0.2	0.1	0.3	0.0
**35**	0.0	0.4	0.2	0.2	0.2	0.3	0.0
**42**	0.0	0.6	0.4	0.2	0.2	0.3	0.0
**62**	1.7	0.6	0.1	0.3	0.4	0.4	0.0
**73**	0.6	0.7	0.2	0.3	0.4	0.4	0.0
**84**	0.8	0.7	0.1	0.3	0.4	0.4	0.0
**94**	0.9	0.8	0.1	0.3	0.4	0.4	0.0
**105**	0.7	0.8	0.1	0.3	0.4	0.3	0.0

Weight increase - Results in %. Average values and standard deviation (N=3)

Time contact [days]	Weight increase [%]	SD
**2**	0.7	0.04
**5**	1.1	0.14
**8**	1.2	0.22
**12**	1.4	0.09
**15**	1.6	0.08
**19**	1.7	0.09
**22**	1.8	0.03
**26**	1.8	0.07
**44**	2.4	0.10
**48**	2.4	0.04
**57**	2.5	0.04
**72**	2.7	0.07
**100**	2.8	0.04
**127**	2.9	0.05
**154**	3.0	0.07
**169**	3.0	0.02
**198**	3.1	0.03
**247**	3.2	0.05

7 Migration and weight increase in 50% ethanol at 60 °C

Migration Kinetics - Results in µg/dm². Average values and standard deviation (N=3)

Migration time [days]		Toluene	Chloro-benzene	Styrene	Phenyl-cyclo-hexane	Benzo-phenone	Methyl-stearate
**1**	average	40.5	116.5			11.6	
	SD	08.5	115.8			04.3	
**2**	average	61.4	74.6	11.4		12.2	
	SD	09.1	21.7	10.7		04.9	
**3**	average	103.9	109.1	20.5		18.5	
	SD	12.4	11.1	33.4		04.1	
**7**	average	128.7	183.4	59.2	0.2	26.4	
	SD	63.3	39.6	18.9	2.0	04.0	
**10**	average	233.4	276.5	99.6	3.8	27.6	
	SD	14.8	12.4	09.0	1.6	01.8	
**14**	average	269.2	323.3	125.5	7.1	30.7	
	SD	19.3	17.9	11.1	2.5	03.4	
**21**	average	288.5	350.4	176.0	14.3	36.2	
	SD	135.4	189.4	23.6	5.2	04.7	
**28**	average	357.4	480.9	210.7	17.1	41.3	
	SD	34.3	29.1	16.3	2.2	03.1	
**35**	average	327.1	456.3	192.4	15.7	42.2	
	SD	34.2	55.0	24.5	3.0	04.0	
**42**	average	197.0	283.9	115.7	11.1	55.1	
	SD	44.4	63.6	43.1	02.8	05.6	
**49**	average	292.9	455.3	201.5	24.6	55.3	
	SD	36.2	34.0	17.1	6.6	07.4	

Note: Due to analytical interferences. migration of methyl-stearate from GPPS in 50% ethanol at 60 °C can not be measured

Relative migration - Results in %. Average values

Migration time [days]	Toluene	Chloro-benzene	Styrene	Phenyl-cyclo-hexane	Benzo-phenone	Methyl-stearate
**1**	4.7				0.9	
**2**	7.2	7.2	1.6		1.0	
**3**	12.1	10.6	2.8		1.5	
**7**	15.1	17.8	8.2	0.0	2.1	
**10**	27.3	26.8	13.8	0.3	2.2	
**14**	31.5	31.3	17.4	0.5	2.5	
**21**	33.8	33.9	24.4	1.0	2.9	
**28**	41.8	46.5	29.2	1.2	3.3	
**35**	38.3	44.2	26.7	1.1	3.4	
**42**	23.0	27.5	16.0	0.8	4.4	
**49**	34.3	44.1	27.9	1.7	4.4	

Migration Kinetics - Results in µg/dm². Average values

Migration time [days]	Oligo-1	Oligo-2	Oligo-3	Oligo-4	Oligo-5	Oligo-6	Oligo-7	Oligo-8
**1**		4.0	6.9	10.1	11.3	13.5		
**2**		5.1	7.0	8.9	8.7	19.7		
**3**		3.8	7.7	5.3	11.2	18.9		
**7**		8.4	12.4	9.4	25.2	7.2		
**10**		9.5	8.0	10.0	23.1	6.7		
**14**		9.0	8.5	9.9	23.8	7.1		
**21**		9.9	8.1	10.2	23.9	7.5		
**28**		9.5	9.9	10.3	24.9	7.8		
**35**		9.5	9.9	11.8	28.0	9.0		
**42**		11.2	12.2	30.0	8.7	3.9		
**49**		9.1	11.2	27.5	9.1	3.0		

Relative migration - Results in %. Average values

Migration time [days]	Oligo-1	Oligo-2	Oligo-3	Oligo-4	Oligo-5	Oligo-6	Oligo-7	Oligo-8
**1**		0.4	0.2	0.4	0.2	1.0		
**2**		0.5	0.2	0.4	0.2	1.5		
**3**		0.3	0.3	0.2	0.2	1.4		
**7**		0.7	0.4	0.4	0.5	0.5		
**10**		0.8	0.3	0.4	0.5	0.5		
**14**		0.8	0.3	0.4	0.5	0.5		
**21**		0.9	0.3	0.5	0.5	0.6		
**28**		0.8	0.3	0.5	0.5	0.6		
**35**		0.8	0.3	0.5	0.6	0.7		
**42**		1.0	0.4	1.3	0.2	0.3		
**49**		0.8	0.4	1.2	0.2	0.2		

Weight increase - Results in %. Average values and standard deviation (N=3)

Time contact [days]	Weight increase [%]	SD
**0.02**	0.5	0.36
**0.04**	0.2	0.35
**0.08**	0.2	0.06
**0.13**	0.4	0.06
**0.17**	0.3	0.02
**0.25**	0.4	0.03
**0.38**	0.4	0.03
**1**	1.0	0.15
**2**	1.1	0.12
**5**	1.3	0.10
**8**	1.2	0.07
**12**	1.2	0.07
**15**	1.2	0.03
**19**	1.2	0.05
**22**	1.3	0.02
**26**	1.2	0.04
**44**	1.2	0.04
**48**	1.2	0.05
**57**	1.2	0.02
**72**	1.2	0.45
**100**	1.3	0.39
**127**	1.4	0.22

8 Migration and weight increase in 50% ethanol at 40 °C

Migration Kinetics - Results in µg/dm². Average values and standard deviation (N=3)

Migration time [days]		Toluene	Chloro-benzene	Styrene	Phenyl-cyclo-hexane	Benzo-phenone	Methyl-stearate
**0.2**	average					7.7	
	SD					0.0	
**0.4**	average					8.6	
	SD					2.3	
**1.0**	average					7.7	
	SD					0.0	
**2.0**	average	1.9				7.7	
	SD	0.0				0.0	
**3.0**	average	1.9				7.7	
	SD	0.0				0.0	
**7.0**	average	21.2	5.6			7.7	
	SD	6.8	11.1			0.0	
**14**	average	34.9	18.0			7.7	
	SD	11.7	14.8			0.0	
**21**	average	34.9	27.8			8.1	
	SD	0.0	8.5			0.9	
**28**	average	51.5	67.2	6.6		7.7	
	SD	38.8	12.1	8.0		0.0	
**35**	average	87.9	97.8	19.6		9.6	
	SD	7.4	8.1	12.0		2.5	
**42**	average	95.6	104.2	26.1		8.6	
	SD	13.5	17.3	5.0		1.8	
**49**	average	92.8	128.8	42.4		7.7	
	SD	9.1	22.4	8.0		0.0	
**59**	average	71.3	69.7	70.0		21.4	
	SD	7.4	116.2	153.6		07.7	
**76**	average	111.0	183.4	23.5		20.7	
	SD	9.1	24.3	8.7		02.7	
**87**	average	107.7	153.9	21.6		21.8	
	SD	9.1	48.3	8.2		2.3	
**98**	average	84.6	139.1	35.2		22.4	
	SD	56.1	60.4	24.5		1.2	
**108**	average	114.3	115.5	29.4		24.2	
	SD	13.8	8.1	6.9		1.5	
**119**	average	81.2	281.5	22.1		21.5	
	SD	13.8	87.0	9.3		1.5	

Note: Due to analytical interferences. migration of methylstearate and of phenylcylohexane from GPPS in 50% ethanol at 40 °C can not be measured.

Relative migration - Results in %. Average values

Migration time [days]	Toluene	Chloro-benzene	Styrene	Phenyl-cyclo-hexane	Benzo-phenone	Methyl-stearate
**0.2**	0.0	0.0	0.0		0.6	
**0.4**	0.0	0.0	0.0		0.7	
**1**	0.0	0.0	0.0		0.6	
**2**	0.2	0.0	0.0		0.6	
**3**	0.2	0.0	0.0		0.6	
**7**	2.5	0.5	0.0		0.6	
**14**	4.1	1.7	0.0		0.6	
**21**	4.1	2.7	0.0		0.6	
**28**	6.0	6.5	0.9		0.6	
**35**	10.3	9.5	2.7		0.8	
**42**	11.2	10.1	3.6		0.7	
**49**	10.9	12.5	5.9		0.6	
**59**	8.3	6.7	9.7		1.7	
**76**	13.0	17.8	3.3		1.7	
**87**	12.6	14.9	3.0		1.7	
**98**	9.9	13.5	4.9		1.8	
**108**	13.4	11.2	4.1		1.9	
**119**	9.5	27.2	3.1		1.7	

Migration of oligomers from GPPS into 50% ethanol at 40 °C was not detectable.

Weight increase - Results in %. Average values and standard deviation (N=3)

Time contact [days]	Weight increase [%]	SD
**0.16**	0.3	0.11
**0.33**	0.3	0.09
**1**	0.6	0.07
**2**	0.8	0.05
**5**	1.0	0.09
**8**	1.1	0.13
**12**	1.1	0.05
**15**	1.1	0.04
**19**	1.2	0.04
**22**	1.2	0.24
**26**	1.2	0.22
**44**	1.2	0.23
**48**	1.2	0.32
**57**	1.2	0.31
**72**	1.2	0.30
**100**	1.2	0.34
**127**	1.2	0.34

9 Weight increase in 50% ethanol at 20 °C

The migration remains below the detection limits.

Weight increase - Results in %. Average values and standard deviation (N=3)

Time contact [days]	Weight increase [%]	SD
**1**	0.5	0.14
**2**	0.6	0.12
**5**	0.6	0.07
**8**	0.7	0.04
**12**	0.7	0.01
**15**	0.8	0.01
**19**	0.9	0.01
**23**	0.9	0.01
**26**	0.9	0.11
**44**	1.0	0.10
**48**	1.0	0.09
**57**	1.0	0.06
**72**	1.1	0.05
**100**	1.1	0.04

10 Weight increase in 20% ethanol at 60. 40 and 20 °C

Weight increase at 60 °C - Results in %. Average values and standard deviation (N=3)

Time contact [days]	Weight increase [%]	SD
**2**	0.6	0.02
**5**	0.6	0.02
**11**	0.6	0.02
**16**	0.6	0.04
**19**	0.5	0.04
**45**	0.5	0.04
**60**	0.5	0.04
**89**	0.5	0.04
**138**	0.6	0.04

Weight increase at 40 °C - Results in %. Average values and standard deviation (N=3)

Time contact [days]	Weight increase [%]	SD
**2**	0.3	0.01
**5**	0.4	0.04
**11**	0.5	0.05
**16**	0.5	0.01
**19**	0.5	0.04
**45**	0.5	0.05
**60**	0.5	0.01
**89**	0.5	0.01
**138**	0.5	0.01

Weight increase at 20 °C - Results in %. Average values and standard deviation (N=3)

Time contact [days]	Weight increase [%]	SD
**2**	0.1	0.05
**5**	0.2	0.05
**11**	0.2	0.04
**16**	0.3	0.02
**19**	0.3	0.03
**45**	0.4	0.01
**60**	0.4	0.01
**89**	0.4	0.02
**138**	0.4	0.02

❖ HIPS

11 Migration and weight increase in isooctane at 60 °C

Migration Kinetics - Results in µg/dm². Average values and standard deviation (N=3)

Migration time [days]		Toluene	Chloro-benzene	Styrene	Phenyl-cyclo-hexane	Benzo-phenone	Methyl-stearate
**0.21**	average	1076.0	1141.6	549.8	907.0	1105.8	1461.4
	SD	20.7	26.2	14.5	471.6	75.6	44.2
**0.38**	average	1095.2	1163.4	564.1	1189.5	1234.3	1614.2
	SD	24.6	27.6	16.4	60.3	99.3	61.2
**1**	average	1100.0	1153.9	560.9	1232.6	1337.5	1724.7
	SD	22.4	19.7	9.4	31.7	63.9	59.2
**2**	average	1122.4	1182.9	576.0	1314.2	1428.1	1796.5
	SD	15.9	12.2	7.5	20.5	39.1	58.2
**3**	average	1137.1	1194.9	583.8	1335.5	1448.8	1801.4
	SD	29.32	15.17	6.28	20.26	23.06	33.26
**7**	average	1126.58	1172.59	571.92	1318.23	1485.59	1783.85
	SD	21.21	11.50	5.13	19.71	15.66	26.15
**14**	average	1121.75	1179.97	570.89	1303.31	1477.29	1787.78
	SD	14.93	16.76	7.75	16.96	14.02	19.18
**22**	average	1165.35	1035.29	504.21	1177.45	1400.02	1747.04
	SD	43.80	26.96	3.41	10.89	22.25	40.71
**29**	average	1167.31	1202.28	581.66	1306.31	1494.30	1726.06
	SD	11.30	11.53	3.50	16.83	19.21	77.13
**35**	average	1180.28	1230.66	602.96	1370.85	1577.38	1819.77
	SD	15.65	6.80	3.37	14.29	18.86	84.23
**43**	average	1152.37	1217.05	590.82	1324.44	1455.87	1449.62
	SD	18.23	19.92	7.76	24.15	90.97	291.23
**49**	average	1134.72	1205.23	600.22	1333.66	1393.66	1440.42
	SD	25.94	50.34	58.51	90.04	94.67	131.52

Relative migration - Results in %. Average values

Migration time [days]	Toluene	Chloro-benzene	Styrene	Phenyl-cyclo-hexane	Benzo-phenone	Methyl-stearate
**0.21**	71.2	70.2	76.5	37.3	55.7	62.6
**0.38**	72.5	71.5	78.5	48.9	62.2	69.1
**1**	72.8	71.0	78.1	50.6	67.4	73.9
**2**	74.3	72.7	80.2	54.0	72.0	77.0
**3**	75.3	73.5	81.3	54.9	73.0	77.2
**7**	74.6	72.1	79.6	54.2	74.9	76.4
**14**	74.3	72.6	79.5	53.6	74.5	76.6
**22**	77.1	63.7	70.2	48.4	70.6	74.8

**29**	77.3	73.9	81.0	53.7	75.3	73.9
**35**	78.1	75.7	83.9	56.3	79.5	77.9
**43**	76.3	74.8	82.2	54.4	73.4	62.1
**49**	75.1	74.1	83.6	54.8	70.3	61.7

Migration Kinetics - Results in μg/dm². Average values

Migration time [days]	Oligo-1	Oligo-2	Oligo-3	Oligo-4	Oligo-5	Oligo-6	Oligo-7	Oligo-8
**0.21**	119.3	1549.5	1913.3	4099.4	8384.4	2375.1	1007.4	308.5
**0.38**	134.5	1761.6	2155.1	4534.0	9342.0	2666.4	1135.7	353.4
**1**	143.7	1942.3	2617.6	5351.5	11226.9	3227.7	1378.3	428.6
**2**	153.4	2070.4	3075.4	6362.7	13259.7	3788.8	1610.9	498.3
**3**	154.2	2087.8	3256.4	6873.8	14117.9	3277.7	1728.2	527.5
**7**	140.5	1891.9	3157.6	6904.2	13810.3	3865.5	1657.4	489.0
**14**	143.3	1916.0	3297.5	7247.6	14381.9	4003.7	1720.1	505.1
**22**	138.5	1909.1	3498.2	7808.1	15501.2	4297.9	1859.8	541.8
**29**	141.7	1863.2	3198.2	6993.3	13865.8	3087.1	1670.3	497.0
**35**	141.6	1856.2	3136.7	6865.9	13585.7	3774.8	1640.8	492.3
**43**	145.1	1894.1	3240.7	7170.5	14194.6	3932.1	1714.0	509.9
**49**	152.0	1998.6	3296.4	7264.9	14495.1	4036.5	1670.8	499.3

Relative migration - Results in %. Average values

Migration time [days]	Oligo-1	Oligo-2	Oligo-3	Oligo-4	Oligo-5	Oligo-6	Oligo-7	Oligo-8
**0.21**	44.9	48.6	22.4	30.7	31.5	32.6	31.3	32.5
**0.38**	50.6	55.3	25.2	34.0	35.1	36.6	35.3	37.3
**1**	54.1	61.0	30.6	40.1	42.2	44.2	42.9	45.2
**2**	57.8	65.0	35.9	47.7	49.8	51.9	50.1	52.6
**3**	58.1	65.5	38.0	51.5	53.1	44.9	53.7	55.6
**7**	52.9	59.4	36.9	51.7	51.9	53.0	51.5	51.6
**14**	54.0	60.1	38.5	54.3	54.0	54.9	53.5	53.3
**22**	52.2	59.9	40.9	58.5	58.3	58.9	57.8	57.2
**29**	53.4	58.5	37.4	52.4	52.1	42.3	51.9	52.4
**35**	53.3	58.3	36.6	51.4	51.1	51.7	51.0	51.9
**43**	54.6	59.4	37.9	53.7	53.3	53.9	53.3	53.8
**49**	57.2	62.7	38.5	54.4	54.5	55.3	51.9	52.7

Weight increase - Results in %. Average values and standard deviation (N=3)

Time contact [days]	Weight increase [%]	SD
**0.2**	16.7	0.03
**0.3**	17.3	0.18
**1**	20.9	0.44
**2**	22.1	0.30
**5**	26.3	1.43
**12**	31.8	1.83
**16**	33.8	0.41
**30**	36.0	0.67
**44**	38.3	0.71
**64**	42.8	0.21
**92**	49.9	1.91
**126**	58.1	2.09
**159**	63.4	0.20
**201**	68.2	2.60

12 Migration and weight increase in isooctane at 40 °C

Migration Kinetics - Results in µg/dm². Average values and standard deviation (N=3)

Migration time [days]		Toluene	Chloro-benzene	Styrene	Phenyl-cyclo-hexane	Benzo-phenone	Methyl-stearate
**1**	average	1052.7	1064.0	526.0	655.6	710.8	922.7
	SD	508.3	18.7	13.0	11.0	8.8	9.3
**2**	average	1279.54	1083.19	558.97	817.99	897.11	1119.46
	SD	30.91	22.90	18.43	31.87	50.94	62.73
**3**	average	1300.51	1093.69	560.81	882.42	966.64	1219.50
	SD	33.92	31.36	28.63	19.53	15.58	20.24
**7**	average	1333.40	1108.12	588.64	1049.93	1170.68	1428.19
	SD	27.84	27.17	19.11	18.01	11.30	15.32
**14**	average	1325.85	1116.16	575.47	1179.41	1308.78	1609.87
	SD	18.28	20.93	29.03	33.41	33.00	41.04
**22**	average	1458.15	972.96	541.10	1094.57	1276.57	1688.49
	SD	58.06	14.98	9.21	21.75	18.84	31.26
**29**	average	1412.59	1155.20	612.93	1246.33	1391.45	1724.25
	SD	83.00	24.45	14.79	26.30	23.21	27.62
**35**	average	1357.98	1180.79	635.50	1307.97	1469.28	1808.45
	SD	16.85	20.15	10.88	29.64	22.37	26.72
**43**	average	1334.60	1138.80	601.36	1255.55	1385.92	1654.69
	SD	15.67	15.88	26.01	9.86	26.31	25.41
**49**	average	1315.44	1145.69	597.12	1267.78	1391.03	1597.35
	SD	28.30	19.37	37.06	25.92	22.04	46.02

Relative migration - Results in %. Average values

Migration time [days]	Toluene	Chloro-benzene	Styrene	Phenyl-cyclo-hexane	Benzo-phenone	Methyl-stearate
**1**	69.7	65.4	73.2	26.9	35.8	39.5
**2**	84.7	66.6	77.8	33.6	45.2	48.0
**3**	86.1	67.3	78.1	36.3	48.7	52.2
**7**	88.3	68.1	81.9	43.1	59.0	61.2
**14**	87.8	68.6	80.1	48.5	66.0	69.0
**22**	96.5	59.8	75.3	45.0	64.4	72.3
**29**	93.5	71.0	85.3	51.2	70.1	73.9
**35**	89.9	72.6	88.5	53.7	74.1	77.5
**43**	88.4	70.0	83.7	51.6	69.9	70.9
**49**	87.1	70.5	83.1	52.1	70.1	68.4

Migration Kinetics - Results in µg/dm². Average values

Migration time [days]	Oligo-1	Oligo-2	Oligo-3	Oligo-4	Oligo-5	Oligo-6	Oligo-7	Oligo-8
**1**	80.0	1029.8	1648.3	3519.5	7046.5	2000.8	856.3	256.7
**2**	96.2	1247.0	2054.4	4445.0	8873.5	2506.7	1075.8	318.1
**3**	108.9	1414.1	2390.1	5183.9	10331.0	2890.7	1250.7	370.6
**7**	117.5	1533.0	2624.3	5707.4	11312.1	3159.0	1357.5	400.9
**14**	132.1	1723.4	2988.2	6489.6	12845.3	3572.2	1503.7	448.3
**22**	132.3	1813.9	3349.6	7402.4	14668.9	4083.6	1759.1	520.8
**29**	135.2	1784.0	1554.0	6602.0	13081.8	3610.1	1339.6	463.4
**35**	136.6	1780.6	540.7	6474.7	12801.8	3551.0	1549.2	460.7
**43**	144.4	1810.4	3118.8	6824.1	13499.5	3742.4	1621.4	481.5
**49**	146.0	1819.3	3121.2	6812.4	13475.2	3742.0	1625.5	484.5

Relative migration - Results in %. Average values

Migration time [days]	Oligo-1	Oligo-2	Oligo-3	Oligo-4	Oligo-5	Oligo-6	Oligo-7	Oligo-8
**1**	30.1	32.3	19.3	26.4	26.5	27.4	26.6	27.1
**2**	36.2	39.1	24.0	33.3	33.3	34.4	33.4	33.6
**3**	41.0	44.4	27.9	38.8	38.8	39.6	38.9	39.1
**7**	44.3	48.1	30.7	42.8	42.5	43.3	42.2	42.3
**14**	49.7	54.1	34.9	48.6	48.3	49.0	46.8	47.3
**22**	49.8	56.9	39.1	55.5	55.1	56.0	54.7	54.9
**29**	50.9	56.0	35.0	49.5	49.2	49.5	41.7	48.9
**35**	51.4	55.9	35.0	48.5	48.1	48.7	48.2	48.6
**43**	54.4	56.8	36.4	51.1	50.7	51.3	50.4	50.8
**49**	55.0	57.1	36.5	51.0	50.6	51.3	50.5	51.1

Weight increase- Results in %. Average values and standard deviation (N=3)

Time contact [days]	Weight increase [%]	SD
**0.2**	3.1	0.22
**0.3**	2.4	0.57
**1**	5.6	0.55
**2**	6.9	0.40
**5**	8.7	1.37
**12**	12.4	1.36
**16**	13.2	0.33
**30**	14.0	0.73
**44**	14.4	0.73
**64**	14.4	0.43
**92**	14.3	1.17
**126**	14.2	0.77
**159**	14.5	0.61

13 Migration and weight increase in isooctane at 20 °C

Migration Kinetics - Results in µg/dm². Average values and standard deviation (N=3)

Migration time [days]		Toluene	Chloro-benzene	Styrene	Phenyl-cyclo-hexane	Benzo-phenone	Methyl-stearate
**1**	average	763.48	743.30	298.00	161.43	187.26	277.16
	SD	207.58	99.34	58.21	33.41	27.60	41.57
**2**	average	887.18	852.55	355.62	233.84	269.79	346.42
	SD	106.14	9.01	18.41	4.32	3.46	3.46
**3**	average	887.03	876.01	377.05	270.92	313.61	382.03
	SD	110.28	5.52	24.69	2.69	3.88	3.07
**7**	average	955.06	919.15	414.62	363.22	422.13	476.65
	SD	104.47	9.70	18.40	3.11	6.67	6.09
**14**	average	972.56	938.34	444.18	466.19	534.10	591.33
	SD	145.93	49.60	32.57	19.86	29.93	28.82
**22**	average	1144.53	901.76	428.83	476.76	554.97	685.64
	SD	129.33	55.21	26.40	12.90	12.70	20.63
**29**	average	1052.81	1009.37	472.48	567.09	636.53	740.26
	SD	103.23	8.98	4.46	9.03	9.50	11.17
**35**	average	1073.63	1045.79	503.07	624.89	695.42	801.07
	SD	109.49	6.44	22.46	12.86	10.60	16.15
**43**	average	1060.96	1006.91	478.66	640.22	672.75	738.30
	SD	92.28	10.09	5.00	11.60	15.86	17.91
**49**	average	1053.87	1036.60	493.67	670.06	722.79	773.61
	SD	114.44	5.38	3.48	8.99	9.35	21.50
**56**	average	1097.16	1092.87	517.27	728.38	742.84	603.40
	SD	102.62	75.68	38.37	57.53	39.56	80.96
**63**	average	1075.44	1042.51	496.77	707.35	752.51	761.54
	SD	106.45	3.67	4.28	8.31	7.07	10.33
**70**	average	1076.80	1035.78	501.69	722.06	733.85	476.46
	SD	103.74	26.27	26.29	21.58	50.55	102.79
**77**	average	1071.22	1027.09	491.50	742.67	746.71	677.34
	SD	95.81	4.90	4.09	15.81	10.16	30.25
**84**	average	1084.79	1084.34	528.73	812.29	803.94	762.38
	SD	98.53	84.20	39.83	77.23	56.28	67.16
**91**	average	1073.48	1046.61	513.49	800.48	818.45	888.29
	SD	106.66	9.14	21.53	20.02	13.07	14.49
**98**	average	1062.77	1025.61	487.71	752.93	806.01	849.96
	SD	100.04	12.99	5.35	22.12	22.52	66.97
**105**	average	1074.23	1031.52	493.56	769.61	820.25	945.78
	SD	93.92	19.11	8.20	21.60	25.57	26.41
**115**	average	1101.54	1048.58	501.35	790.74	846.79	924.81
	SD	107.20	21.53	10.30	32.14	31.19	82.88

Relative migration - Results in %. Average values

Migration time [days]	Toluene	Chloro-benzene	Styrene	Phenyl-cyclo-hexane	Benzo-phenone	Methyl-stearate
**1**	50.5	45.7	41.5	6.6	9.4	11.9
**2**	58.7	52.4	49.5	9.6	13.6	14.8
**3**	58.7	53.9	52.5	11.1	15.8	16.4
**7**	63.2	56.5	57.7	14.9	21.3	20.4
**14**	64.4	57.7	61.8	19.2	26.9	25.3
**22**	75.8	55.5	59.7	19.6	28.0	29.4
**29**	69.7	62.1	65.8	23.3	32.1	31.7
**35**	71.1	64.3	70.0	25.7	35.1	34.3
**43**	70.2	61.9	66.6	26.3	33.9	31.6
**49**	69.8	63.7	68.7	27.5	36.4	33.1
**56**	72.6	67.2	72.0	29.9	37.4	25.8
**63**	71.2	64.1	69.2	29.1	37.9	32.6
**70**	71.3	63.7	69.8	29.7	37.0	20.4
**77**	70.9	63.2	68.4	30.5	37.6	29.0
**84**	71.8	66.7	73.6	33.4	40.5	32.7
**91**	71.1	64.4	71.5	32.9	41.3	38.0
**98**	70.4	63.1	67.9	30.9	40.6	36.4
**105**	71.1	63.4	68.7	31.6	41.4	40.5
**115**	72.9	64.5	69.8	32.5	42.7	39.6

Migration Kinetics - Results in µg/dm^2. Average values

Migration time [days]	Oligo-1	Oligo-2	Oligo-3	Oligo-4	Oligo-5	Oligo-6	Oligo-7	Oligo-8
**1**	22.6	218.1	588.6	1155.0	2360.9	688.7	280.6	87.1
**2**	27.3	296.4	646.9	1301.1	2671.3	775.5	312.5	98.2
**3**	31.5	334.5	643.5	1293.7	2657.6	776.9	323.2	98.7
**7**	36.6	420.3	635.6	1257.2	2593.2	752.7	317.0	97.7
**14**	48.2	552.1	683.9	1400.9	2914.0	848.5	361.6	112.0
**22**	52.5	657.4	853.1	1670.4	3505.8	1021.9	436.1	134.9
**29**	62.8	712.1	874.0	1652.7	3482.8	1008.5	435.5	136.3
**35**	63.9	745.4	881.9	1659.0	3501.9	1019.7	443.6	140.2
**43**	68.6	791.2	936.6	1772.9	3771.6	1091.3	479.9	149.0
**49**	74.0	817.0	974.0	1827.4	3895.0	1130.0	498.2	155.7
**56**	68.9	885.1	983.8	1923.3	4189.6	1205.4	536.7	170.5
**63**	72.5	901.9	1024.8	2031.7	4352.6	1251.7	563.1	176.0
**70**	76.5	987.6	1157.0	2343.4	5047.4	1453.5	656.3	207.2
**77**	79.0	1003.5	1186.0	2369.4	5082.3	1454.4	657.3	205.8
**84**	75.4	998.4	1124.6	2240.9	4823.7	1387.4	622.3	194.9
**91**	78.2	1000.4	1172.4	2334.2	4999.9	1440.7	637.2	197.9

**98**	79.1	999.2	1253.2	2515.3	5399.1	1554.5	694.0	218.8
**105**	81.4	1030.8	1321.4	2629.2	5643.3	1630.2	723.7	229.6
**115**	84.5	1074.3	1373.1	2729.6	5885.3	1689.9	764.2	243.4

Relative migration - Results in %. Average values

Migration time [days]	Oligo-1	Oligo-2	Oligo-3	Oligo-4	Oligo-5	Oligo-6	Oligo-7	Oligo-8
**1**	8.5	6.8	6.9	8.7	8.9	9.4	8.7	9.2
**2**	10.3	9.3	7.6	9.7	10.0	10.6	9.7	10.4
**3**	11.9	10.5	7.5	9.7	10.0	10.7	10.0	10.4
**7**	13.8	13.2	7.4	9.4	9.7	10.3	9.9	10.3
**14**	18.1	17.3	8.0	10.5	11.0	11.6	11.2	11.8
**22**	19.8	20.6	10.0	12.5	13.2	14.0	13.6	14.2
**29**	23.6	22.4	10.2	12.4	13.1	13.8	13.5	14.4
**35**	24.0	23.4	10.3	12.4	13.2	14.0	13.8	14.8
**43**	25.8	24.8	10.9	13.3	14.2	15.0	14.9	15.7
**49**	27.8	25.6	11.4	13.7	14.6	15.5	15.5	16.4
**56**	25.9	27.8	11.5	14.4	15.7	16.5	16.7	18.0
**63**	27.3	28.3	12.0	15.2	16.4	17.2	17.5	18.6
**70**	28.8	31.0	13.5	17.6	19.0	19.9	20.4	21.9
**77**	29.7	31.5	13.9	17.8	19.1	19.9	20.4	21.7
**84**	28.4	31.3	13.1	16.8	18.1	19.0	19.3	20.6
**91**	29.4	31.4	13.7	17.5	18.8	19.8	19.8	20.9
**98**	29.8	31.4	14.6	18.8	20.3	21.3	21.6	23.1
**105**	30.7	32.4	15.4	19.7	21.2	22.3	22.5	24.2
**115**	31.8	33.7	16.0	20.5	22.1	23.2	23.8	25.7

Weight increase - Results in %. Average values and standard deviation (N=3)

Time contact [days]	Weight increase [%]	SD
**2**	1.6	0.19
**5**	2.3	0.40
**12**	2.7	0.43
**16**	2.9	0.05
**30**	3.5	0.11
**44**	4.0	0.19
**64**	4.4	0.13
**92**	5.0	0.18
**126**	5.6	0.17
**159**	6.2	0.33
**201**	7.0	0.43
**273**	7.6	0.50

14 Migration and weight increase in 95% ethanol at 60 °C

Migration Kinetics - Results in µg/dm². Average values and standard deviation (N=3)

Migration time [days]		Toluene	Chloro-benzene	Styrene	Phenyl-cyclo-hexane	Benzo-phenone	Methyl-stearate
**0.33**	average	100.5	181.0	53.4	37.1	71.4	53.9
	SD	14.2	28.7	6.2	1.5	3.8	5.5
**1**	average	313.6	426.9	163.3	53.7	118.2	76.6
	SD	34.7	29.4	12.1	23.6	6.3	4.1
**2**	average	675.2	729.2	285.7	96.1	173.5	99.5
	SD	183.8	65.7	26.7	5.8	9.4	6.6
**3**	average	785.0	894.8	367.8	188.2	221.1	141.5
	SD	33.6	55.1	17.2	07.2	10.2	7.2
**7**	average	1164.1	1239.7	548.7	274.5	361.3	203.4
	SD	34.8	28.4	12.3	09.8	15.1	11.8
**14**	average	1306.4	1400.5	659.5	379.5	528.0	274.9
	SD	27.3	20.6	08.3	17.7	27.7	18.0
**22**	average	1392.9	1750.3	834.5	514.5	742.4	301.8
	SD	150.3	137.8	25.1	27.5	31.3	16.5
**29**	average	1307.0	1388.7	670.5	522.7	747.8	440.9
	SD	20.9	29.5	12.3	30.6	42.4	33.2
**35**	average	1390.0	1454.5	751.3	649.9	940.3	542.0
	SD	24.9	34.5	05.8	41.5	66.3	52.8
**43**	average	1251.9	1451.7	733.4	710.0	968.3	443.7
	SD	38.9	30.6	14.2	31.1	61.4	72.6
**49**	average	1293.8	1488.5	751.8	768.5	1073.5	495.2
	SD	52.5	21.9	09.7	55.9	80.8	78.8
**56**	average	1171.5	1420.3	728.9	798.1	1136.0	487.9
	SD	46.4	45.5	09.9	48.9	56.9	43.5
**63**	average	1200.0	1452.2	741.5	866.7	1200.0	496.7
	SD	75.1	55.2	15.1	43.0	51.7	39.5
**70**	average	1203.3	1348.0	748.9	935.6	1262.6	423.5
	SD	44.6	116.2	17.7	69.0	71.2	43.4
**77**	average	1181.0	1424.0	744.6	965.8	1289.9	444.1
	SD	48.2	40.1	17.8	61.0	54.8	29.0
**84**	average	1230.7	1357.9	743.0	1006.5	1422.3	710.1
	SD	55.6	78.6	20.0	87.6	80.9	33.8
**91**	average	1199.0	1410.4	708.5	989.2	1427.8	767.6
	SD	56.9	22.1	12.1	36.5	39.2	42.1
**98**	average	1075.1	1354.4	725.2	1111.8	1536.5	429.1
	SD	99.1	57.9	36.5	74.9	69.7	241.8
**105**	average	997.2	1272.0	647.6	1066.3	1497.4	644.1
	SD	166.3	119.0	53.4	105.8	97.0	19.5
**115**	average	1250.7	1407.3	735.0	1194.4	1591.4	891.8
	SD	53.6	51.8	13.2	49.5	34.9	46.4

Relative migration - Results in %. Average values

Migration time [days]	Toluene	Chloro-benzene	Styrene	Phenyl-cyclo-hexane	Benzo-phenone	Methyl-stearate
**0.33**	6.7	11.1	7.4	1.5	3.6	2.3
**1**	20.8	26.3	22.7	2.2	6.0	3.3
**2**	20.8	44.8	39.8	3.9	8.7	4.3
**3**	52.0	55.0	50.4	5.2	11.1	5.5
**7**	77.1	76.2	76.1	8.8	18.2	8.4
**14**	86.5	86.1	91.8	13.3	26.6	11.7
**22**	92.2	83.0	88.8	19.1	37.5	13.0
**29**	86.5	85.4	93.4	19.4	37.7	19.5
**35**	92.0	89.4	104.8	24.8	47.5	24.2
**43**	82.9	89.3	102.3	27.4	48.9	19.6
**49**	85.7	91.5	104.9	29.9	54.2	22.0
**56**	77.6	87.3	101.6	31.1	57.4	21.7
**63**	79.4	89.3	103.4	34.1	60.6	22.1
**70**	79.7	82.9	104.5	37.0	63.8	18.7
**77**	78.2	87.6	103.9	38.3	65.2	19.6
**84**	81.5	83.5	103.6	40.0	71.8	32.1
**91**	79.4	86.7	98.7	39.3	72.1	34.8
**98**	71.2	83.3	101.1	44.5	77.6	18.9
**105**	66.0	78.2	90.1	42.6	75.6	29.0
**115**	82.8	86.5	102.5	48.0	80.4	40.6

Migration Kinetics - Results in µg/dm^2. Average values

Migration time [days]	Oligo-1	Oligo-2	Oligo-3	Oligo-4	Oligo-5	Oligo-6	Oligo-7	Oligo-8
**0.33**	4.48	27.29	49.55	30.06	68.18	20.98	8.90	3.39
**1**	5.88	39.38	46.63	35.55	82.43	24.65	10.42	4.03
**2**	8.74	55.15	43.89	39.76	94.07	27.24	11.23	5.08
**3**	11.69	69.99	47.13	46.13	111.83	32.62	14.51	6.28
**7**	15.58	113.44	53.05	58.82	139.11	41.96	19.19	7.11
**14**	22.17	164.14	57.65	73.20	177.28	54.90	26.51	9.62
**22**	24.71	202.94	54.36	90.53	218.33	65.91	31.93	12.13
**29**	31.88	236.57	112.52	95.56	234.26	62.31	36.20	10.61
**35**	36.37	259.20	105.18	96.78	239.01	72.28	39.81	2.32
**43**	13.36	299.14	109.79	108.85	264.15	45.03	45.02	16.67
**49**	45.83	326.16	115.99	114.99	283.28	3.93	41.71	16.63
**56**	18.48	344.90	87.46	118.41	295.96	91.86	48.03	17.27
**63**	24.05	369.47	86.65	123.12	305.07	94.00	24.06	18.75
**70**	8.97	403.49	100.98	137.31	337.04	103.95	55.92	20.83
**77**	14.30	423.06	98.99	143.96	354.98	108.94	57.34	20.96

**84**	17.67	446.39	126.32	158.76	392.76	120.61	63.21	23.75
**91**	7.82	467.29	129.62	182.31	451.28	139.10	72.00	27.15
**98**	8.25	491.17	114.83	164.48	406.55	125.26	62.13	23.58
**105**	6.63	507.92	120.20	174.95	436.56	136.33	70.22	27.58
**115**	7.27	541.09	128.03	178.07	443.08	137.70	72.58	28.14

Relative migration - Results in %. Average values

Migration time [days]	Oligo-1	Oligo-2	Oligo-3	Oligo-4	Oligo-5	Oligo-6	Oligo-7	Oligo-8
**0.33**	1.7	0.9	0.6	0.2	0.3	0.3	0.3	0.4
**1**	2.2	1.2	0.5	0.3	0.3	0.3	0.3	0.4
**2**	3.3	1.7	0.5	0.3	0.4	0.4	0.3	0.5
**3**	4.4	2.2	0.6	0.3	0.4	0.4	0.5	0.7
**7**	5.9	3.6	0.6	0.4	0.5	0.6	0.6	0.7
**14**	8.3	5.2	0.7	0.5	0.7	0.8	0.8	1.0
**22**	9.3	6.4	0.6	0.7	0.8	0.9	1.0	1.3
**29**	12.0	7.4	1.3	0.7	0.9	0.9	1.1	1.1
**35**	13.7	8.1	1.2	0.7	0.9	1.0	1.2	0.2
**43**	5.0	9.4	1.3	0.8	1.0	0.6	1.4	1.8
**49**	17.3	10.2	1.4	0.9	1.1	0.1	1.3	1.8
**56**	7.0	10.8	1.0	0.9	1.1	1.3	1.5	1.8
**63**	9.1	11.6	1.0	0.9	1.1	1.3	0.7	2.0
**70**	3.4	12.7	1.2	1.0	1.3	1.4	1.7	2.2
**77**	5.4	13.3	1.2	1.1	1.3	1.5	1.8	2.2
**84**	6.7	14.0	1.5	1.2	1.5	1.7	2.0	2.5
**91**	2.9	14.7	1.5	1.4	1.7	1.9	2.2	2.9
**98**	3.1	15.4	1.3	1.2	1.5	1.7	1.9	2.5
**105**	2.5	15.9	1.4	1.3	1.6	1.9	2.2	2.9
**115**	2.7	17.0	1.5	1.3	1.7	1.9	2.3	3.0

Weight increase - Results in %. Average values and standard deviation (N=3)

Time contact [days]	Weight increase [%]	SD
**0.2**	1.6	0.06
**0.3**	1.9	0.11
**1**	3.2	0.17
**2**	3.3	0.05
**5**	3.2	0.58
**12**	3.2	0.62
**16**	3.2	0.05
**30**	3.3	0.05
**44**	3.7	0.07
**64**	4.2	0.03
**92**	5.4	0.09
**126**	7.4	0.06
**159**	9.8	0.04
**201**	16.2	0.01
**273**	34.0	0.06

15 Migration and weight increase in 95% ethanol at 40 °C

Migration Kinetics - Results in µg/dm². Average values and standard deviation (N=3)

Migration time [days]		Toluene	Chloro-benzene	Styrene	Phenyl-cyclo-hexane	Benzo-phenone	Methyl-stearate
**1**	average	51.0	98.8	37.8	11.3	35.6	29.7
	SD	1.9	24.0	3.6	8.2	5.5	1.3
**2**	average	78.6	211.2	66.0	19.6	39.0	34.5
	SD	1.7	39.3	12.9	1.4	2.7	0.9
**3**	average	133.5	283.7	79.0	23.3	47.2	37.4
	SD	6.2	10.5	14.2	0.8	4.2	3.5
**7**	average	236.6	390.2	123.4	30.9	64.8	42.3
	SD	6.8	50.8	29.1	1.5	4.5	2.0
**14**	average	364.9	590.2	200.0	40.7	83.9	45.6
	SD	11.8	49.4	27.4	1.5	5.5	1.2
**22**	average	445.9	916.0	301.9	55.6	106.7	57.8
	SD	62.2	47.4	14.9	2.0	2.1	4.6
**29**	average	609.0	792.2	276.5	56.4	113.4	75.9
	SD	11.0	10.3	19.0	1.3	5.6	2.8
**35**	average	707.0	913.9	330.2	67.6	131.2	88.5
	SD	10.5	18.9	7.5	3.4	3.7	4.7
**43**	average	697.3	984.1	366.0	72.9	104.9	33.0
	SD	19.0	14.4	8.3	2.7	13.4	7.7
**49**	average	764.8	1067.3	395.1	77.4	132.4	45.4
	SD	19.4	14.4	6.8	2.3	9.3	7.6
**56**	average	811.3	1155.0	442.4	79.8	132.4	55.1
	SD	31.5	57.0	18.3	3.3	9.3	5.0
**63**	average	871.3	992.7	447.8	83.2	142.7	48.7
	SD	46.4	475.3	20.4	1.5	10.6	1.7
**70**	average	908.7	1188.3	483.1	76.1	150.6	40.0
	SD	20.8	47.0	46.3	31.8	7.3	4.2
**77**	average	990.4	1200.3	493.6	73.7	106.3	33.6
	SD	78.0	20.5	33.1	37.6	78.7	10.1
**84**	average	1095.2	1227.3	511.5	90.5	164.3	71.2
	SD	70.5	34.0	42.3	3.5	4.9	03.9
**91**	average	1080.5	1269.4	505.9	100.4	141.0	60.7
	SD	41.8	31.7	20.9	11.6	67.3	05.3
**98**	average	995.1	1239.3	498.5	102.4	178.1	63.8
	SD	70.4	34.2	11.6	7.4	6.6	4.9
**105**	average	991.2	1200.0	497.4	98.4	186.8	62.3
	SD	103.4	76.3	30.9	13.8	14.3	5.1
**115**	average	1168.5	1303.2	563.0	113.1	172.9	104.4
	SD	27.7	20.5	17.6	10.4	82.5	6.2
**133**	average	1160.4	1296.1	578.7	114.2	212.1	89.4
	SD	41.8	17.3	18.6	10.1	13.9	10.1

**147**	average	1164.2	1299.5	557.2	122.3	179.8	90.6
	SD	30.7	76.1	27.0	10.4	86.5	8.0
**161**	average	1156.3	1363.4	604.0	127.6	198.9	15.3
	SD	25.3	26.7	15.0	12.5	15.6	3.0
**175**	average	1203.7	1375.7	619.7	140.5	208.7	20.8
	SD	16.5	19.1	7.2	15.2	12.3	12.9
**189**	average	1217.2	1352.1	605.1	129.0	249.2	13.2
	SD	63.0	52.7	27.7	15.1	22.2	0.0
**202**	average	1264.2	1385.8	626.8	121.6	246.5	13.2
	SD	36.0	19.2	15.2	39.9	30.9	0.0

Relative migration - Results in %. Average values

**Migration time [days]**	**Toluene**	**Chloro-benzene**	**Styrene**	**Phenyl-cyclo-hexane**	**Benzo-phenone**	**Methyl-stearate**
**1**	3.4	6.1	5.3	0.5	1.8	1.3
**2**	5.2	13.0	9.2	0.8	2.0	1.5
**3**	8.8	17.4	11.0	1.0	2.4	1.6
**7**	15.7	24.0	17.2	1.3	3.3	1.8
**14**	24.2	36.3	27.8	1.7	4.2	2.0
**22**	29.5	56.3	42.0	2.3	5.4	2.5
**29**	40.1	49.5	37.8	2.3	5.8	3.3
**35**	46.5	57.2	45.2	2.7	6.7	3.8
**43**	45.9	61.6	50.2	2.9	5.3	1.4
**49**	50.3	66.9	54.3	3.1	6.8	1.9
**56**	53.3	72.4	60.9	3.2	7.3	2.4
**63**	57.3	62.2	61.6	3.4	7.6	2.1
**70**	59.7	74.5	66.5	3.1	7.7	1.7
**77**	65.1	75.3	68.0	3.6	8.2	1.9
**84**	71.9	77.0	70.5	3.7	8.4	3.0
**91**	70.9	79.7	69.7	4.1	8.8	2.6
**98**	65.4	77.8	68.7	4.2	9.2	2.7
**105**	65.1	75.3	68.5	4.0	9.6	2.7
**115**	76.7	81.8	77.6	4.6	10.6	4.5
**133**	76.2	81.4	79.8	4.7	10.9	3.8
**147**	76.4	81.6	76.8	5.0	11.0	3.9
**161**	75.9	85.6	83.3	5.2	10.3	0.7
**175**	79.0	86.4	85.5	5.8	10.8	0.9
**189**	79.9	84.9	83.5	5.3	12.9	0.6
**202**	83.0	87.1	86.5	5.0	12.8	0.6

Migration Kinetics - Results in μg/dm². Average values

Migration time [days]	Oligo-1	Oligo-2	Oligo-3	Oligo-4	Oligo-5	Oligo-6	Oligo-7	Oligo-8
**1**	n.d.	18.6	23.7	24.4	53.4	15.3	05.9	n.d.
**2**	n.d.	21.2	23.6	26.4	60.4	17.5	05.7	02.9
**3**	n.d.	24.3	24.6	20.3	65.2	20.4	06.8	01.6
**7**	n.d.	27.7	24.8	32.1	72.5	22.5	09.1	n.d.
**14**	n.d.	32.1	25.3	35.1	79.0	24.0	07.6	n.d.
**22**	n.d.	34.8	21.5	39.9	88.5	27.0	11.0	n.d.
**29**	06.0	39.0	61.3	38.5	88.0	27.2	04.0	n.d.
**35**	07.7	41.9	55.7	38.4	87.2	27.0	12.1	04.2
**43**	n.d.	42.0	52.7	38.7	85.7	16.7	13.8	n.d.
**49**	07.0	44.4	57.7	39.8	88.7	11.3	14.6	04.4
**56**	n.d.	45.0	28.5	39.9	91.1	27.4	13.5	n.d.
**63**	n.d.	46.1	23.4	40.5	91.0	26.7	13.1	04.0
**70**	n.d.	46.9	28.5	41.7	93.9	28.5	14.5	n.d.
**77**	n.d.	49.3	27.3	43.7	97.4	29.8	15.3	n.d.
**84**	06.8	52.9	26.6	43.3	96.8	29.0	12.6	n.d.
**91**	n.d.	50.9	30.9	43.5	96.7	29.6	13.9	n.d.
**98**	06.3	54.9	28.6	48.6	108.0	33.1	12.0	04.7
**105**	n.d.	55.7	33.2	49.3	110.0	34.0	11.9	05.0
**115**	08.9	58.8	34.9	48.2	108.8	33.3	12.6	05.2

Relative migration - Results in %. Average values

Migration time [days]	Oligo-1	Oligo-2	Oligo-3	Oligo-4	Oligo-5	Oligo-6	Oligo-7	Oligo-8
**1**		0.6	0.3	0.2	0.2	0.2	0.2	
**2**		0.7	0.3	0.2	0.2	0.2	0.2	0.3
**3**		0.8	0.3	0.2	0.2	0.3	0.2	0.2
**7**		0.9	0.3	0.2	0.3	0.3	0.3	
**14**		1.0	0.3	0.3	0.3	0.3	0.2	
**22**		1.1	0.3	0.3	0.3	0.4	0.3	
**29**	2.3	1.2	0.7	0.3	0.3	0.4	0.1	
**35**		1.3	0.7	0.3	0.3	0.4	0.4	0.4
**43**		1.3	0.6	0.3	0.3	0.2	0.4	
**49**	2.6	1.4	0.7	0.3	0.3	0.2	0.5	0.5
**56**		1.4	0.3	0.3	0.3	0.4	0.4	
**63**		1.4	0.3	0.3	0.3	0.4	0.4	0.4
**70**		1.5	0.3	0.3	0.4	0.4	0.4	
**77**		1.5	0.3	0.3	0.4	0.4	0.5	
**84**	2.6	1.7	0.3	0.3	0.4	0.4	0.4	
**91**		1.6	0.4	0.3	0.4	0.4	0.4	
**98**	2.4	1.7	0.3	0.4	0.4	0.5	0.4	0.5

**105**	1.7	0.4	0.4	0.4	0.5	0.4	0.5
**115**	1.8	0.4	0.4	0.4	0.5	0.4	0.6

Weight increase - Results in %. Average values and standard deviation (N=3)

Time contact [days]	Weight increase [%]	SD
**0.17**	0.62	0.07
**0.33**	0.72	0.09
**1**	1.58	0.05
**2**	1.89	0.06
**5**	2.55	0.49
**12**	2.74	0.40
**16**	2.79	0.13
**30**	2.68	0.25
**44**	2.68	0.16
**64**	2.59	0.13
**92**	2.46	0.42
**126**	2.48	0.30
**159**	2.50	0.12

16 Migration and weight increase in 95% ethanol at 20 °C

Migration Kinetics - Results in µg/dm². Average values and standard deviation (N=3)

Migration time [days]		Toluene	Chloro-benzene	Styrene	Phenyl-cyclo-hexane	Benzo-phenone	Methyl-stearate
**1**	average	25.8	30.4	17.3	3.5	18.7	26.0
	SD	0.9	2.2	1.2	0.4	2.6	1.0
**2**	average	23.2	46.8	19.4	5.1	23.9	27.0
	SD	1.9	4.8	1.2	85.0	4.2	0.9
**3**	average	25.6	52.7	20.7	6.5	25.8	28.3
	SD	3.3	5.0	1.9	3.9	2.5	2.1
**7**	average	36.7	73.3	25.9	13.3	29.5	29.9
	SD	0.6	1.3	1.5	0.5	4.1	1.7
**14**	average	53.6	106.9	37.3	16.3	32.6	27.4
	SD	0.6	1.9	2.1	0.8	4.9	0.7
**22**	average	35.3	148.7	57.6	21.8	40.1	31.0
	SD	2.6	23.3	11.5	1.5	2.3	1.0
**29**	average	80.6	223.6	56.7	26.4	37.6	37.4
	SD	2.3	6.8	1.8	5.3	1.4	1.9
**35**	average	113.9	217.6	67.4	28.0	43.5	43.1
	SD	13.4	22.2	1.9	2.2	5.4	0.9
**43**	average	101.4	222.8	76.8	34.6	32.2	
	SD	3.6	4.8	2.0	14.6	6.1	
**49**	average	117.8	253.4	87.4	32.2	36.1	
	SD	4.6	4.9	1.6	1.8	6.9	
**56**	average	136.7	279.6	98.8	31.5	41.2	
	SD	2.5	2.9	1.1	0.5	4.5	
**63**	average	166.4	300.3	106.8	32.6	42.3	
	SD	28.8	5.8	1.4	0.8	5.2	
**70**	average	167.2	304.5	112.9	33.1	42.3	
	SD	03.7	10.5	03.5	01.8	06.1	
**77**	average	174.9	334.6	120.0	33.4	49.9	
	SD	05.3	04.6	04.5	01.5	02.8	
**84**	average	225.8	349.2	124.3	33.9	42.8	
	SD	03.7	13.9	02.8	01.7	04.7	
**91**	average	239.1	382.5	131.0	32.7	45.2	
	SD	04.3	07.4	02.9	02.2	06.1	
**98**	average	216.9	371.8	126.3	33.2	45.5	
	SD	15.8	12.6	05.2	02.7	06.5	
**105**	average	268.0	415.2	143.3	35.6	54.0	
	SD	07.3	08.8	04.1	03.0	09.9	
**115**	average	292.7	437.2	158.3	39.9	59.2	
	SD	17.4	13.1	03.3	03.1	04.1	
**133**	average	334.2	467.0	165.0	43.1	60.9	
	SD	6.7	11.7	4.5	5.4	6.9	

Relative migration - Results in %. Average values

Migration time [days]	Toluene	Chloro-benzene	Styrene	Phenyl-cyclo-hexane	Benzo-phenone	Methyl-stearate
**1**	1.7	1.9	2.4	0.1	0.9	1.1
**2**	1.5	2.9	2.7	0.2	1.2	1.2
**3**	1.7	3.2	2.9	0.3	1.3	1.2
**7**	2.4	4.5	3.6	0.5	1.5	1.3
**14**	3.6	6.6	5.2	0.7	1.6	1.2
**22**	2.3	9.1	8.0	0.9	2.0	1.3
**29**	5.5	13.4	7.2	1.0	1.9	1.6
**35**	7.8	13.0	8.7	1.0	2.2	1.8
**43**	7.0	13.3	10.0	1.3	1.6	
**49**	8.1	15.2	11.5	1.2	1.8	
**56**	9.4	16.9	13.1	1.2	2.1	
**63**	11.5	18.2	14.2	1.2	2.1	
**70**	11.5	18.4	15.0	1.2	2.1	
**77**	12.1	20.3	16.0	1.3	2.5	
**84**	15.6	21.2	16.6	1.3	2.2	
**91**	16.5	23.3	17.5	1.2	2.3	
**98**	15.0	22.6	16.9	1.2	2.3	
**105**	18.5	25.3	19.2	1.4	2.7	
**115**	20.2	26.7	21.3	1.5	3.0	
**133**	23.1	28.5	22.2	1.7	3.1	

Migration Kinetics - Results in µg/dm². Average values

Migration time [days]	Oligo-1	Oligo-2	Oligo-3	Oligo-4	Oligo-5	Oligo-6	Oligo-7	Oligo-8
**1**	n.d.	09.7	24.8	17.0	39.4	11.8	05.7	n.d.
**2**	n.d.	11.6	20.7	19.8	44.2	12.7	05.2	n.d.
**3**	n.d.	14.5	19.8	22.4	47.5	15.6	06.2	n.d.
**7**	n.d.	17.1	17.9	23.9	52.5	16.2	06.4	n.d.
**14**	n.d.	20.4	18.4	27.5	59.9	18.5	06.1	n.d.
**22**	n.d.	21.5	16.5	31.4	67.5	20.2	08.1	n.d.
**29**	03.8	24.9	47.6	31.5	68.0	20.9	07.7	n.d.
**35**	n.d.	26.2	45.5	33.7	60.4	30.9	11.2	02.1
**43**	n.d.	24.2	41.3	33.2	69.5	20.2	11.3	n.d.
**49**	n.d.	25.2	47.2	27.8	64.3	29.4	11.9	n.d.
**56**	n.d.	25.9	18.7	35.3	79.7	25.7	12.2	04.6
**63**	n.d.	27.4	21.1	23.6	62.0	22.6	10.9	n.d.
**70**	n.d.	28.3	21.9	36.7	79.9	23.8	12.3	n.d.
**77**	n.d.	27.7	21.7	36.7	79.8	24.5	12.1	n.d.
**84**	n.d.	29.4	23.4	37.9	83.5	24.9	13.0	n.d.
**91**	n.d.	30.4	23.6	26.8	86.0	25.9	12.0	n.d.
**98**	n.d.	29.8	28.2	40.4	87.6	26.5	12.3	n.d.
**105**	n.d.	30.9	31.1	42.8	93.7	29.9	10.0	03.9
**115**	n.d.	31.7	25.9	40.7	89.4	27.1	11.9	04.0
**133**			n.d.	41.5	100.1	29.2	11.4	10.8

Relative migration - Results in %. Average values

Migration time [days]	Oligo-1	Oligo-2	Oligo-3	Oligo-4	Oligo-5	Oligo-6	Oligo-7	Oligo-8
**1**	n.d.	0.3	0.3	0.1	0.1	0.2	0.2	n.d.
**2**	n.d.	0.4	0.2	0.1	0.2	0.2	0.2	n.d.
**3**	n.d.	0.5	0.2	0.2	0.2	0.2	0.2	n.d.
**7**	n.d.	0.5	0.2	0.2	0.2	0.2	0.2	n.d.
**14**	n.d.	0.6	0.2	0.2	0.2	0.3	0.2	n.d.
**22**	n.d.	0.7	0.2	0.2	0.3	0.3	0.3	n.d.
**29**	1.4	0.8	0.6	0.2	0.3	0.3	0.2	n.d.
**35**	n.d.	0.8	0.5	0.3	0.2	0.4	0.3	0.2
**43**	n.d.	0.8	0.5	0.2	0.3	0.3	0.4	n.d.
**49**	n.d.	0.8	0.6	0.2	0.2	0.4	0.4	n.d.
**56**	n.d.	0.8	0.2	0.3	0.3	0.4	0.4	0.5
**63**	n.d.	0.9	0.2	0.2	0.2	0.3	0.3	n.d.
**70**	n.d.	0.9	0.3	0.3	0.3	0.3	0.4	n.d.
**77**	n.d.	0.9	0.3	0.3	0.3	0.3	0.4	n.d.
**84**	n.d.	0.9	0.3	0.3	0.3	0.3	0.4	n.d.
**91**	n.d.	1.0	0.3	0.2	0.3	0.4	0.4	n.d.

**98**	n.d.	0.9	0.3	0.3	0.3	0.4	0.4	n.d.
**105**	n.d.	1.0	0.4	0.3	0.4	0.4	0.3	0.4
**115**	n.d.	1.0	0.3	0.3	0.3	0.4	0.4	0.4
**133**		1.7	n.d.	0.3	0.4	0.4	0.4	1.1

Weight increase - Results in %. Average values and standard deviation (N=3)

Time contact [days]	Weight increase [%]	SD
**2**	0.76	0.01
**5**	1.23	0.21
**12**	1.71	0.21
**16**	1.89	0.02
**30**	2.19	0.21
**44**	2.32	0.21
**64**	2.48	0.02
**92**	2.48	0.06
**126**	2.57	1.03
**159**	2.60	1.87

17 Migration and weight increase in 50% ethanol at 60 °C

Migration Kinetics - Results in µg/dm². Average values and standard deviation (N=3)

Migration time [days]		Toluene	Chloro-benzene	Styrene	Phenyl-cyclo-hexane	Benzo-phenone	Methyl-stearate
**1**	average	43.9	41.4	20.2	10.6	16.2	n.d.
	SD	16.0	13.1	08.1	01.5	06.7	n.d.
**2**	average	72.7	57.1	30.3	11.6	16.2	n.d.
	SD	19.2	25.6	03.8	01.1	02.6	n.d.
**3**	average	91.4	86.4	34.8	12.1	19.7	n.d.
	SD	21.8	30.4	10.1	02.3	02.3	n.d.
**7**	average	57.8	122.9	49.3	12.5	21.7	n.d.
	SD	60.7	62.4	21.2	02.9	04.4	n.d.
**14**	average	201.3	212.5	64.4	15.2	25.8	n.d.
	SD	51.4	64.7	46.6	01.8	04.3	n.d.
**22**	average	302.3	240.4	90.6	15.4	31.2	n.d.
	SD	91.8	113.3	33.4	03.3	02.5	n.d.
**29**	average	161.4	184.9	79.5	15.6	34.4	n.d.
	SD	22.4	31.9	08.6	01.5	07.6	n.d.
**35**	average	276.1	296.7	116.9	18.1	34.3	n.d.
	SD	n.d.	n.d.	47.0	03.2	03.9	n.d.
**43**	average	457.0	436.7	167.4	21.9	17.3	n.d.
	SD	n.d.	n.d.	16.5	02.2	06.7	n.d.
**49**	average	321.1	302.3	122.1	17.3	8.8	n.d.
	SD	n.d.	n.d.	76.8	07.4	05.4	n.d.
**56**	average	450.8	431.9	185.8	23.9	27.5	n.d.
	SD	n.d.	n.d.	22.8	02.8	06.3	n.d.
**63**	average	301.1	377.4	153.7	20.7	24.3	n.d.
	SD	n.d.	n.d.	48.0	05.0	04.1	n.d.
**70**	average	339.8	323.6	132.5	21.4	43.8	n.d.
	SD	n.d.	n.d.	49.7	04.8	04.1	n.d.
**77**	average	202.5	263.9	123.5	21.0	44.2	n.d.
	SD	n.d.	n.d.	44.3	05.2	06.3	n.d.
**84**	average	230.0	215.9	106.1	21.2	45.5	n.d.
	SD	n.d.	n.d.	19.0	04.3	08.5	n.d.
**91**	average	266.2	206.1	109.7	21.9	55.0	n.d.
	SD	n.d.	n.d.	24.8	02.8	24.5	n.d.
**98**	average	288.6	139.9	87.7	15.2	52.3	n.d.
	SD	n.d.	n.d.	62.7	15.1	13.7	n.d.
**105**	average	161.4	121.7	86.9	20.8	58.9	n.d.
	SD	n.d.	n.d.	54.7	06.4	17.9	n.d.
**115**	average	271.2	285.9	167.8	36.1	77.4	n.d.
	SD	n.d.	n.d.	95.4	15.2	19.1	n.d.

Note: Due to analytical interferences. migration of methyl stearate from HIPS in 50% ethanol at 60 °C can not be measured.

Relative migration - Results in %. Average values

Migration time [days]	Toluene	Chloro-benzene	Styrene	Phenyl-cyclo-hexane	Benzo-phenone	Methyl-stearate
**1**	5.8	5.0	5.6	0.9	1.6	
**2**	9.5	6.9	8.4	0.9	1.6	
**3**	12.0	10.5	9.6	1.0	2.0	
**7**	7.6	15.0	13.6	1.0	2.2	
**14**	26.4	25.9	17.8	1.2	2.6	
**22**	39.6	29.3	25.0	1.2	3.1	
**29**	21.2	22.5	21.9	1.3	3.4	
**35**	36.2	36.1	32.2	1.5	3.4	
**43**	59.9	53.2	46.1	1.8	1.7	
**49**	42.1	36.8	33.7	1.4	0.9	
**56**	59.1	52.6	51.2	1.9	2.7	
**63**	39.5	46.0	42.4	1.7	2.4	
**70**	44.5	39.4	36.5	1.7	4.4	
**77**	26.5	32.1	34.0	1.7	4.4	
**84**	30.1	26.3	29.2	1.7	4.5	
**91**	34.9	25.1	30.2	1.8	5.5	
**98**	37.8	17.0	24.2	1.2	5.2	
**105**	21.2	14.8	24.0	1.7	5.9	
**115**	35.5	34.8	46.3	2.9	7.7	

Migration Kinetics - Results in µg/dm². Average values

Migration time [days]	Oligo-1	Oligo-2	Oligo-3	Oligo-4	Oligo-5	Oligo-6	Oligo-7	Oligo-8
**1**	16.6	12.3	21.5	45.3	12.8	n.d.	n.d.	n.d.
**2**	20.5	15.3	26.3	55.7	16.1	05.1	n.d.	n.d.
**3**	21.3	15.8	23.3	53.8	25.8	06.1	n.d.	n.d.
**7**	24.0	18.4	07.8	39.0	61.1	05.9	n.d.	n.d.
**14**	22.4	15.9	29.4	62.2	18.4	05.3	n.d.	n.d.
**22**	23.9	16.1	31.2	65.6	18.9	06.4	n.d.	n.d.
**29**	25.4	52.2	35.0	74.6	21.7	06.4	n.d.	n.d.
**35**	24.8	45.7	35.2	67.7	19.9	08.9	n.d.	02.1
**43**	21.9	30.2	29.5	60.5	16.8	06.2	n.d.	n.d.
**49**	20.0	12.2	22.1	38.8	10.6	03.3	n.d.	n.d.
**56**	26.6	19.2	31.8	67.7	19.7	08.4	n.d.	04.6
**63**	28.7	12.9	35.1	73.8	21.7	10.3	n.d.	n.d.
**70**	32.6	20.7	40.6	85.4	25.1	11.0	n.d.	n.d.
**77**	30.7	17.8	37.8	80.5	25.1	06.8	n.d.	n.d.
**84**	33.8	24.7	45.3	96.0	27.5	13.4	n.d.	n.d.
**91**	41.4	20.7	42.8	90.2	25.8	09.1	n.d.	n.d.

**98**	34.8	26.8	46.0	94.0	26.4	06.6	n.d.	n.d.
**105**	39.0	23.7	49.0	103.1	28.6	09.2	n.d.	03.9
**115**	42.2	28.9	51.7	108.8	30.7	13.0	06.7	04.0

Relative migration - Results in %. Average values

**Migration time [days]**	**Oligo-1**	**Oligo-2**	**Oligo-3**	**Oligo-4**	**Oligo-5**	**Oligo-6**	**Oligo-7**	**Oligo-8**
**1**	6.3	0.4	0.3	0.3	0.0			
**2**	7.7	0.5	0.3	0.4	0.1	0.1		
**3**	8.0	0.5	0.3	0.4	0.1	0.1		
**7**	9.0	0.6	0.1	0.3	0.2	0.1		
**14**	8.4	0.5	0.3	0.5	0.1	0.1		
**22**	9.0	0.5	0.4	0.5	0.1	0.1		
**29**	9.6	1.6	0.4	0.6	0.1	0.1		
**35**	9.3	1.4	0.4	0.5	0.1	0.1		
**43**	8.3	0.9	0.3	0.5	0.1	0.1		
**49**	7.5	0.4	0.3	0.3	0.0	0.0		
**56**	10.0	0.6	0.4	0.5	0.1	0.1		
**63**	10.8	0.4	0.4	0.6	0.1	0.1		
**70**	12.3	0.7	0.5	0.6	0.1	0.2		
**77**	11.6	0.6	0.4	0.6	0.1	0.1		
**84**	12.7	0.8	0.5	0.7	0.1	0.2		
**91**	15.6	0.6	0.5	0.7	0.1	0.1		
**98**	13.1	0.8	0.5	0.7	0.1	0.1		
**105**	14.7	0.7	0.6	0.8	0.1	0.1		
**115**	15.9	0.9	0.6	0.8	0.1	0.2		

Weight increase - Results in %. Average values and standard deviation (N=3)

Time contact [days]	Weight increase [%]	SD
**0.2**	0.8	0.01
**0.3**	1.0	0.08
**1**	1.4	0.09
**2**	1.5	0.02
**5**	1.4	0.19
**12**	1.3	0.19
**16**	1.3	0.02
**30**	1.3	0.05
**44**	1.4	0.04
**64**	1.7	0.01
**92**	2.7	0.00
**126**	7.3	0.03
**159**	8.5	0.03
**201**	9.7	0.02
**273**	10.0	0.05

18 Migration and weight increase in 50% ethanol at 40 °C

Migration Kinetics - Results in µg/dm². Average values and standard deviation (N=3)

Migration time [days]		Toluene	Chloro-benzene	Styrene	Phenyl-cyclo-hexane	Benzo-phenone	Methyl-stearate
**1**	average	43.0	42.0	16.0	13.0	17.0	n.d.
	SD	03.5	03.3	01.6	00.7	03.7	n.d.
**3**	average	59.0	66.0	24.0	14.0	23.0	n.d.
	SD	09.3	05.2	01.4	05.8	03.2	n.d.
**7**	average	92.0	121.0	39.0	21.0	28.0	n.d.
	SD	04.2	15.6	04.8	01.2	01.6	n.d.
**16**	average	156.0	190.0	60.0	23.0	31.0	n.d.
	SD	09.7	12.7	03.8	01.2	02.7	n.d.
**36**	average	213.0	269.0	84.0	26.0	39.0	n.d.
	SD	23.8	31.2	09.3	02.6	04.8	n.d.
**49**	average	260.0	339.0	105.0	27.0	40.0	n.d.
	SD	35.6	39.8	11.6	02.1	01.6	n.d.
**64**	average	309.0	404.0	127.0	30.0	41.0	n.d.
	SD	32.4	41.7	10.1	04.4	05.7	n.d.

Note: Due to analytical interferences. migration of methylstearate from HIPS in 50% ethanol at 40 °C can not be measured.

Relative migration - Results in %. Average values

Migration time [days]	Toluene	Chloro-benzene	Styrene	Phenyl-cyclo-hexane	Benzo-phenone	Methyl-stearate
**1**	2.8	2.6	2.2	0.5	0.9	
**3**	3.9	4.1	3.3	0.6	1.2	
**7**	6.1	7.4	5.4	0.9	1.4	
**16**	10.3	11.7	8.4	0.9	1.6	
**36**	14.1	16.5	11.7	1.1	2.0	
**49**	17.2	20.8	14.6	1.1	2.0	
**64**	20.5	24.8	17.7	1.2	2.1	

Migration Kinetics - Results in µg/dm². Average values

Migration time [days]	Oligo-1	Oligo-2	Oligo-3	Oligo-4	Oligo-5	Oligo-6	Oligo-7	Oligo-8
1	n.d.	10.07	7.45	12.82	29.26	13.59	3.54	n.d.
3	n.d.	12.86	9.39	19.48	39.76	11.70	4.16	n.d.
7	n.d.	15.39	9.90	21.27	43.39	13.26	3.90	n.d.
16	n.d.	17.19	11.13	23.20	47.89	14.23	3.78	n.d.
36	n.d.	19.28	11.83	21.72	46.43	20.90	4.68	n.d.
49	n.d.	20.21	12.56	26.07	53.64	15.14	5.02	n.d.
64	n.d.	17.78	14.19	26.80	54.65	17.87	5.26	n.d.

Relative migration - Results in %. Average values

Migration time [days]	Oligo-1	Oligo-2	Oligo-3	Oligo-4	Oligo-5	Oligo-6	Oligo-7	Oligo-8
1	n.d.	0.3	0.1	0.1	0.1	0.2	0.1	n.d.
3	n.d.	0.4	0.1	0.1	0.1	0.2	0.1	n.d.
7	n.d.	0.5	0.1	0.2	0.2	0.2	0.1	n.d.
16	n.d.	0.5	0.1	0.2	0.2	0.2	0.1	n.d.
36	n.d.	0.6	0.1	0.2	0.2	0.3	0.1	n.d.
49	n.d.	0.6	0.1	0.2	0.2	0.2	0.2	n.d.
64	n.d.	0.6	0.2	0.2	0.2	0.2	0.2	n.d.

Weight increase - Results in %. Average values and standard deviation (N=3)

Time contact [days]	Weight increase [%]	SD
1	0.9	0.02
2	1.0	0.09
5	1.3	0.08
12	1.3	0.03
16	1.3	0.12
30	1.3	0.11
44	1.3	0.01
64	1.3	0.04
92	1.3	0.03
126	1.3	0.02
159	1.3	0.04

19 Migration and weight increase in 50% ethanol at 20 °C

Migration Kinetics - Results in µg/dm². Average values and standard deviation (N=3)

Migration time [days]		Toluene	Chloro-benzene	Styrene	Phenyl-cyclo-hexane	Benzo-phenone	Methyl-stearate
**1**	average	19	12	2	5	6	n.d.
	SD	10.9	05.5	00.0	00.6	00.0	n.d.
**3**	average	21	20	11	7	6	n.d.
	SD	13.9	04.3	01.3	00.8	00.0	n.d.
**7**	average	30	41	15	11	13	n.d.
	SD	16.6	09.4	02.2	02.4	03.1	n.d.
**16**	average	25	48	17	13	17	n.d.
	SD	16.4	11.2	03.8	01.5	03.1	n.d.
**36**	average	68	84	28	17	21	n.d.
	SD	14.6	15.8	03.0	01.1	02.8	n.d.
**49**	average	59	84	26	16	21	n.d.
	SD	22.7	18.9	04.7	02.3	03.2	n.d.
**64**	average	89	125	33	18	24	n.d.
	SD	69.9	47.0	59.9	02.8	03.1	n.d.

Note: Due to analytical interferences. migration of methyl stearate from HIPS in 50% ethanol at 20 °C can not be measured.

Relative migration - Results in %. Average values

Migration time [days]	Toluene	Chloro-benzene	Styrene	Phenyl-cyclo-hexane	Benzo-phenone	Methyl-stearate
**1**	1.3	0.7	0.3	0.2	0.3	
**3**	1.4	1.2	1.5	0.3	0.3	
**7**	2.0	2.5	2.1	0.5	0.7	
**16**	1.7	3.0	2.4	0.5	0.9	
**36**	4.5	5.2	3.9	0.7	1.1	
**49**	3.9	5.2	3.6	0.7	1.1	
**64**	5.9	7.7	4.6	0.8	1.2	

Migration Kinetics - Results in µg/dm². Average values

Migration time [days]	Oligo-1	Oligo-2	Oligo-3	Oligo-4	Oligo-5	Oligo-6	Oligo-7	Oligo-8
**1**	n.d.	02.2	n.d.	06.6	14.0	07.0	n.d.	n.d.
**3**	n.d.	03.2	11.7	11.2	23.2	10.8	n.d.	n.d.
**7**	n.d.	07.2	06.1	14.1	28.1	08.2	n.d.	n.d.
**16**	n.d.	08.5	06.3	13.8	30.6	13.6	02.9	n.d.
**36**	n.d.	06.7	06.9	12.0	27.8	16.6	02.8	n.d.
**49**	n.d.	10.6	08.0	13.7	31.0	19.4	03.3	n.d.
**64**	n.d.	11.8	07.7	18.1	35.4	09.5	03.6	n.d.

Relative migration - Results in %. Average values

Migration time [days]	Oligo-1	Oligo-2	Oligo-3	Oligo-4	Oligo-5	Oligo-6	Oligo-7	Oligo-8
**1**	n.d.	0.1	n.d.	0.0	0.1	0.1	n.d.	n.d.
**3**	n.d.	0.1	0.1	0.1	0.1	0.1	n.d.	n.d.
**7**	n.d.	0.2	0.1	0.1	0.1	0.1	n.d.	n.d.
**16**	n.d.	0.3	0.1	0.1	0.1	0.2	0.1	n.d.
**36**	n.d.	0.2	0.1	0.1	0.1	0.2	0.1	n.d.
**49**	n.d.	0.3	0.1	0.1	0.1	0.3	0.1	n.d.
**64**	n.d.	0.4	0.1	0.1	0.1	0.1	0.1	n.d.

Weight increase - Results in %. Average values and standard deviation (N=3)

Time contact [days]	Weight increase [%]	SD
**2**	0.5	0.03
**5**	0.7	0.14
**12**	0.9	0.12
**16**	1.0	0.03
**30**	1.1	0.13
**44**	1.1	0.11
**64**	1.2	0.04
**92**	1.2	0.06
**126**	1.2	0.05
**159**	1.4	0.04
**201**	1.2	0.08
**273**	1.3	0.06

20 Weight increase in 20% ethanol at 60. 40 and 20 °C

Weight increase at 60 °C - Results in %. Average values and standard deviation (N=3)

Time contact [days]	Weight increase [%]	SD
**2**	0.7	0.004
**5**	0.7	0.003
**12**	0.7	0.003
**16**	0.7	0.001
**30**	0.7	0.010
**44**	0.8	0.009
**64**	0.8	0.007
**92**	0.9	0.005
**126**	1.6	0.004
**159**	2.6	0.011
**201**	3.8	0.014
**273**	5.2	0.015

Weight increase at 40 °C - Results in %. Average values and standard deviation (N=3)

Time contact [days]	Weight increase [%]	SD
**2**	0.5	0.022
**5**	0.6	0.056
**12**	0.6	0.053
**16**	0.6	0.007
**30**	0.6	0.007
**44**	0.6	0.009
**64**	0.6	0.011
**92**	0.6	0.020
**126**	0.6	0.023
**159**	0.6	0.023

Weight increase at 20 °C - Results in %. Average values and standard deviation (N=3)

Time contact [days]	Weight increase [%]	SD
**2**	0.2	0.009
**5**	0.4	0.071
**12**	0.4	0.060
**16**	0.4	0.026
**30**	0.5	0.037
**44**	0.5	0.046
**64**	0.5	0.014

**92**	0.5	0.020
**126**	0.5	0.021
**159**	0.5	0.007

21 Weight increase in 10% ethanol at 60. 40 and 20 °C

Weight increase at 60 °C - Results in %. Average values and standard deviation (N=3)

Time contact [days]	Weight increase [%]	SD
**2**	0.4	0.01
**5**	0.4	0.02
**12**	0.4	0.02
**16**	0.4	0.02
**30**	0.4	0.01
**44**	0.5	0.01
**64**	0.5	0.00
**92**	0.7	0.01
**126**	1.1	0.02
**159**	1.7	0.01
**201**	2.3	0.01
**273**	3.3	0.00

Weight increase at 40 °C - Results in %. Average values and standard deviation (N=3)

Time contact [days]	Weight increase [%]	SD
**2**	0.2	0.010
**5**	0.3	0.047
**12**	0.3	0.048
**16**	0.3	0.006
**30**	0.3	0.005
**44**	0.3	0.004
**64**	0.3	0.008
**92**	0.3	0.011
**126**	0.3	0.015
**159**	0.3	0.004

Weight increase at 20 °C - Results in %. Average values and standard deviation (N=3)

Time contact [days]	Weight increase [%]	SD
**2**	0.1	0.005
**5**	0.2	0.028
**12**	0.2	0.027
**16**	0.2	0.005
**30**	0.2	0.022
**44**	0.3	0.018
**64**	0.3	0.003
**92**	0.2	0.006
**126**	0.3	0.006
**159**	0.3	0.010

22 Weight increase in 3% acetic acid at 60. 40 and 20 °C

Weight increase at 60°C - Results in %. Average values and standard deviation (N=3)

Time contact [days]	Weight increase [%]	SD
**2**	0.3	0.010
**5**	0.4	0.129
**12**	0.4	0.114
**16**	0.6	0.081
**30**	0.6	0.037
**44**	0.7	0.036
**64**	0.8	0.019
**92**	0.9	0.042
**126**	0.9	0.116
**159**	1.1	0.085
**201**	1.1	0.072
**273**	1.2	0.051

Weight increase at 40 °C - Results in %. Average values and standard deviation (N=3)

Time contact [days]	Weight increase [%]	SD
**2**	0.1	0.028
**5**	0.2	0.030
**12**	0.2	0.029

**16**	0.3	0.002
**30**	0.3	0.010
**44**	0.3	0.015
**64**	0.3	0.015
**92**	0.3	0.022
**126**	0.3	0.028
**159**	0.4	0.005
**201**	0.4	0.013
**273**	0.6	0.013

Weight increase at 20 °C- Results in %. Average values and standard deviation (N=3)

**Time contact [days]**	**Weight increase [%]**	**SD**
**2**	0.1	0.003
**5**	0.1	0.012
**12**	0.2	0.010
**16**	0.2	0.010
**30**	0.2	0.021
**44**	0.2	0.014
**64**	0.2	0.006
**92**	0.2	0.005
**126**	0.2	0.003
**159**	0.3	0.015
