# Supplementary material for: Migration Testing of GPPS and HIPS Polymers: Swelling Effect Caused by Food Simulants Compared to Real Foods
Source: Molecules. 2022 Jan 26;27(3):823. doi: 10.3390/molecules27030823 (PMC8839621; doi:10.3390/molecules27030823)
Supplement: Supplementary file 1 [file molecules-27-00823-s001.zip › Suppmaterial_2_visualchanges.pdf]

Article

Migration testing of GPPS and HIPS polymers: swelling effect caused by food simulants compared to real foods

Valeria Guazzotti *, Anita Gruner, Mladen Juric, Veronika Hendrich, Angela Störmer and Frank Welle

Fraunhofer Institute for Process Engineering and Packaging, IVV. Giggenhauser Straße 35, 85354 Freising. Germany

*correspondence: valeria.guazzotti@ivv.fraunhofer.de

Supplementary material -2 (Visual changes)

In addition to the analytical determination of the weight increase and of the migration kinetics from GPPS and HIPS polymers after contact with different food and food simulants at several time-temperature testing conditions, at most kinetic time points photos were taken to document visual changes of the sample material.

For a better comparison, an original GPPS or HIPS strip (marked with a black ring) was put next to the test strip in all photos.

GPPS (general purpose polystyrene)

1 Contact with Isooctane

1.1 Isooctane at 60 °C

1 h	2 h	3 h
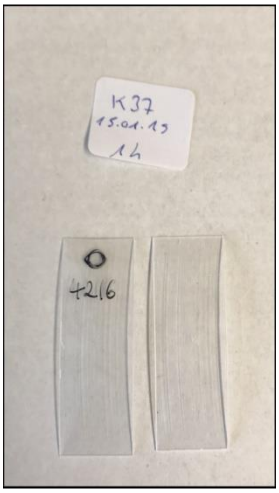	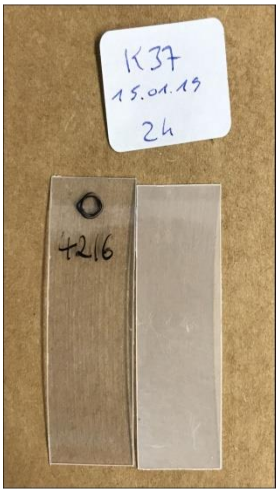	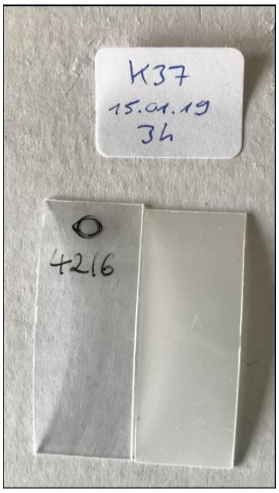
**4 h**	**6 h**	**8 h**
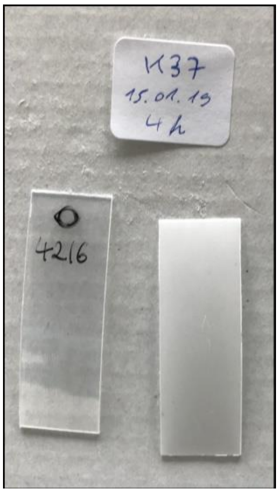	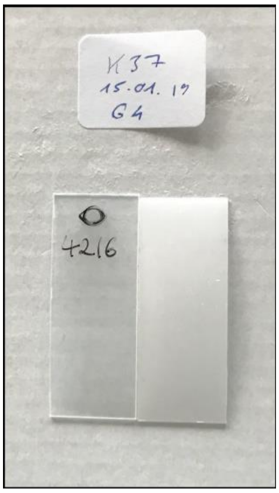	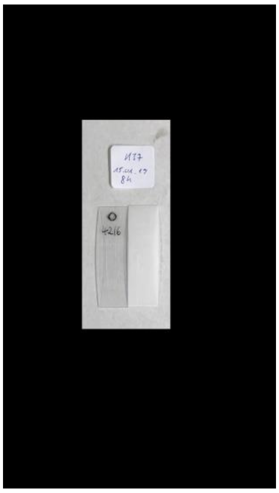

<table>
<tr>
<td>1 d
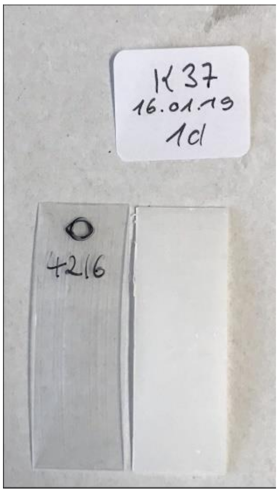
</td>
<td>2 d
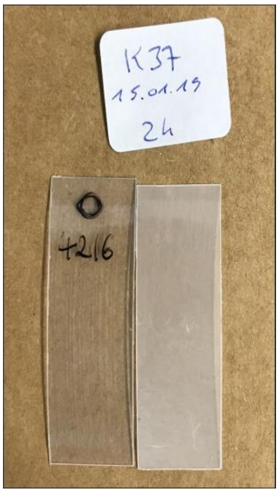
</td>
<td>3 d
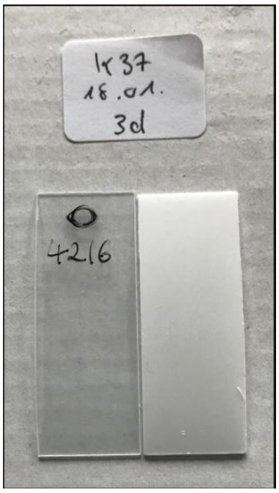
</td>
</tr>
<tr>
<td>6 d
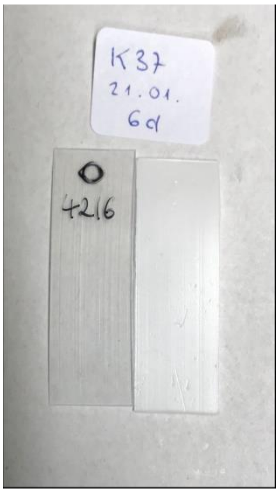
</td>
<td>8 d
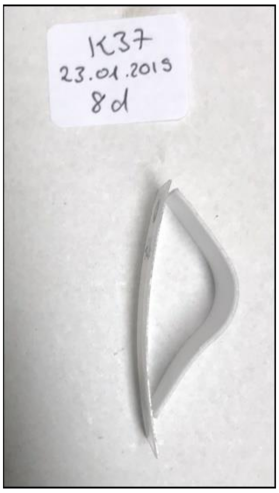
</td>
<td>13 d
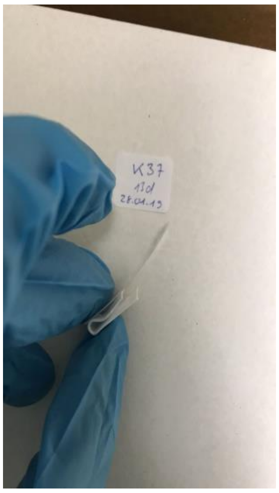</td>
</tr>
<tr>
<td>17 d
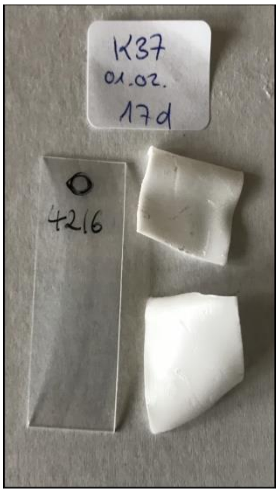
</td>
<td>23 d
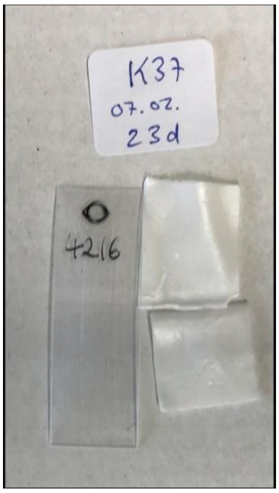
</td>
<td>29 d
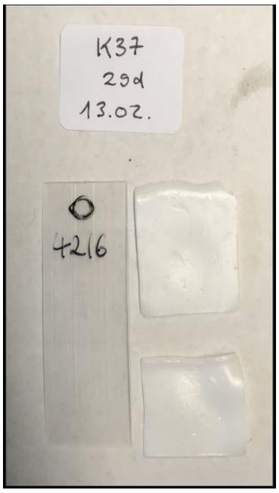
</td>
</tr>
</table>

<table>
<tr>
<td>45 d
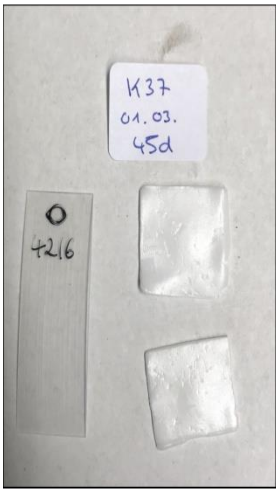
</td>
<td>65 d
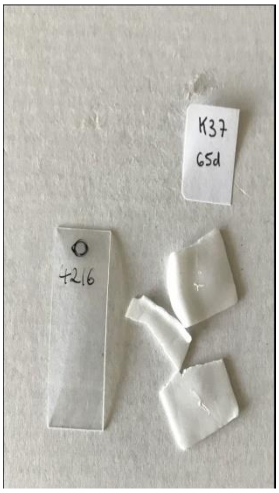
</td>
<td>73 d
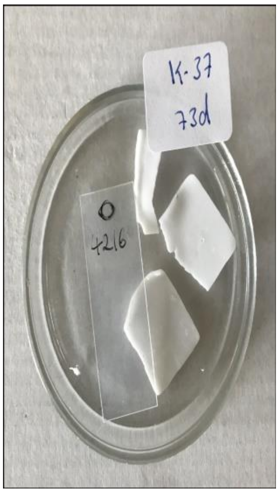
</td>
</tr>
<tr>
<td>99 d
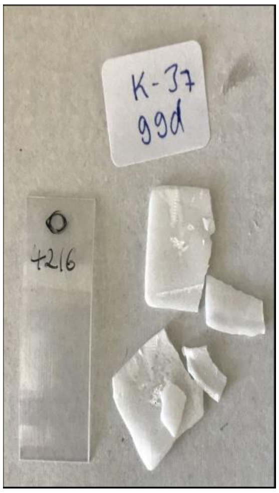
</td>
<td>114 d
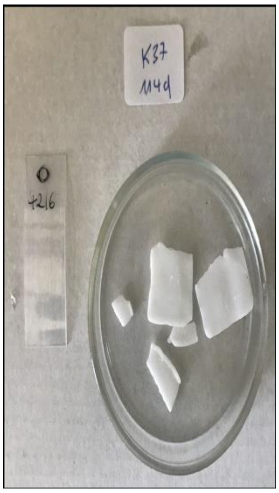
</td>
<td>143 d
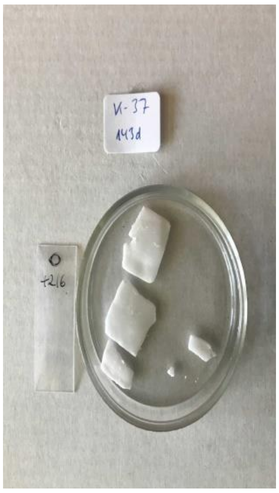
</td>
</tr>
</table>

1.2 Isooctane at 40 °C

4 h

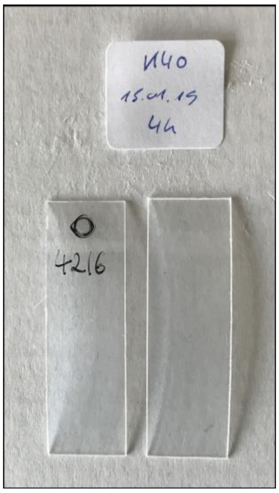

8 h

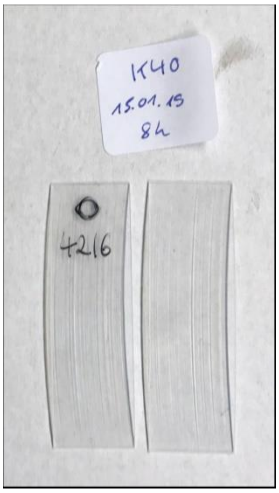

1 d

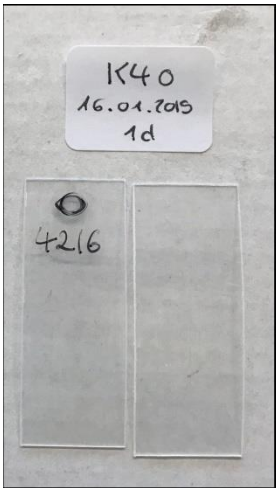

2 d

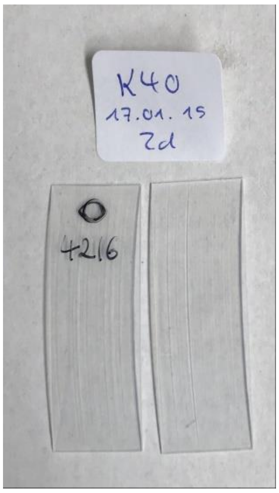

3 d

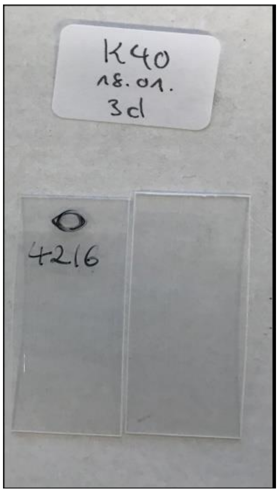

6 d

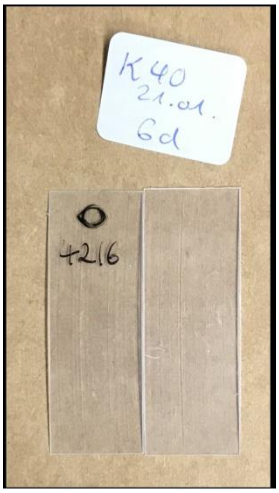

8 d

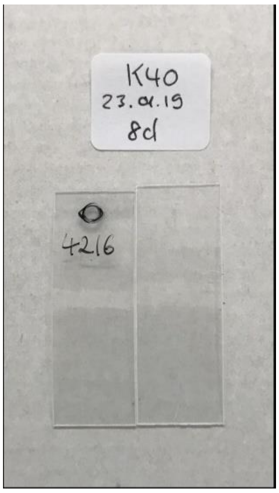

13 d

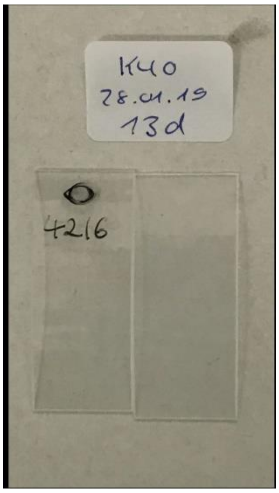

17 d

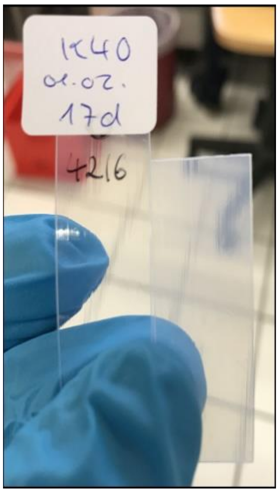

23 d

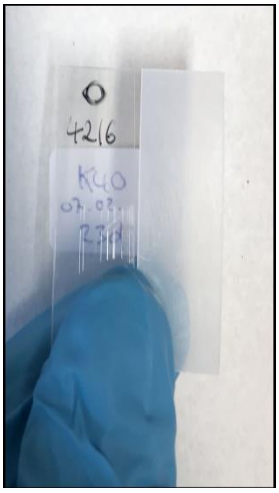

29 d

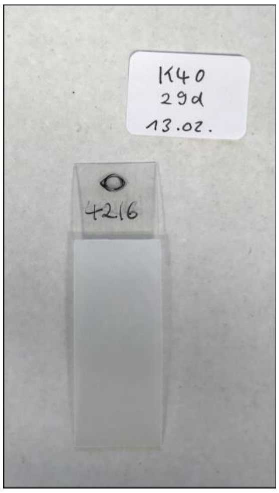

45 d

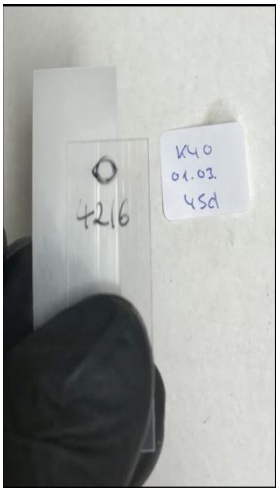

73 d

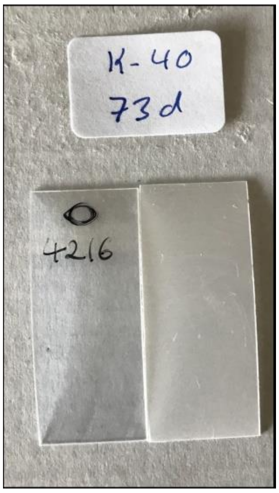

99 d

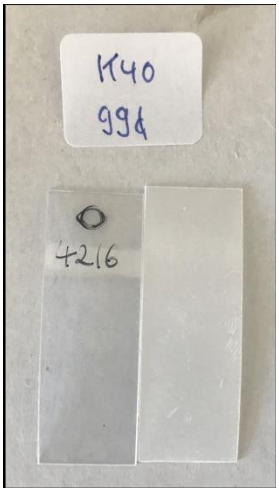

114 d

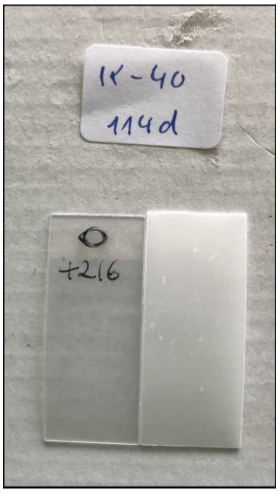

1.3 Isooctane at 20 °C

1 d

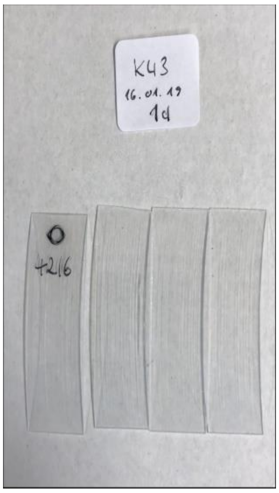

2 d

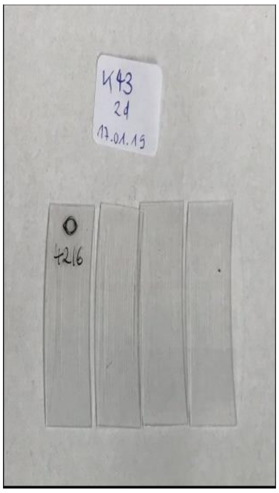

3 d

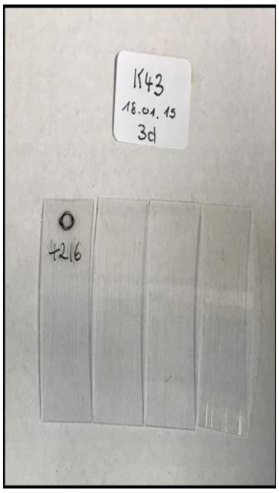

6 d

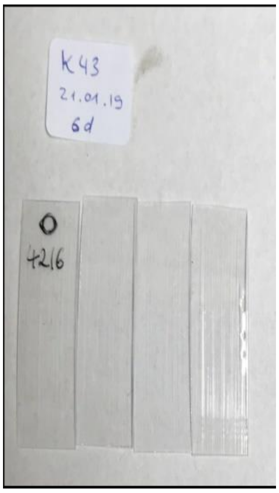

8 d

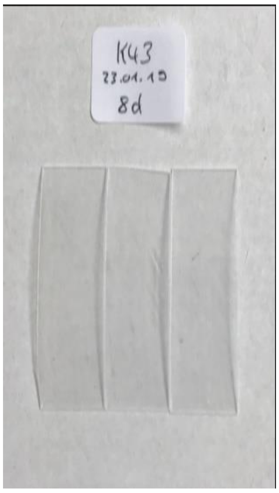

13 d

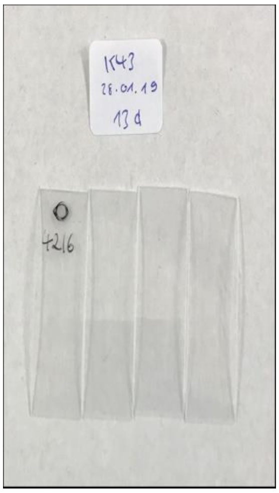

17 d

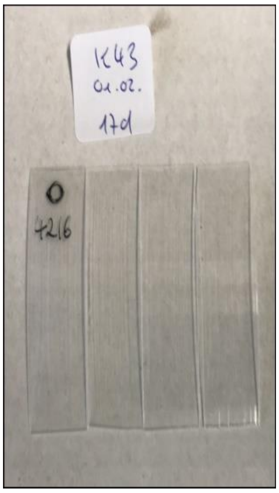

23 d

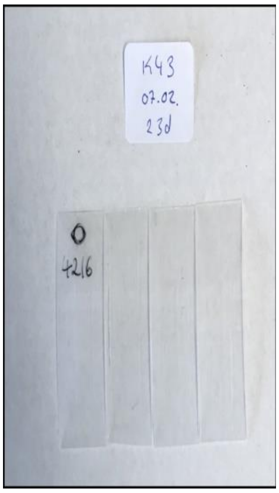

45 d

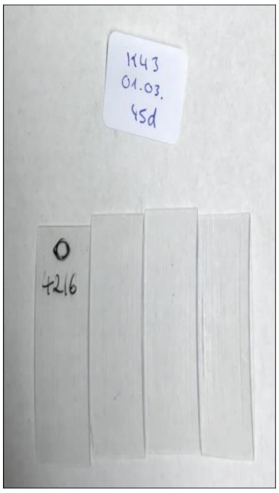

<table>
<tr>
<td>65 d
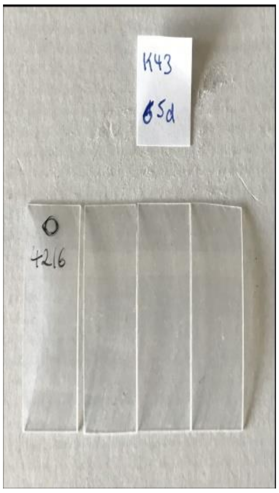
</td>
<td>73 d
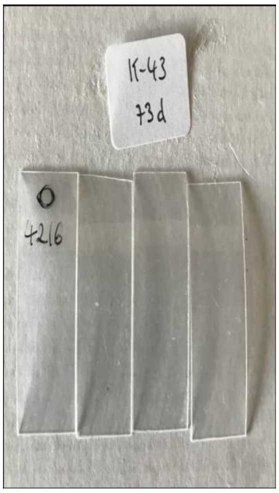
</td>
<td></td>
</tr>
</table>

2 Contact with 95% Ethanol

2.1 95% Ethanol at 60 °C

0.5 h 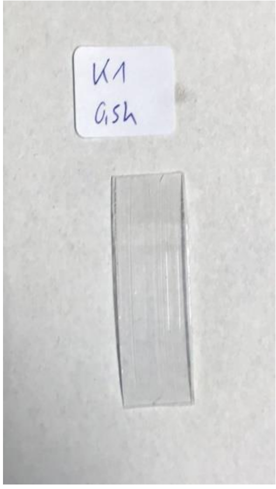	1 h 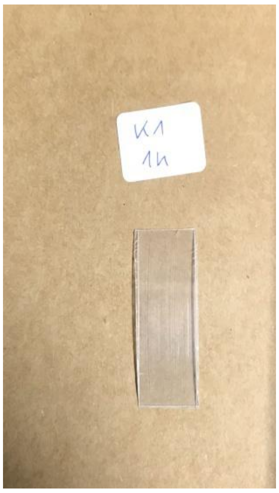	2 h 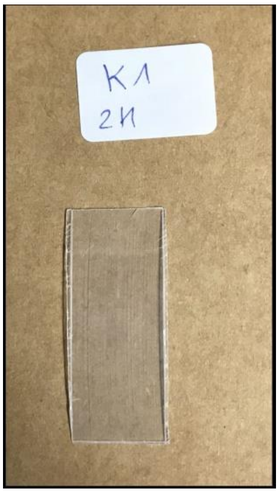
3 h 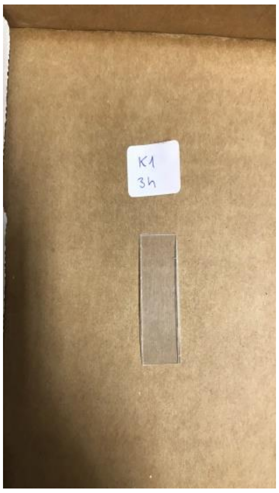	4 h 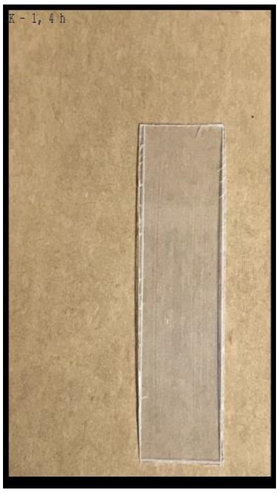	6 h 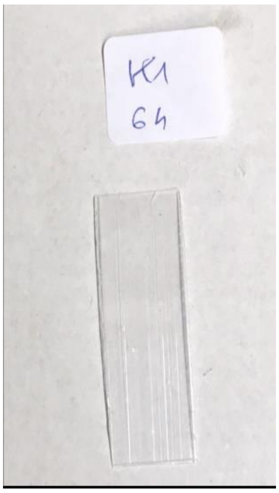

<table>
<tr>
<td>8 h
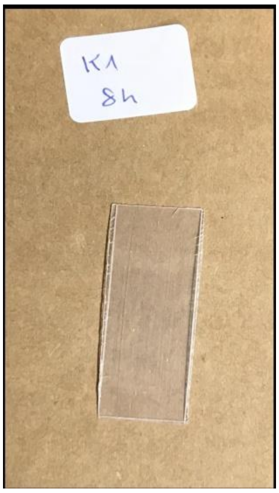</td>
<td>1 d
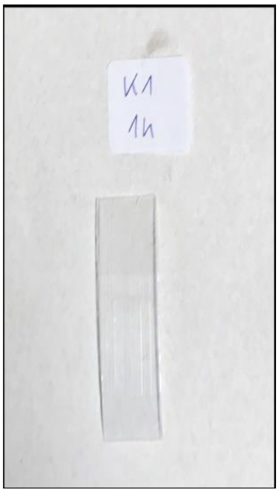</td>
<td>2 d
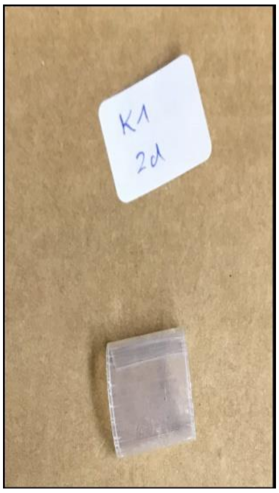</td>
</tr>
<tr>
<td>5 d
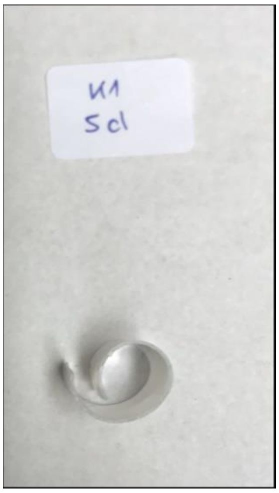</td>
<td>8 d
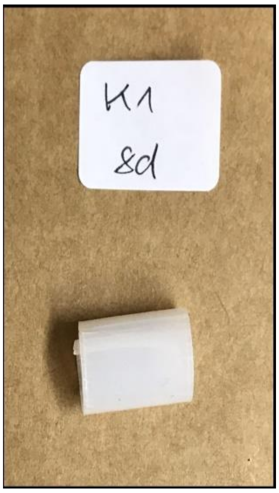</td>
<td>12 d
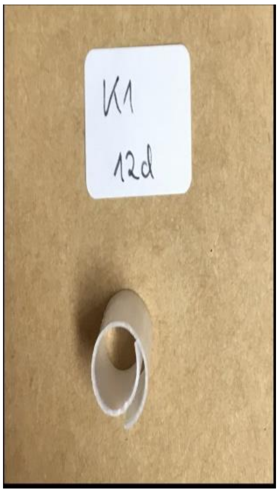</td>
</tr>
<tr>
<td>15 d
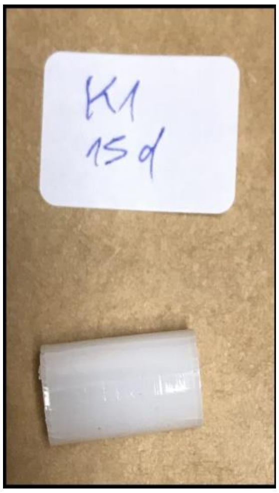</td>
<td>19 d
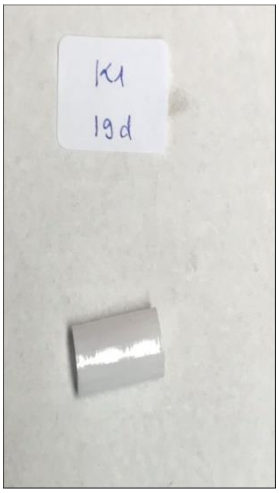</td>
<td>22 d
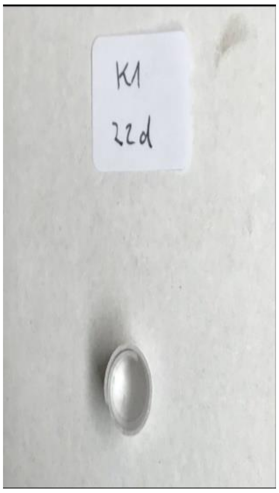</td>
</tr>
</table>

26 d	44 d	57 d
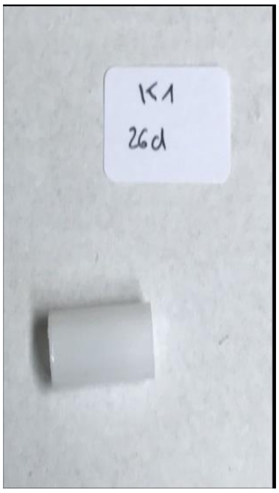	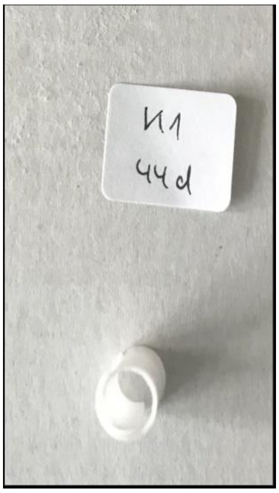	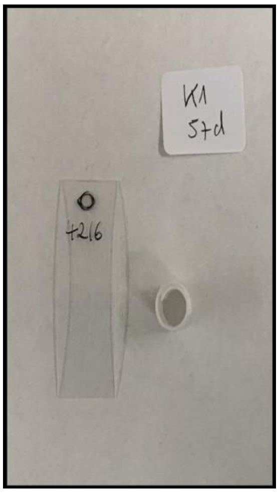
72 d	100 d	127 d
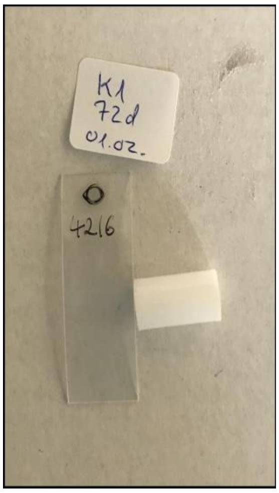	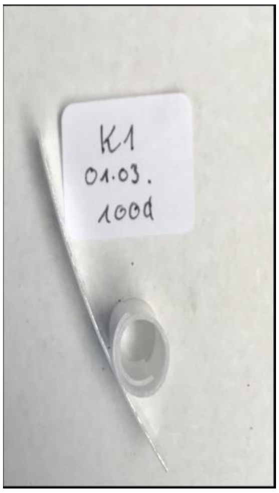	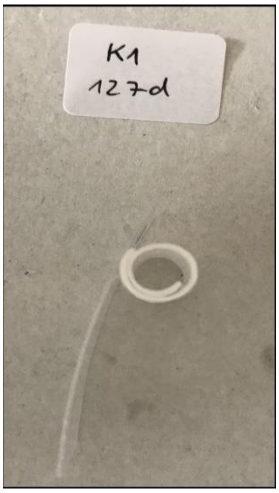
154 d	169 d	198 d
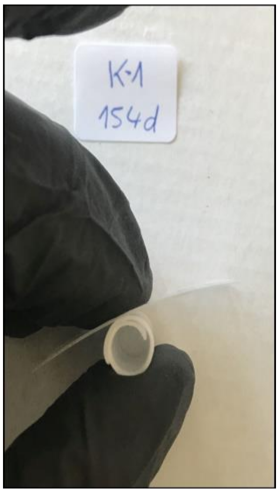	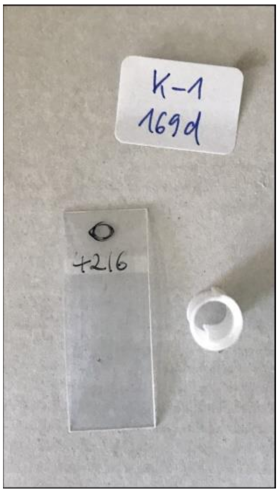	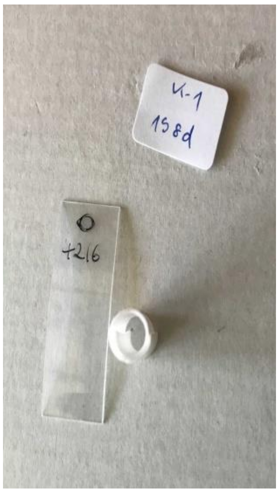

2.2 95% Ethanol at 40 °C

4 h<br>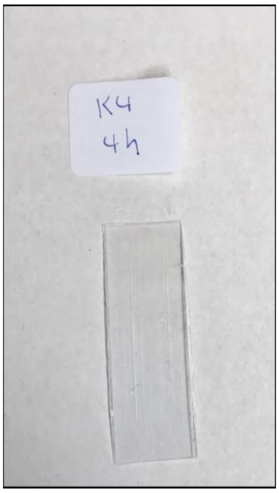	8 h<br>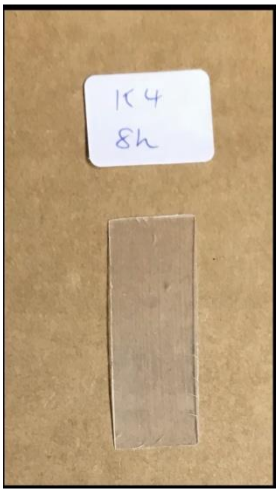	2 d<br>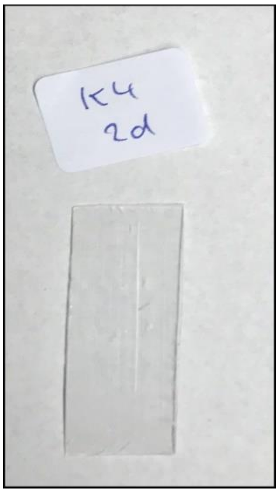
5 d<br>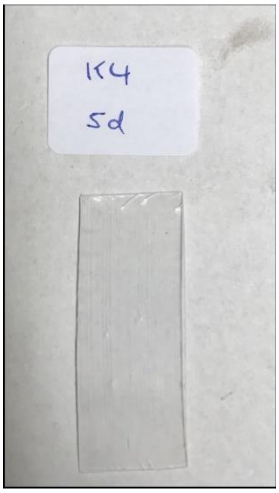	8 d<br>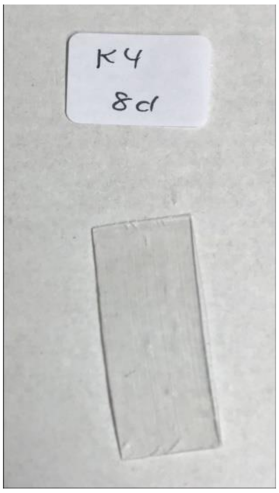	12 d<br>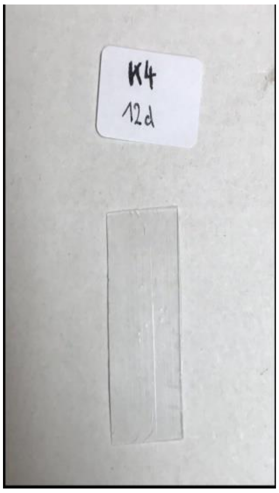
19 d<br>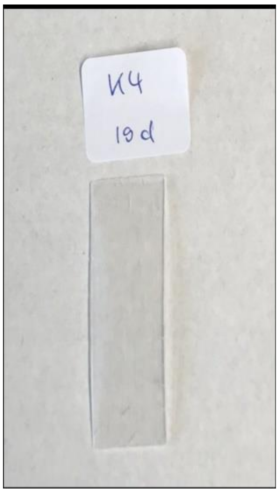	26 d<br>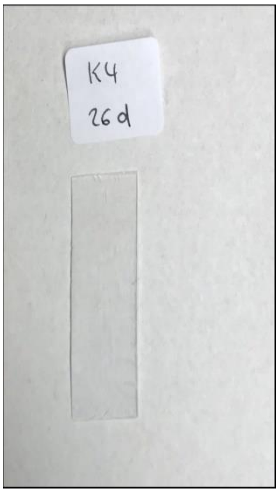	44 d<br>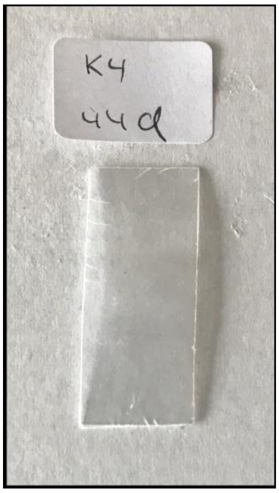

57 d

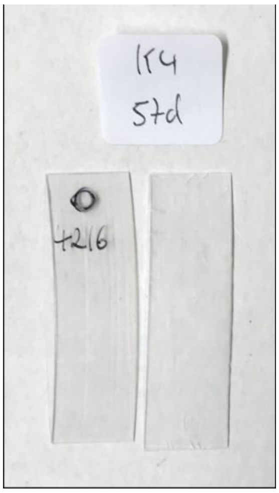

72 d

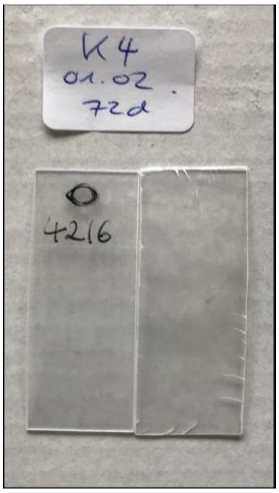

100 d

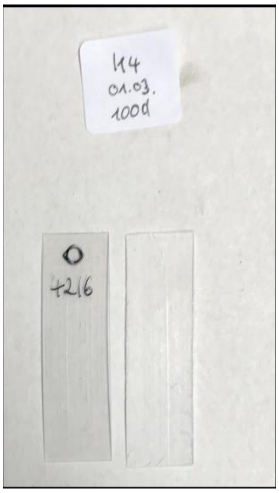

127 d

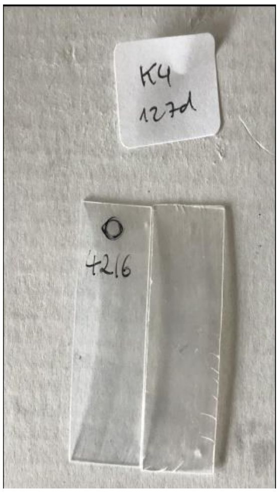

154 d

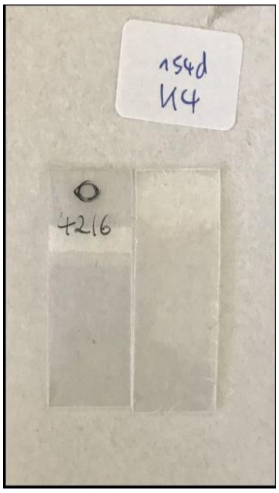

169 d

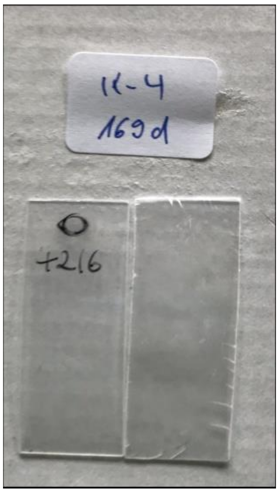

2.3 95% Ethanol at 20 °C

<table>
<tr>
<td>2 d
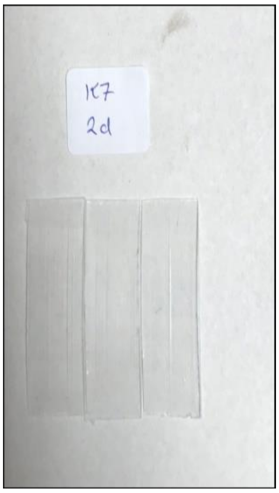</td>
<td>5 d
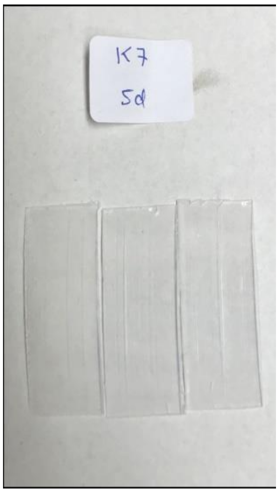</td>
<td>8 d
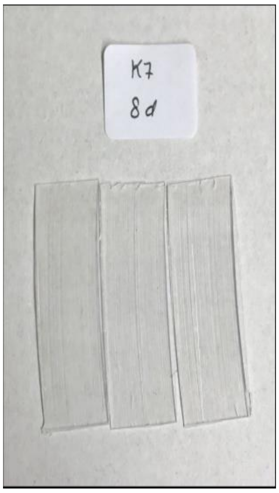</td>
</tr>
<tr>
<td>12 d
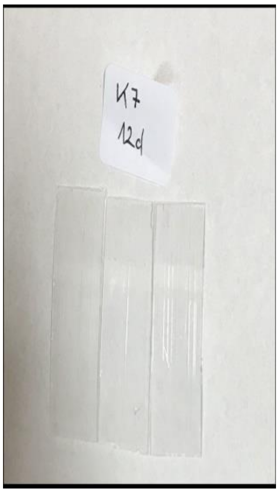</td>
<td>19 d
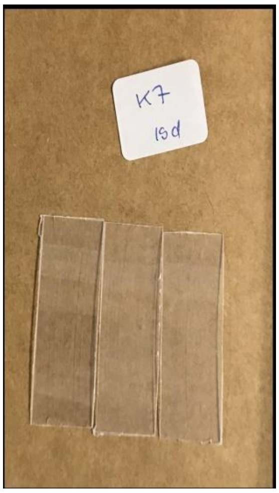</td>
<td>26 d
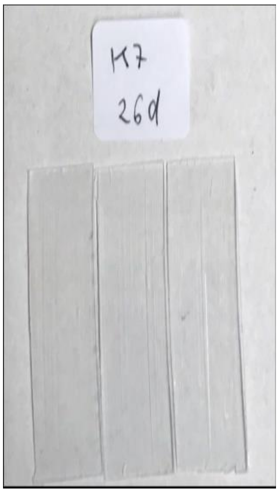</td>
</tr>
<tr>
<td>44 d
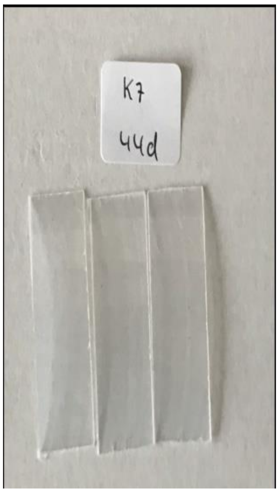</td>
<td>57 d
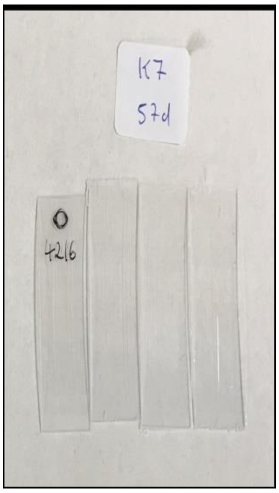</td>
<td>72 d
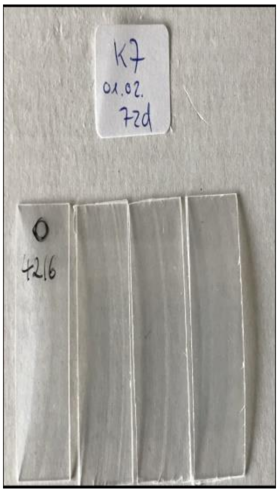</td>
</tr>
</table>

100 d	154 d	198 d
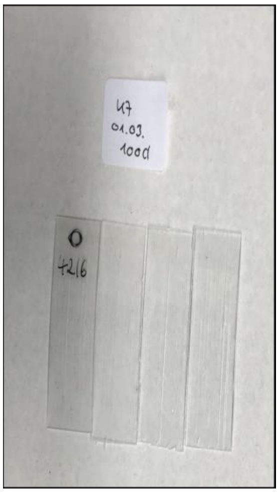	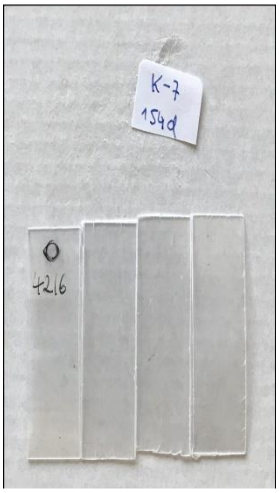	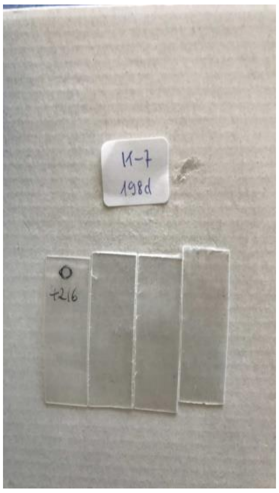

3 Contact with 50% Ethanol

3.1 50% Ethanol at 60 °C

0.5 h 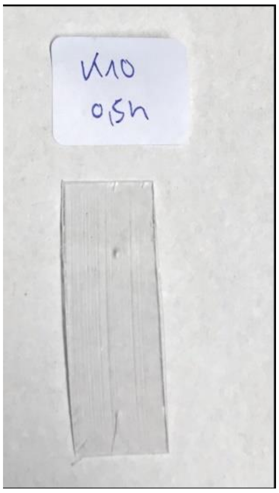	1 h 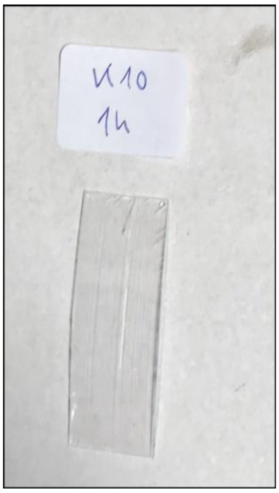	2 h 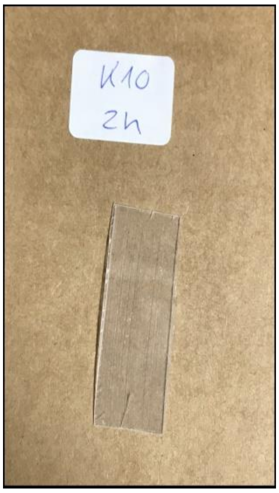
3 h 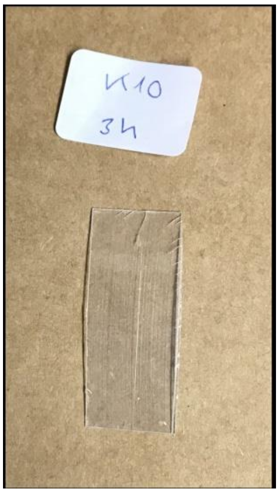	4 h 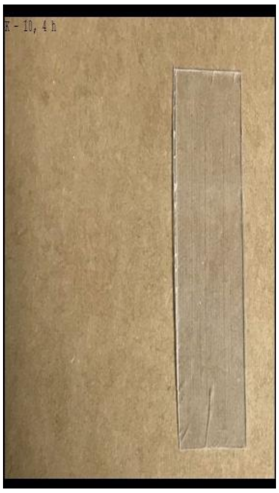	6 h 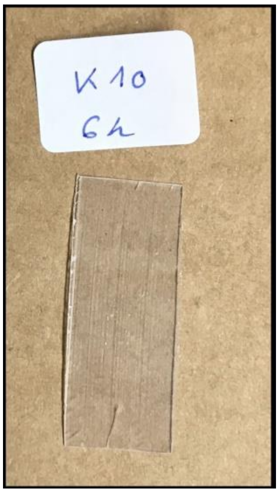

8 h<br>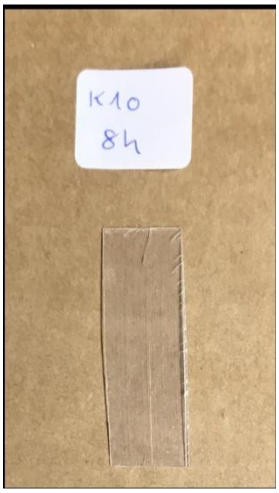	1 d<br>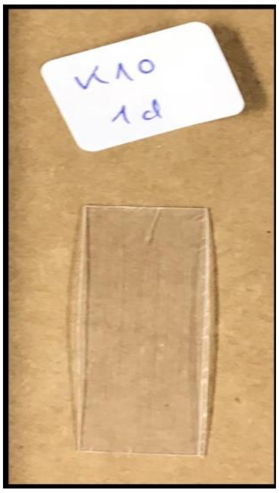	2 d<br>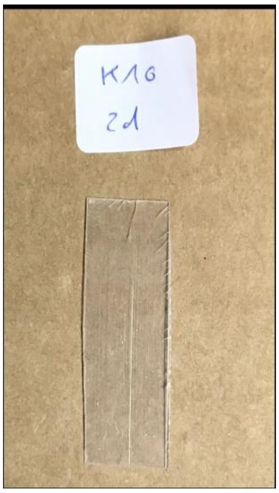
5 d<br>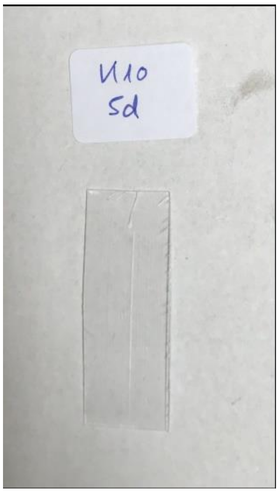	8 d<br>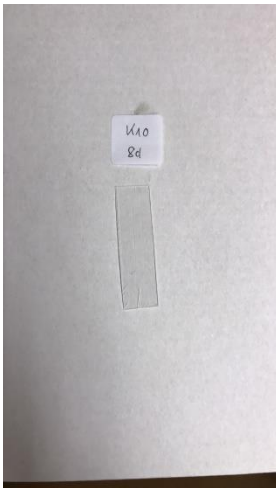	12 d<br>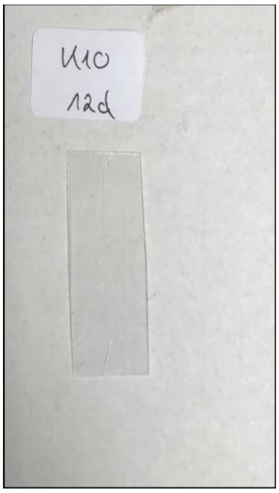
19 d<br>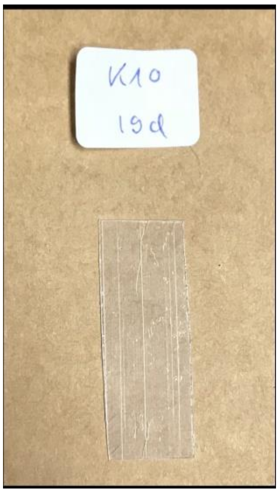	26 d<br>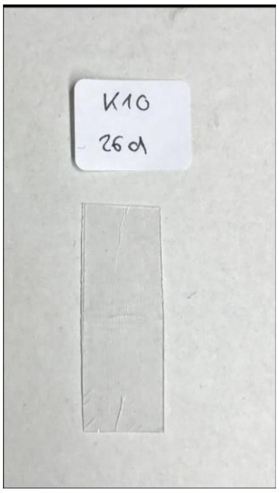	44 d<br>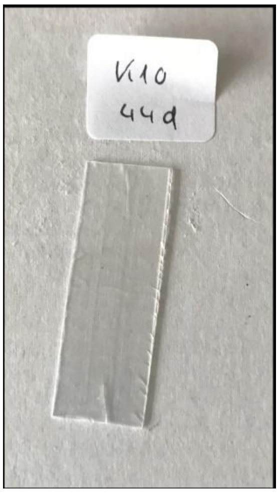

57 d	72 d	127 d
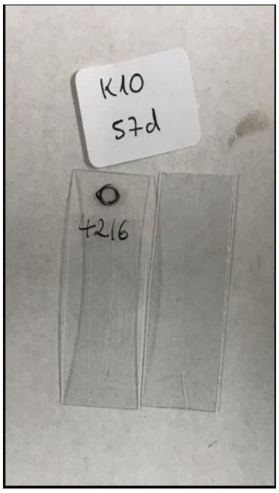 K10<br>57d<br>4216	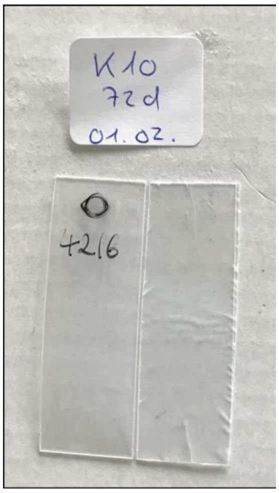 K10<br>72d<br>01.02.<br>4216	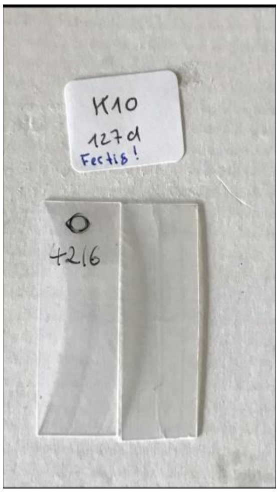 K10<br>127d<br>Fertig!<br>4216

3.2 50% Ethanol at 40 °C

4 h

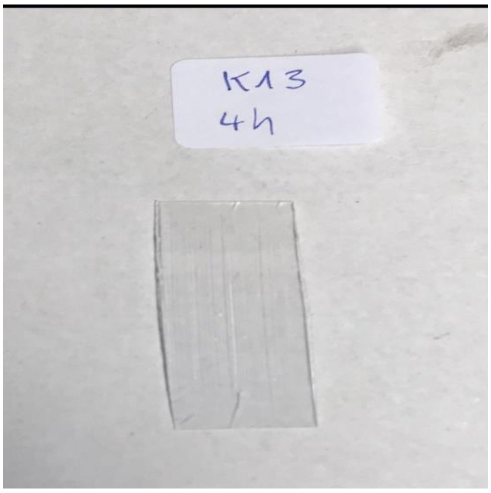

8 h

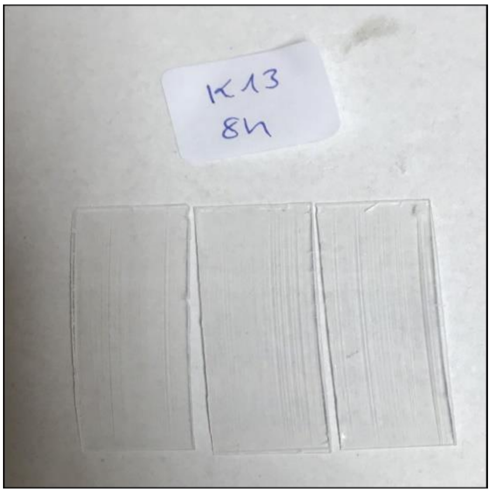

1 d

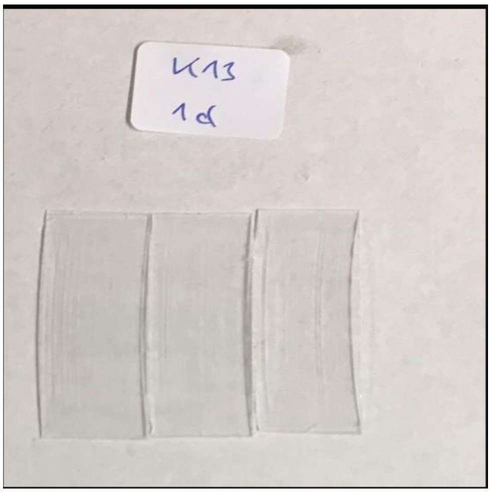

5 d

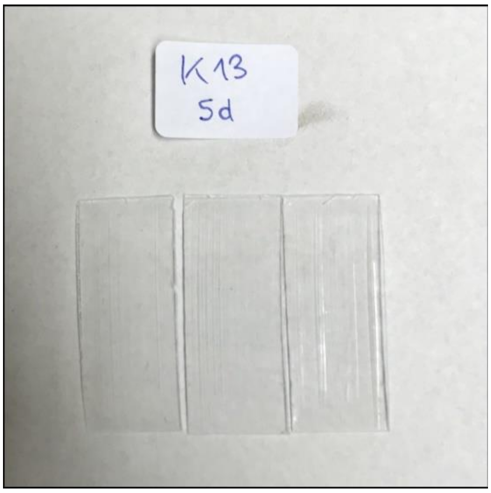

8 d

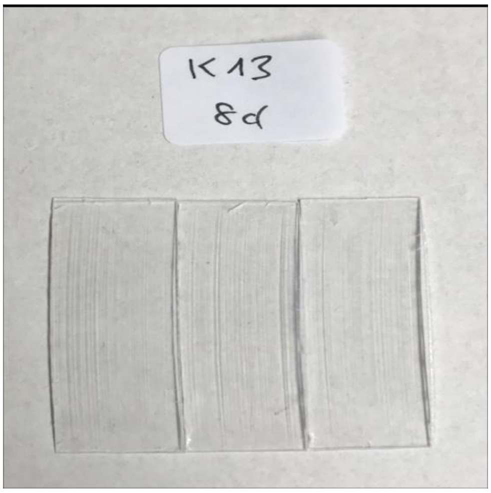

12 d

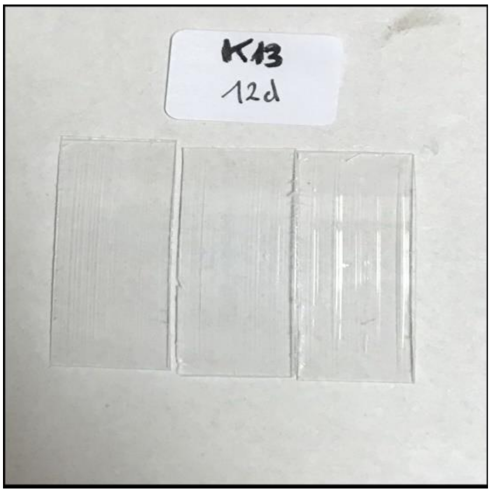

19 d

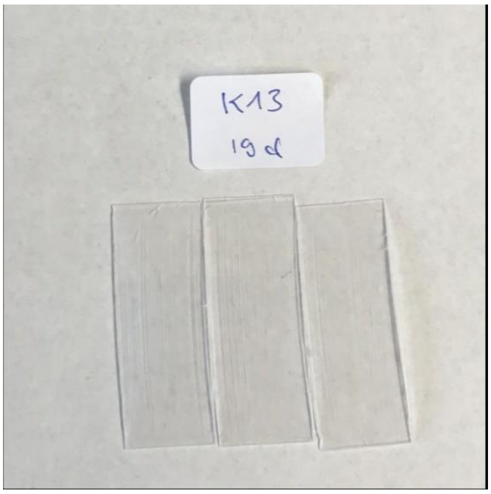

26 d

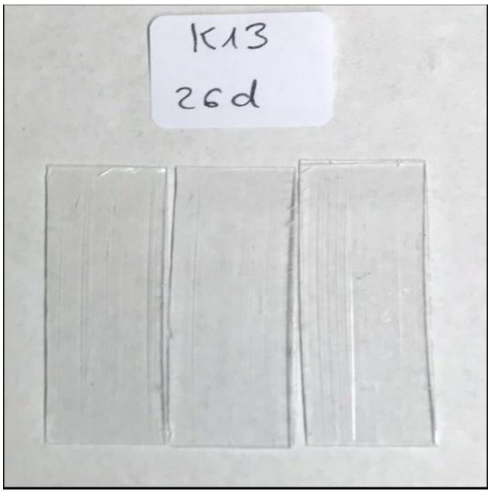

44 d

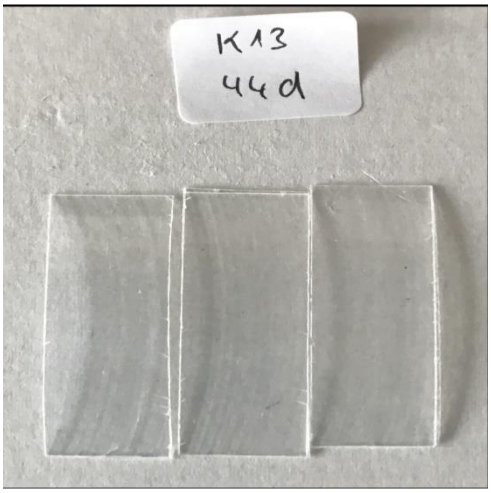

57 d

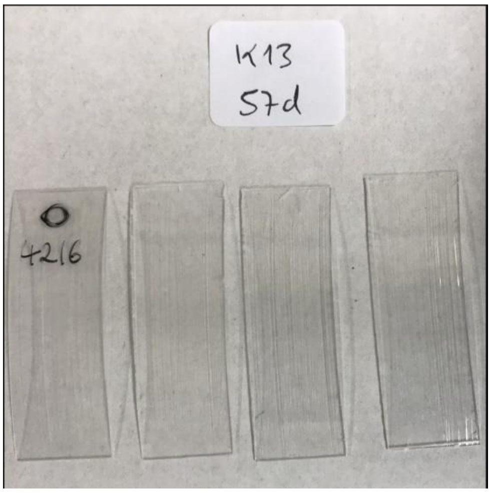

72 d

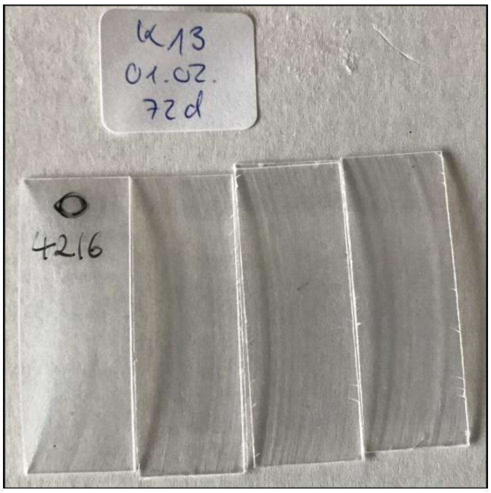

127 d

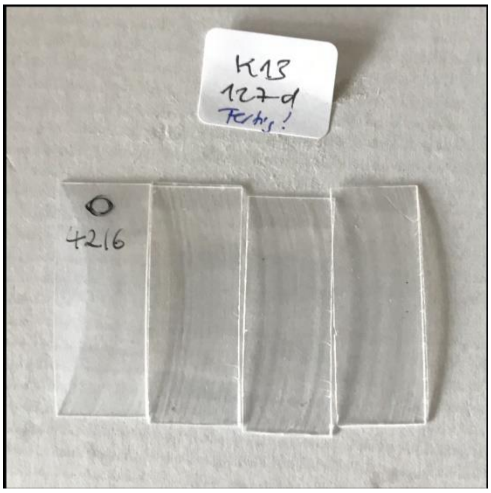

3.3 50% Ethanol at 20 °C

2 d

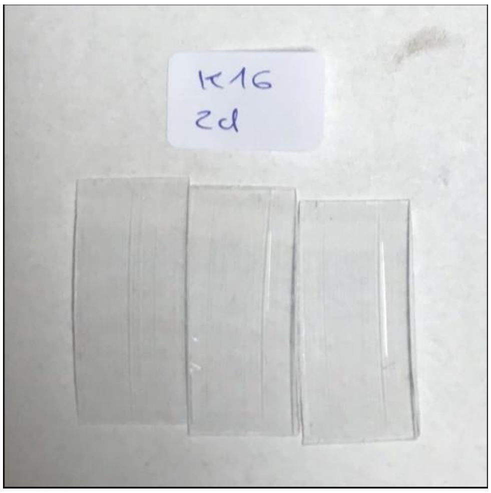

5 d

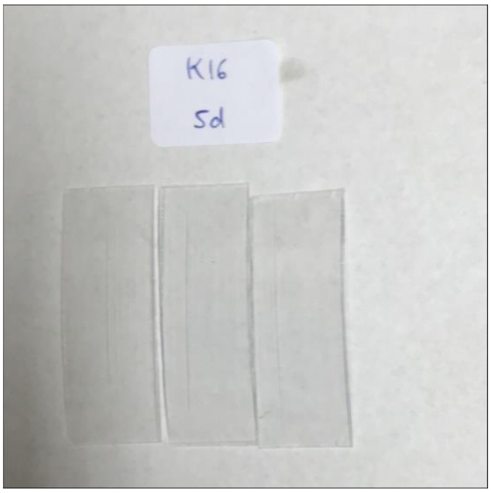

8 d

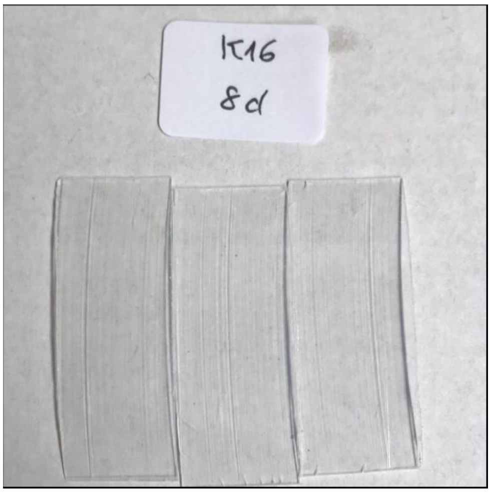

12 d

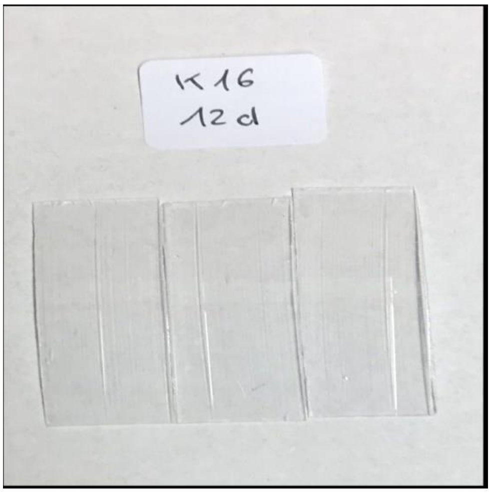

19 d

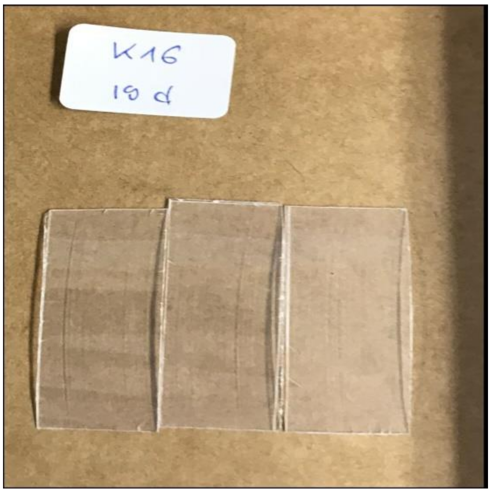

26 d

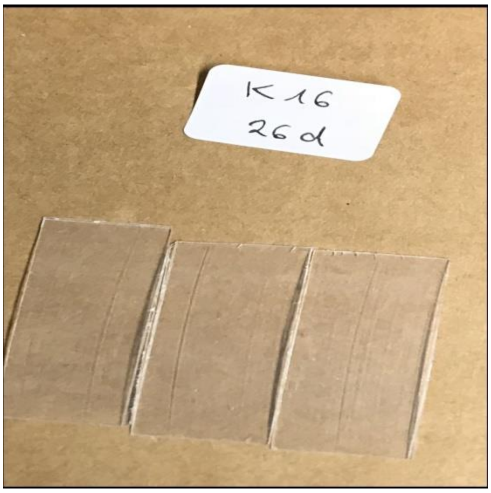

44 d	57 d
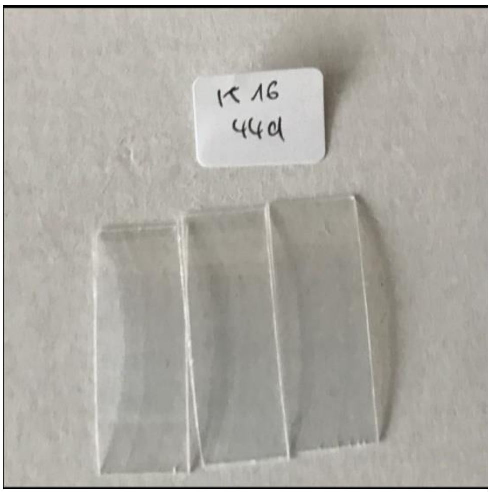	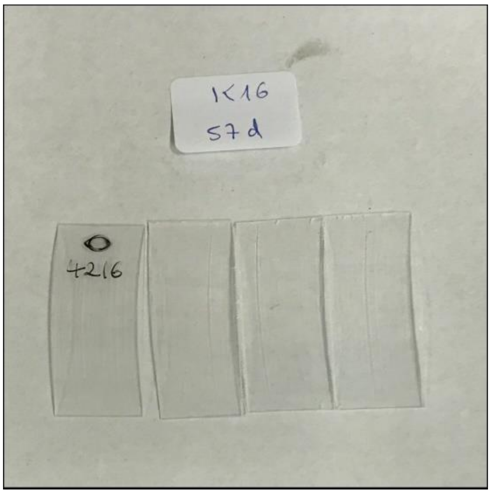
72 d	127 d
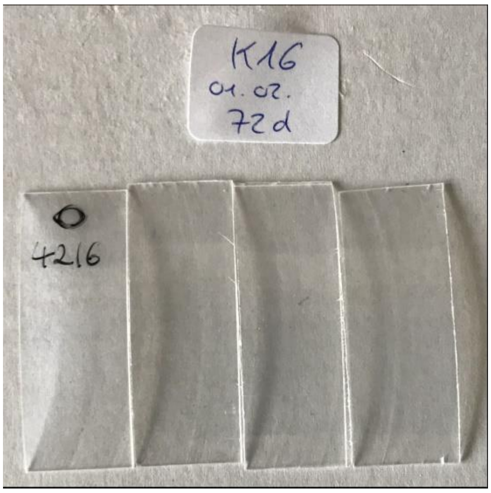	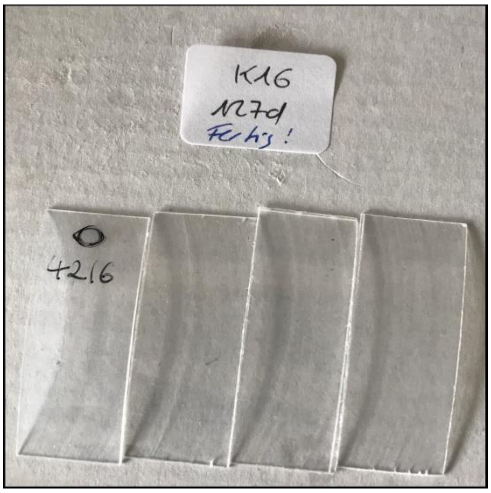

4 Contact with 20% Ethanol

4.1 20% Ethanol at 60 °C

2 d

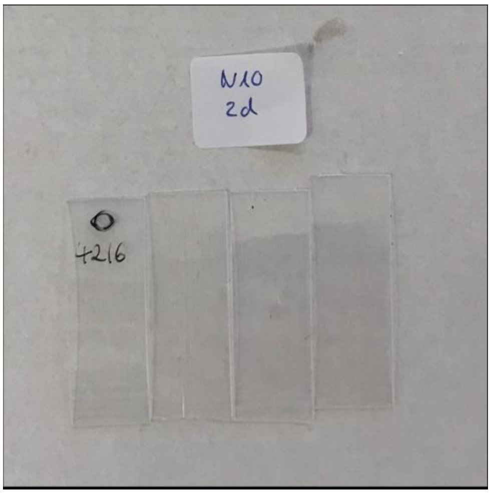

5 d

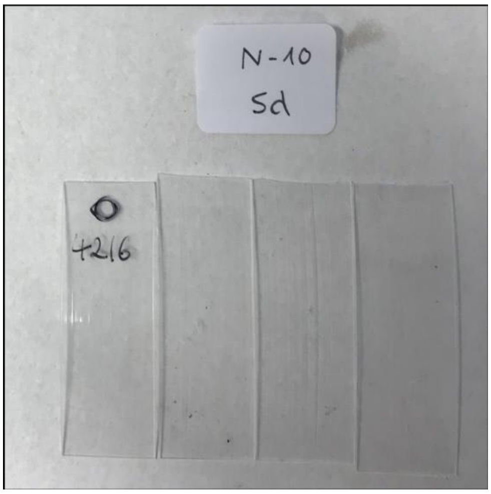

11 d

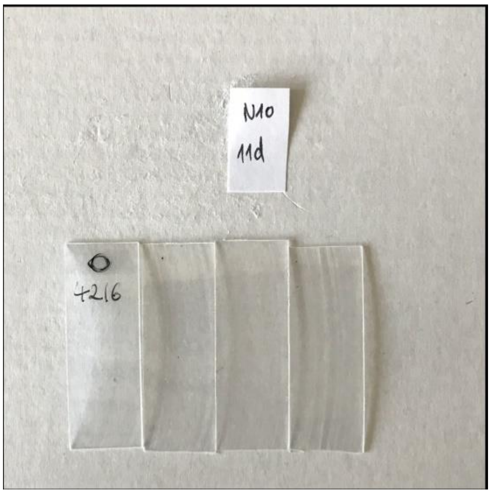

16 d

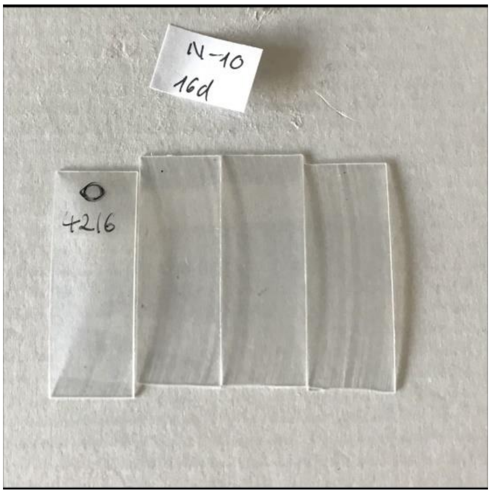

19 d 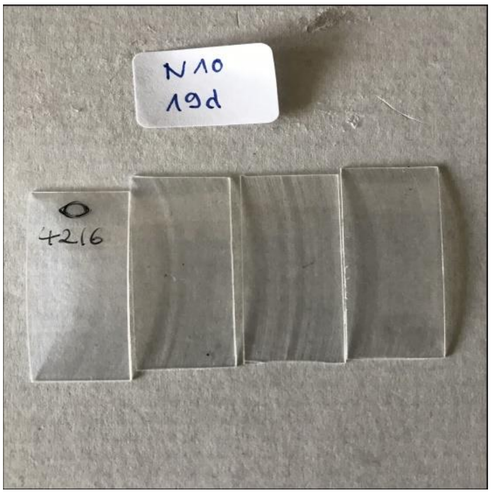	45 d 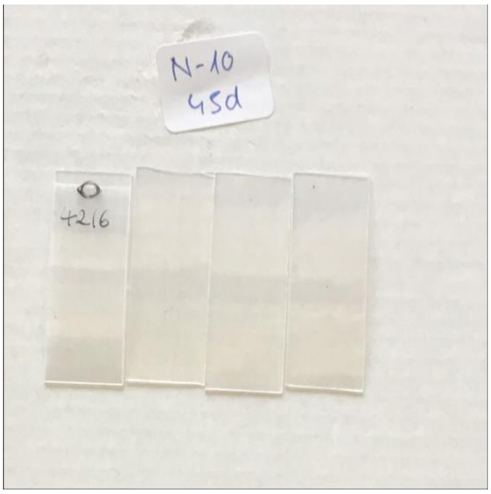
60 d 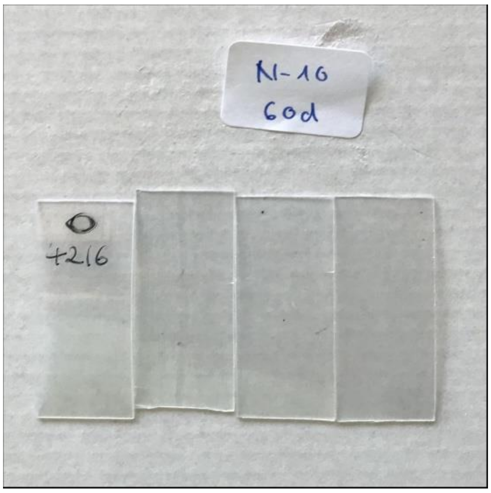	

4.2 20% Ethanol at 40 °C

<table>
<tr>
<td>2 d
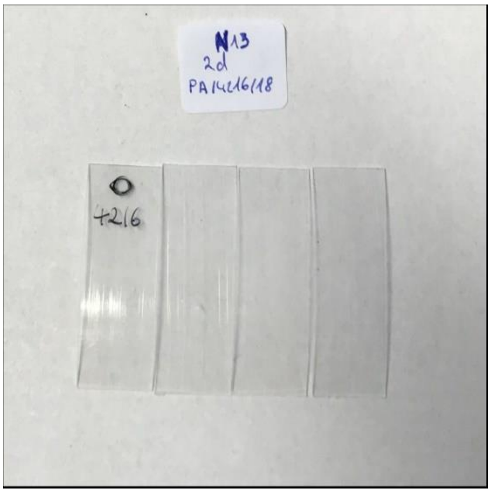
</td>
<td>5 d
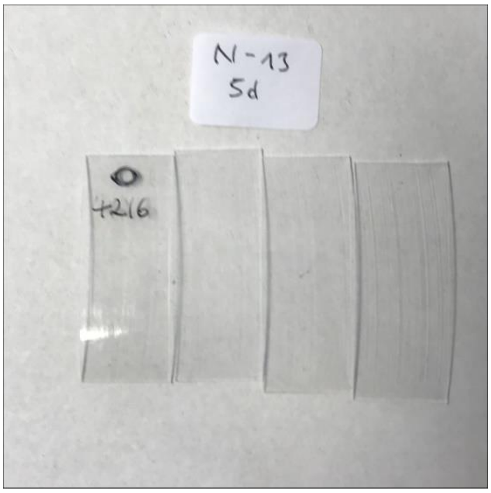
</td>
</tr>
<tr>
<td>11 d
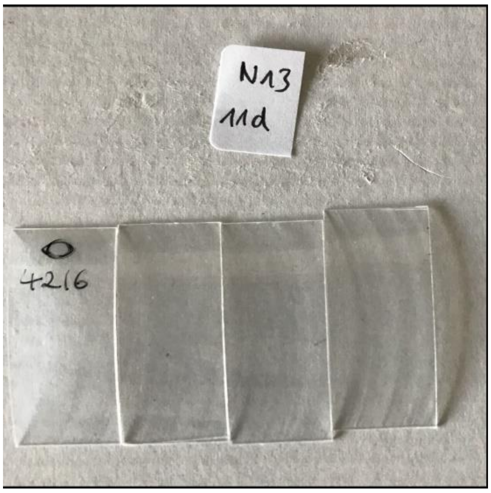
</td>
<td>16 d
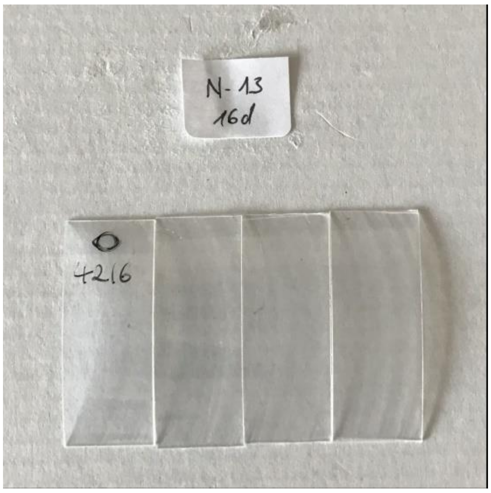
</td>
</tr>
<tr>
<td>45 d
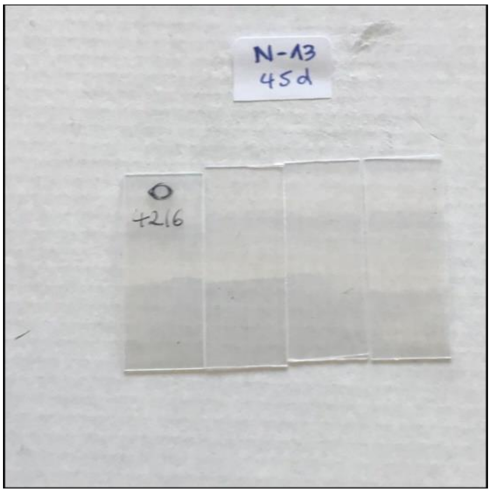
</td>
<td>89 d
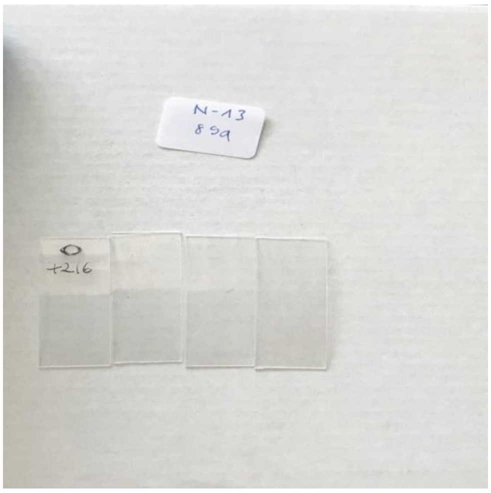
</td>
</tr>
</table>

4.3 20% Ethanol at 20 °C

2 d

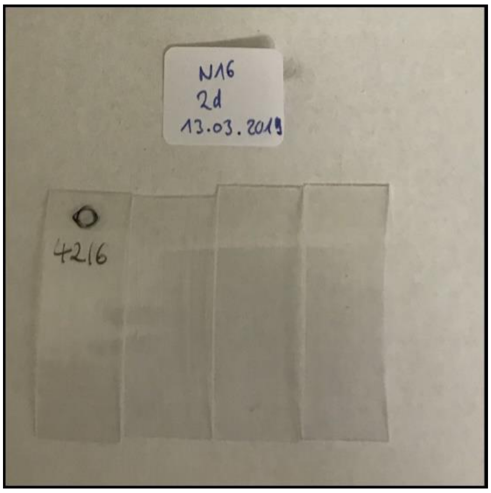

5 d

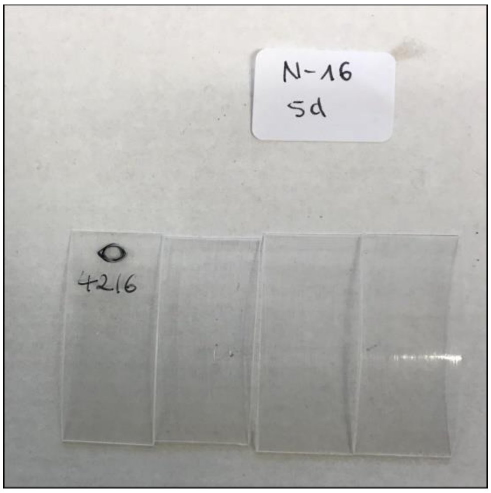

11 d

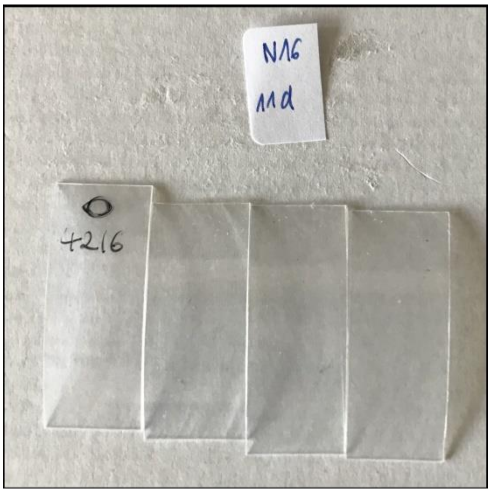

16 d

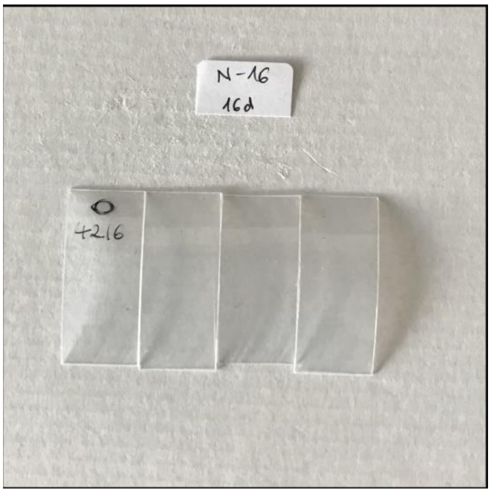

45 d

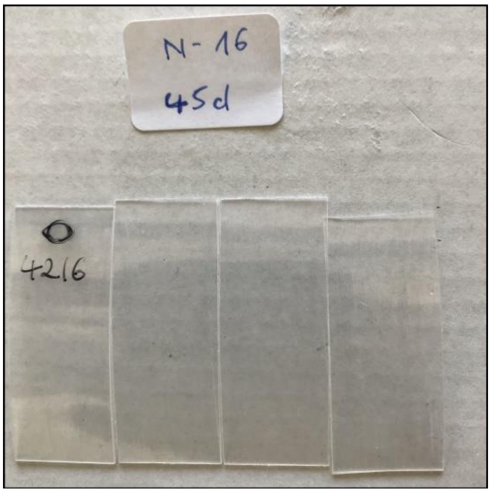

60 d

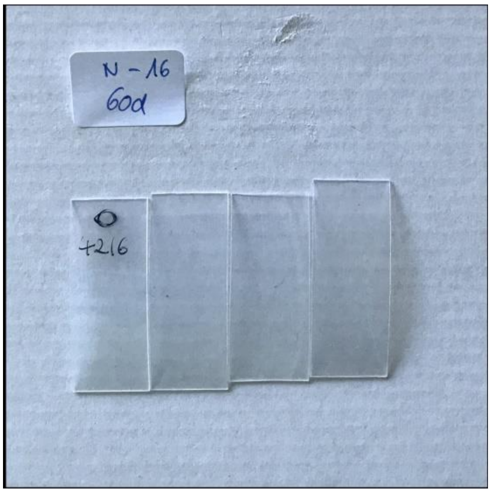

89 d<br>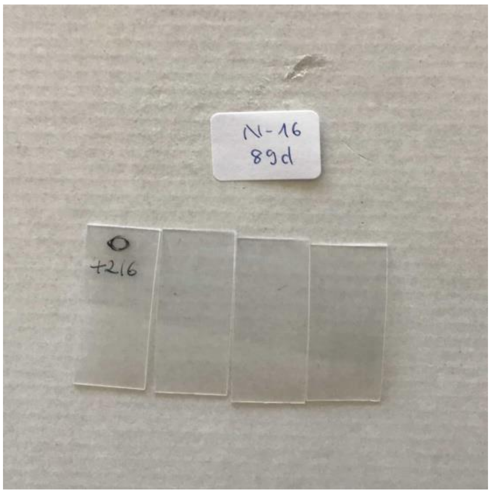 	

5 Contact with 10% Ethanol

5.1 10% Ethanol at 60 °C

2 d

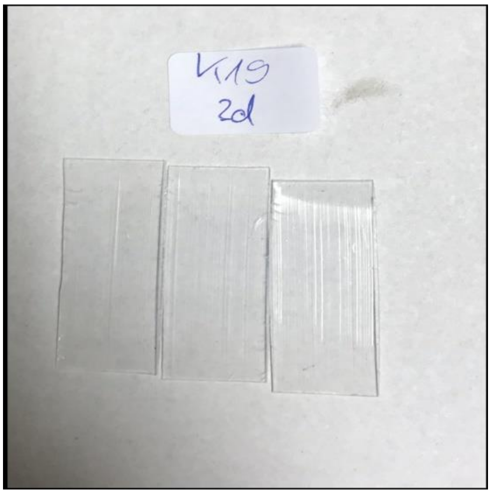

5 d

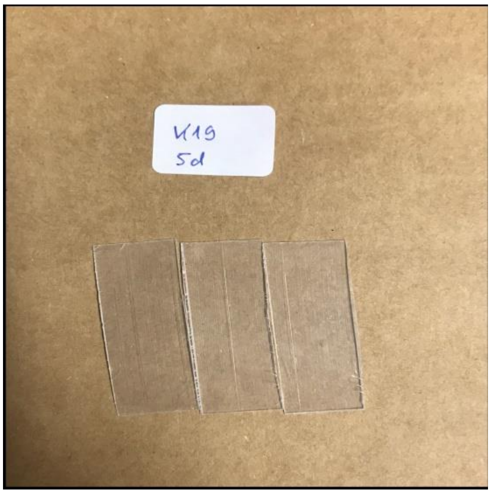

12 d

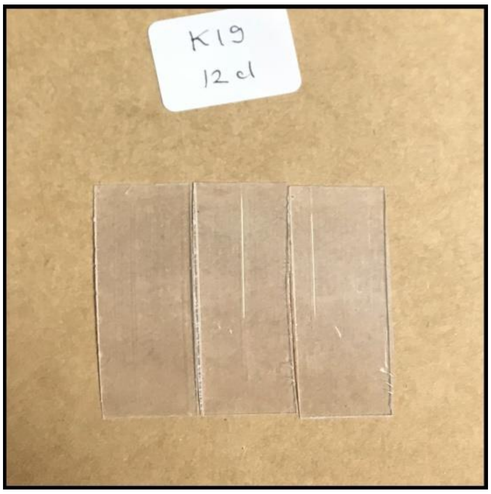

15 d

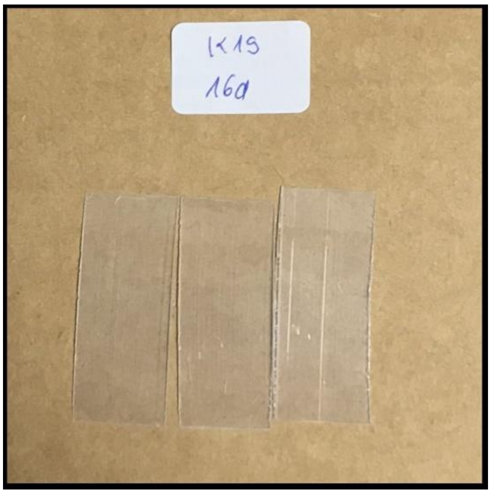

22 d

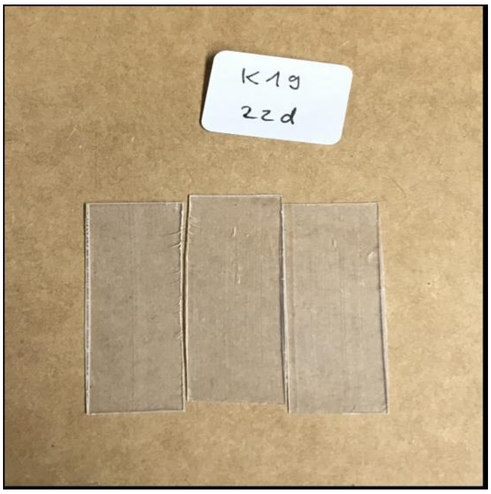

44 d

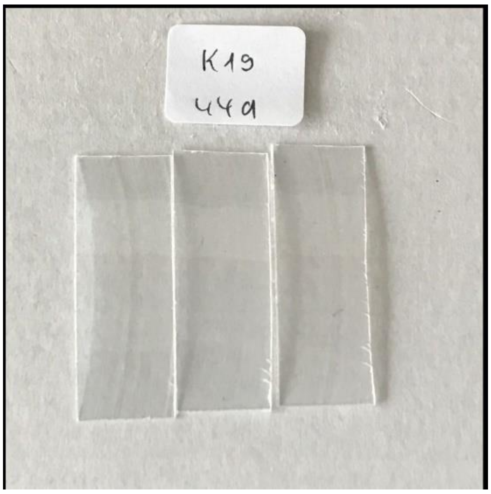

72 d

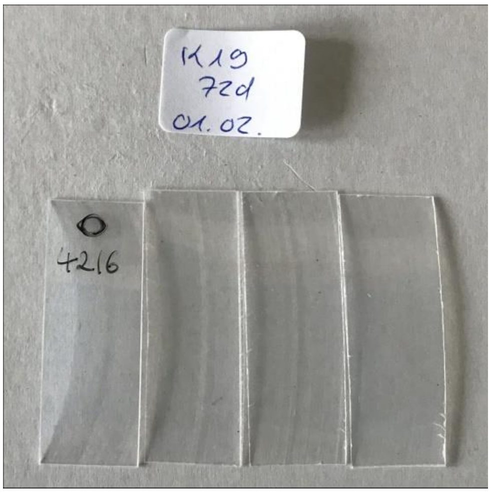

100 d

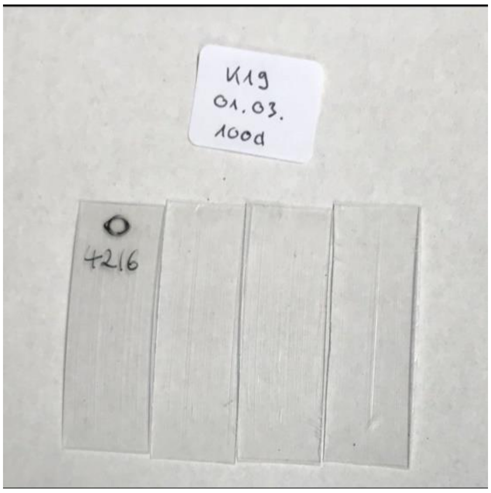

127

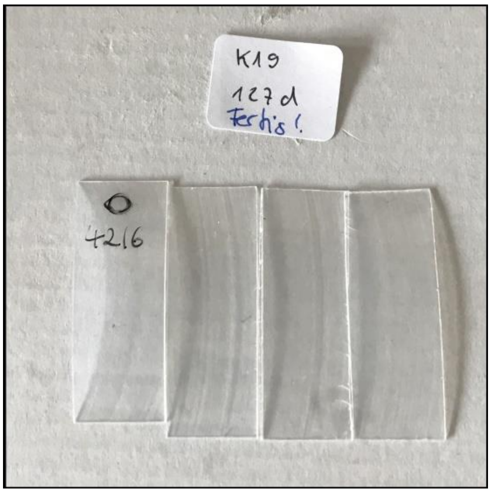

d

5.2 10% Ethanol at 40 °C

2 d<br>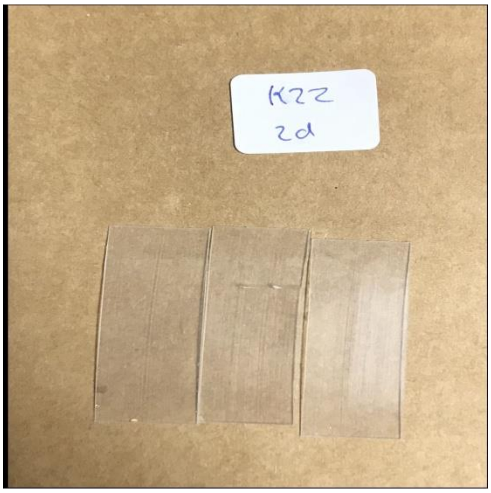	5 d<br>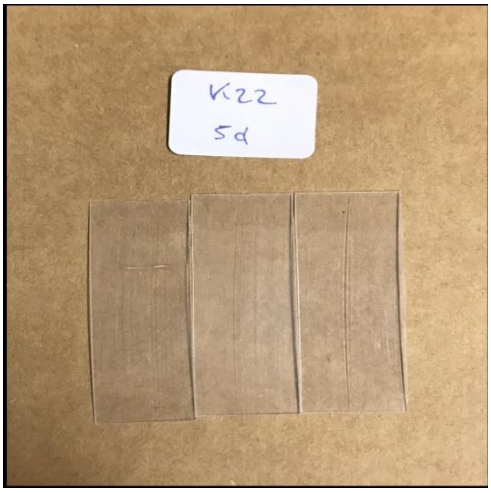
12 d<br>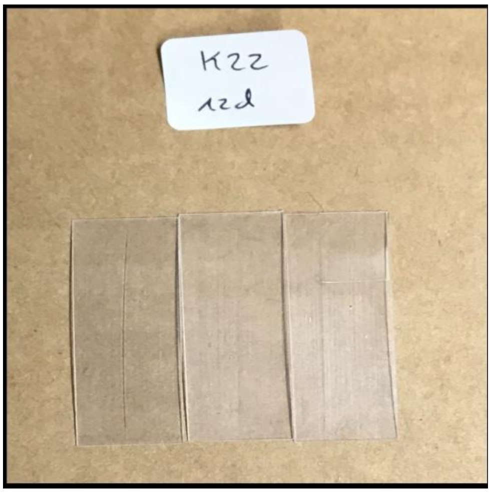	15 d<br>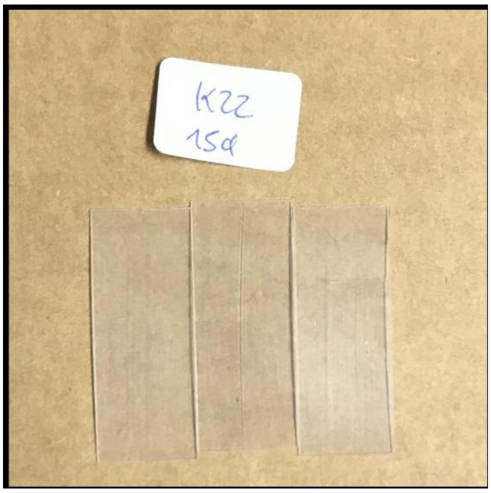
22 d<br>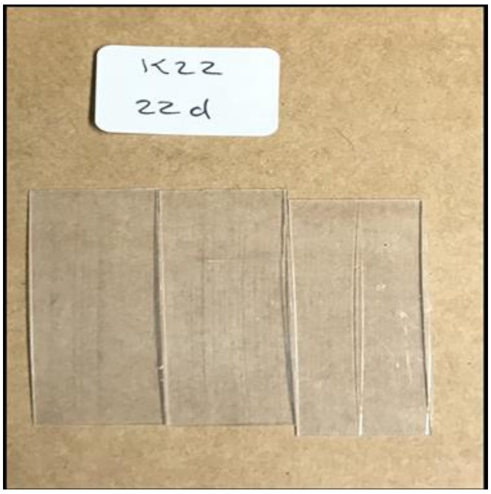	44 d<br>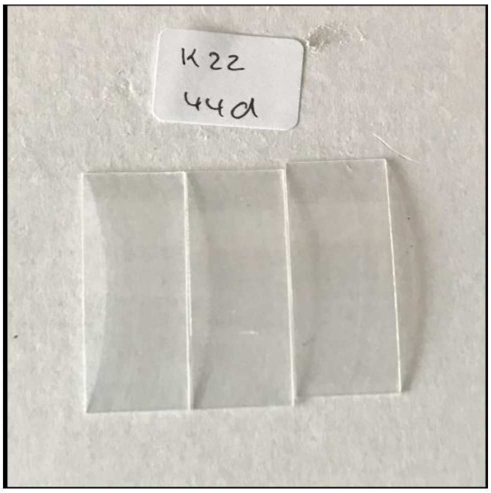

57 d

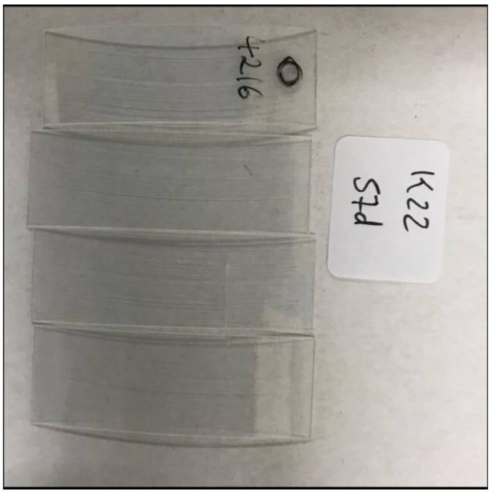

72 d

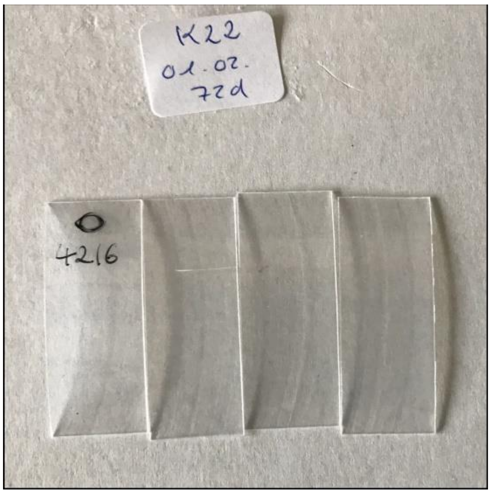

100 d

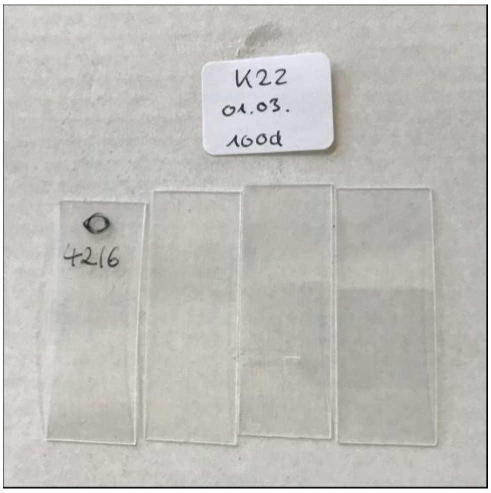

127 d

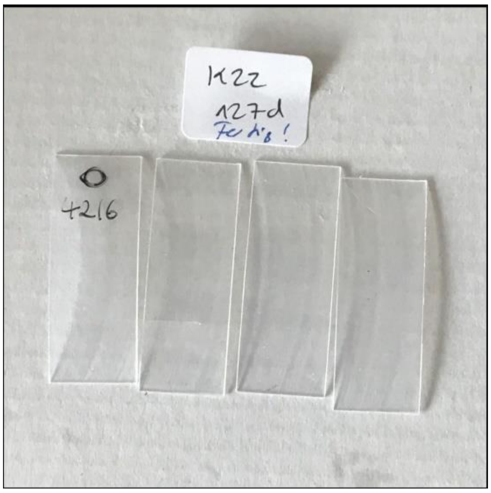

5.3 10% Ethanol at 20 °C

2 d

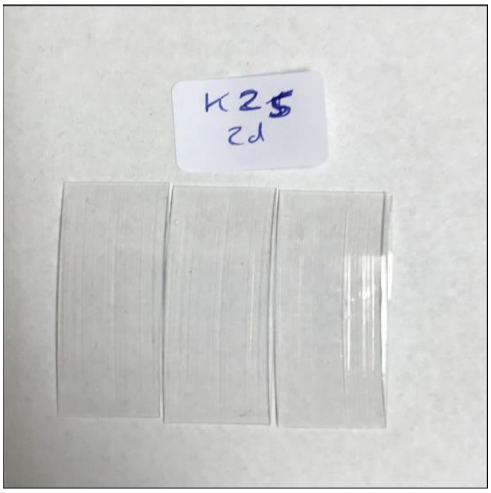

5 d

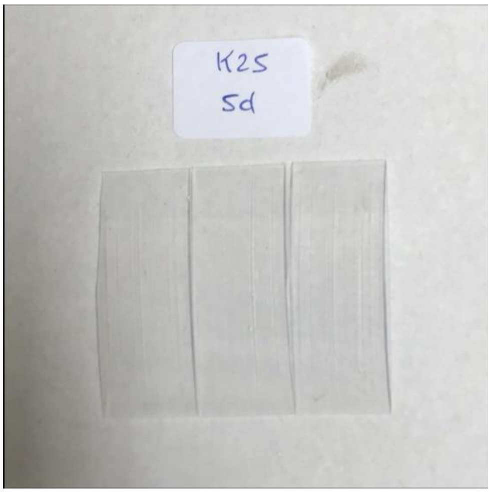

12 d

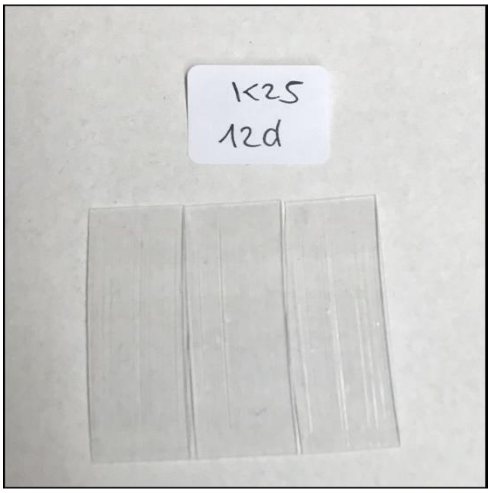

15 d

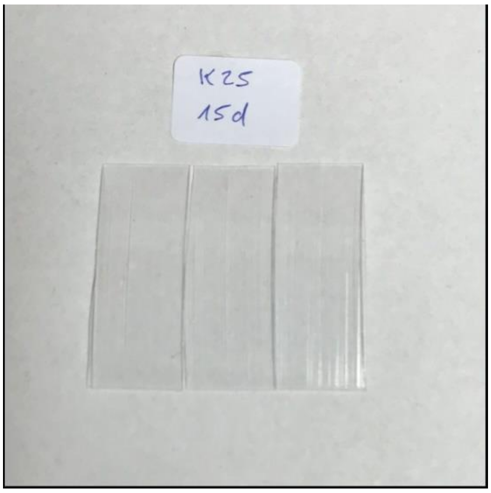

22 d

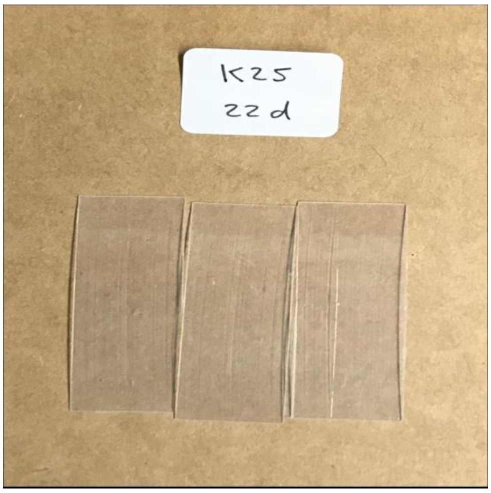

44 d

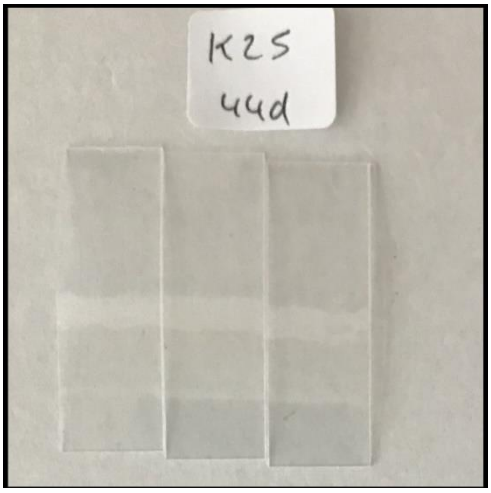

57 d

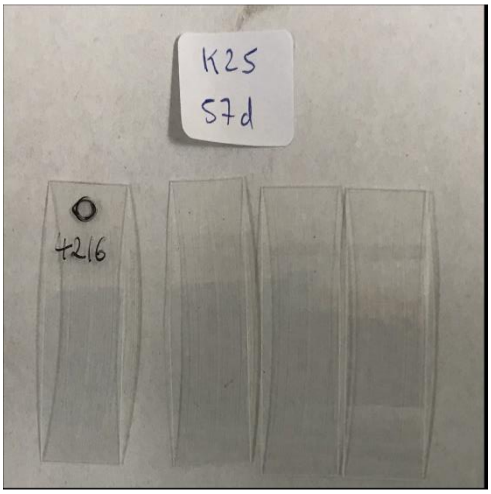

72 d

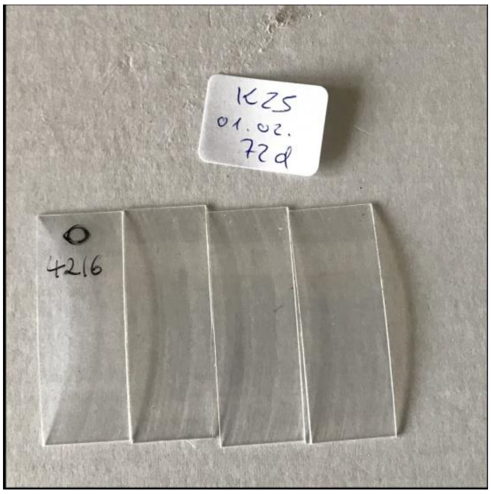

100 d

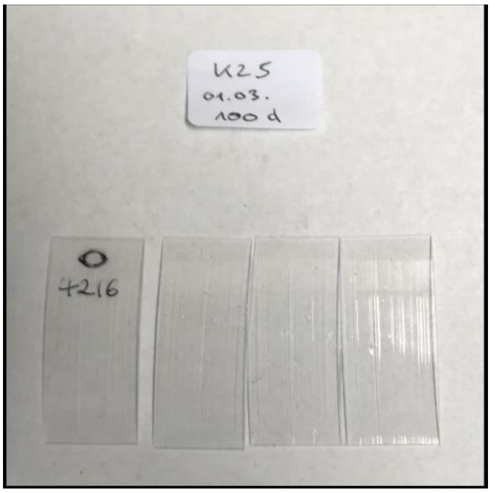

127 d

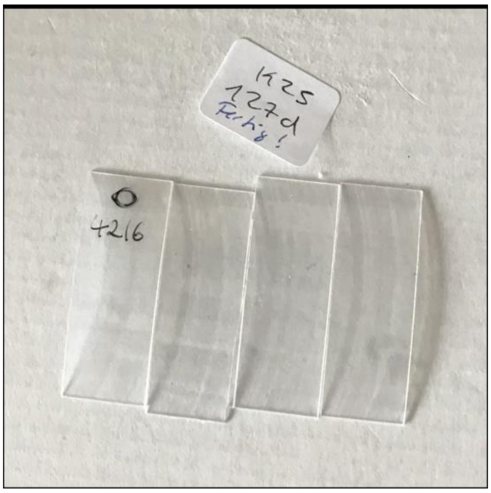

6 Contact with 3% Acetic acid

6.1 3% Acetic acid at 60 °C

2 d	5 d
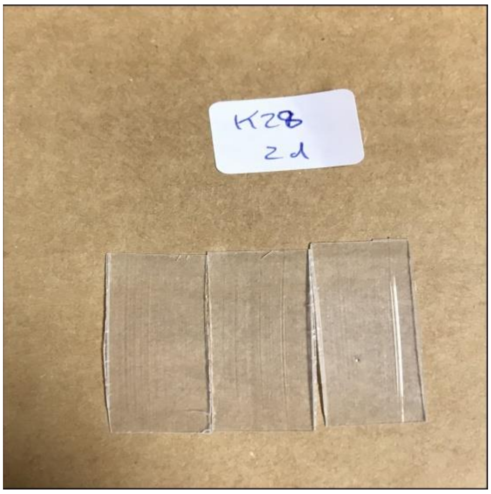	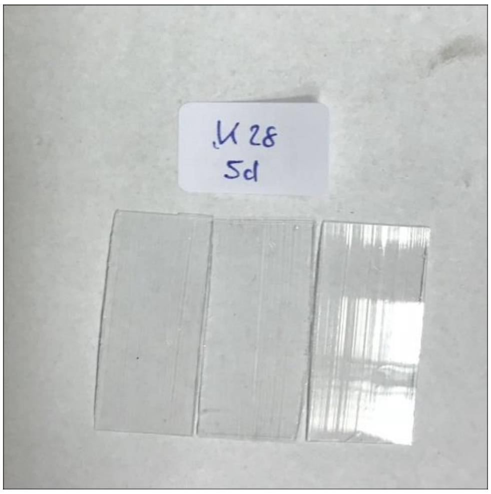
12 d	16 d
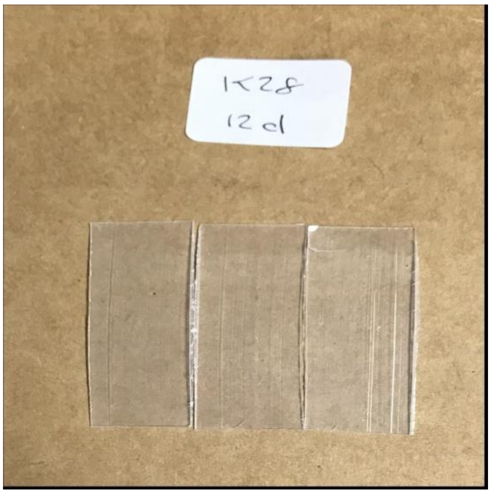	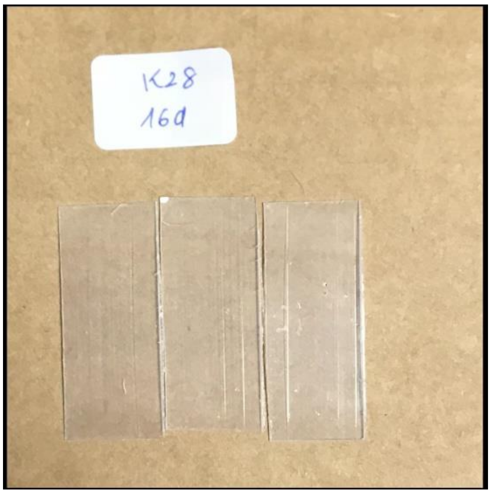

22 d

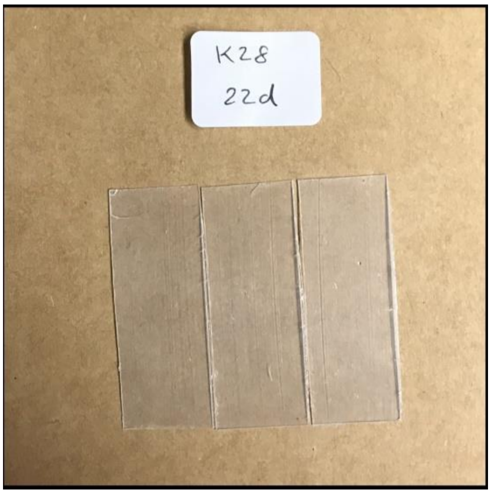

44 d

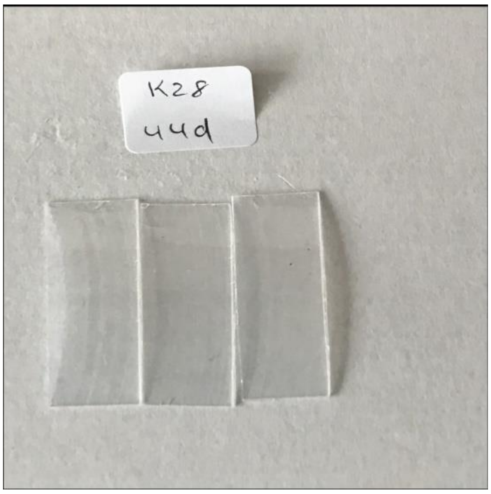

57 d

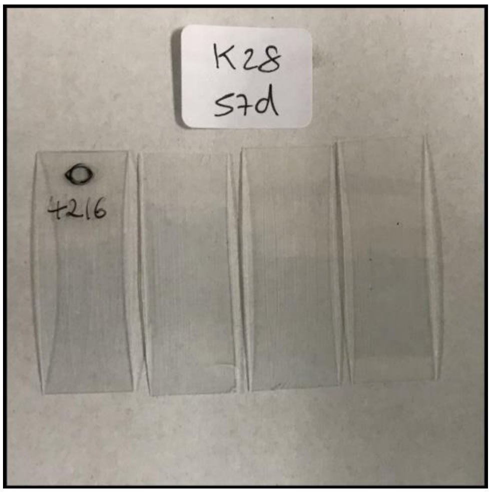

72 d

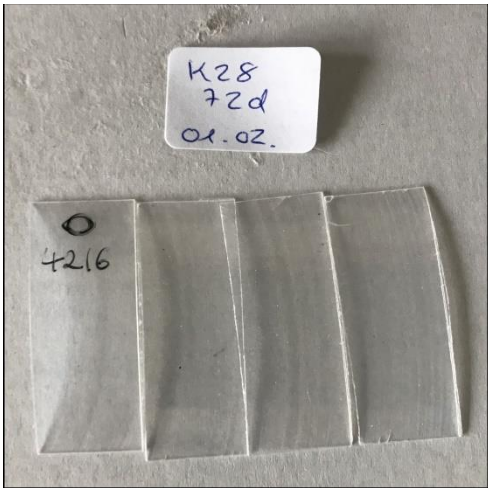

100 d

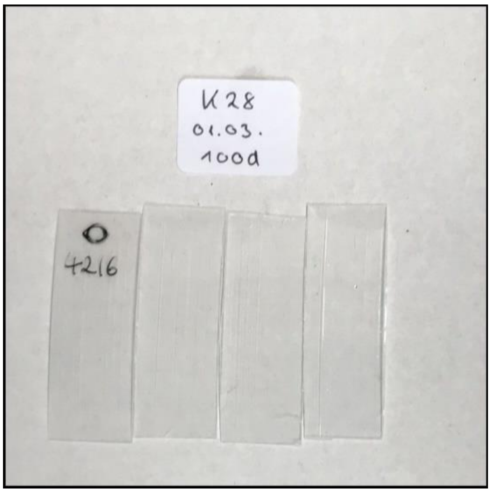

127 d

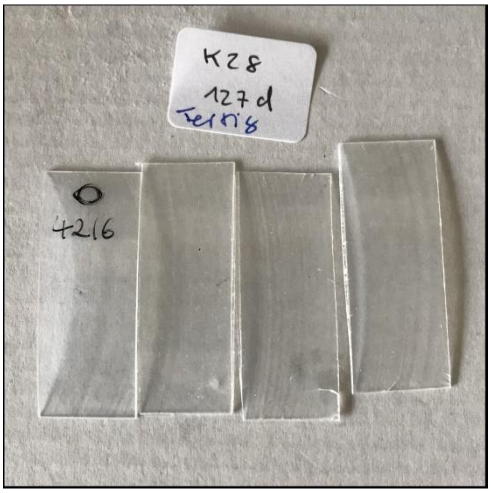

6.2 3% Acetic acid at 40 °C

2 d

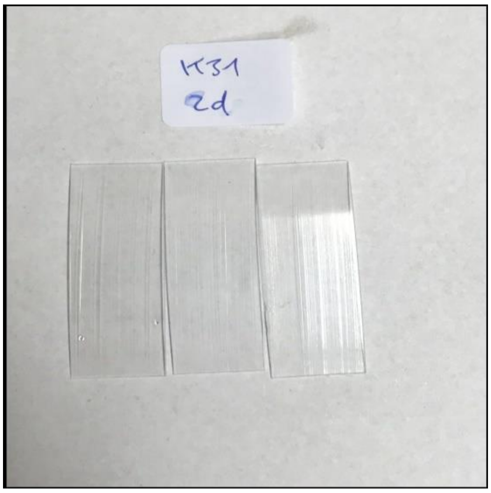

5 d

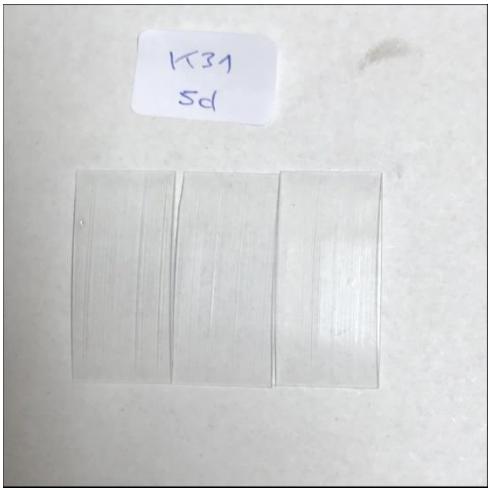

12 d

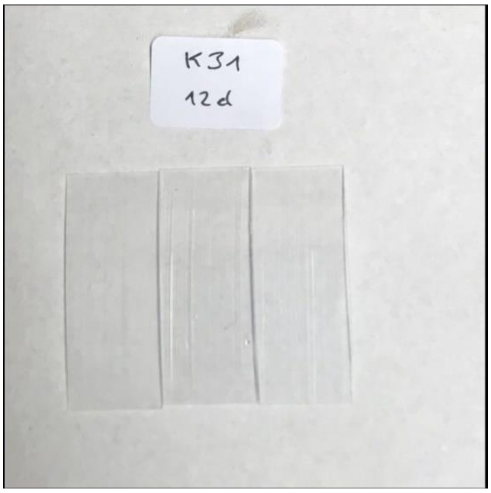

22 d

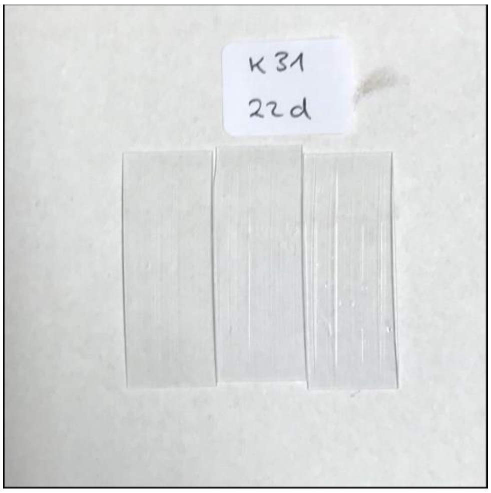

44 d

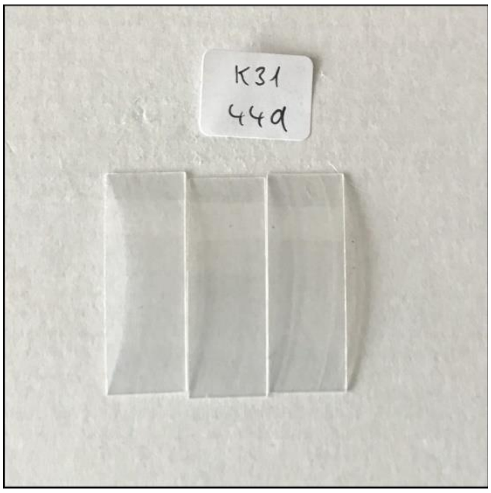

57 d

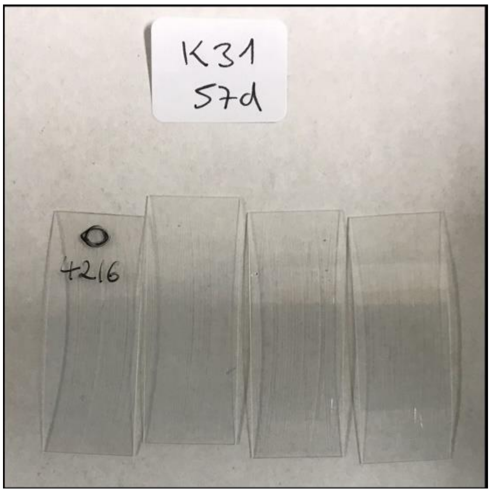

127 d 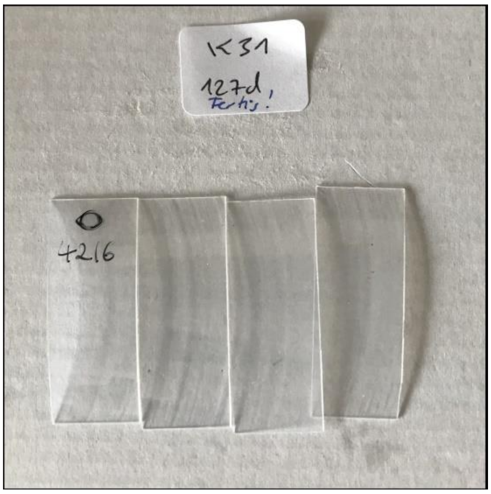	

6.3 3% Acetic acid at 20 °C

2 d

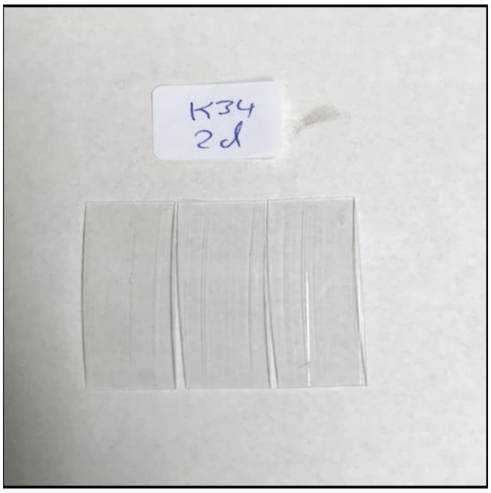

5 d

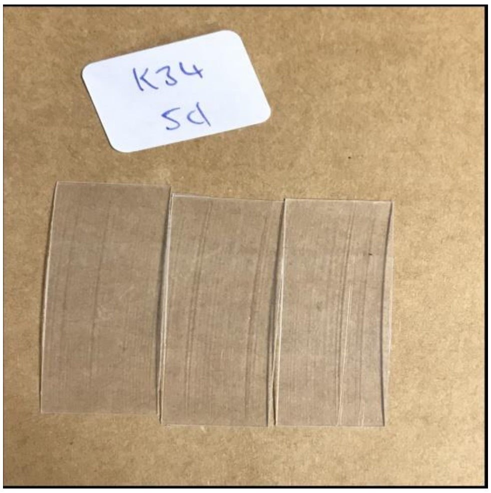

12 d

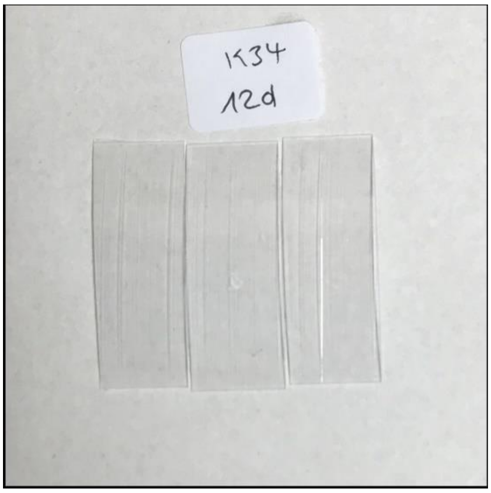

15 d

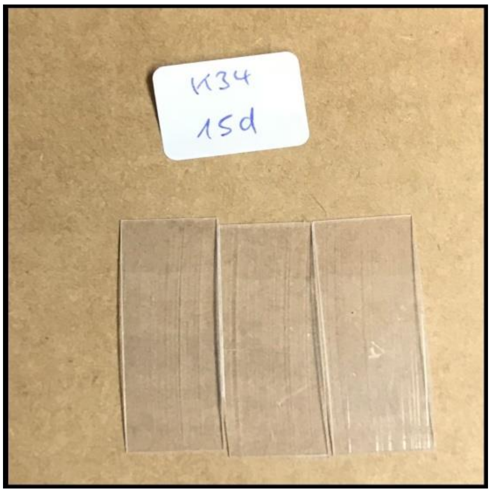

22 d

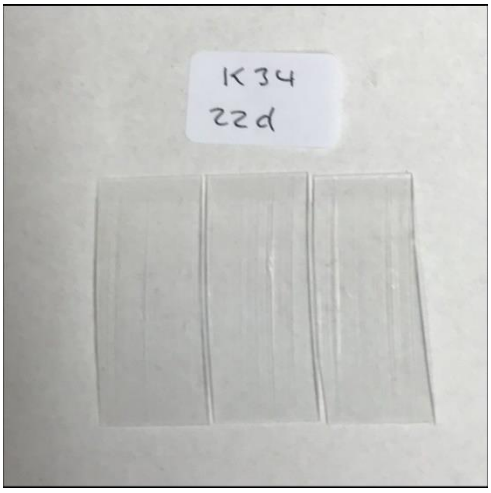

44 d

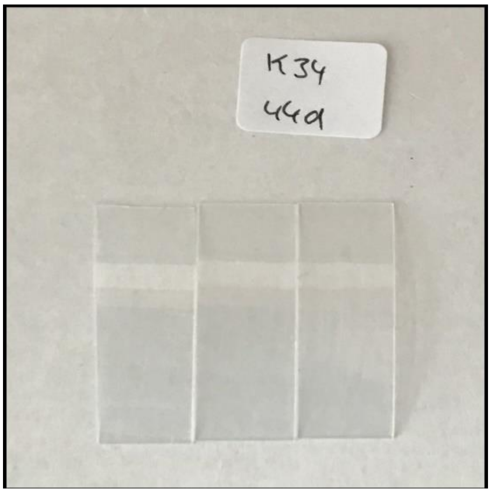

57 d

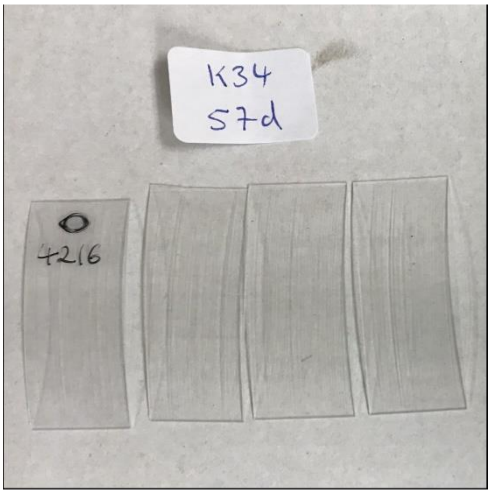

72 d

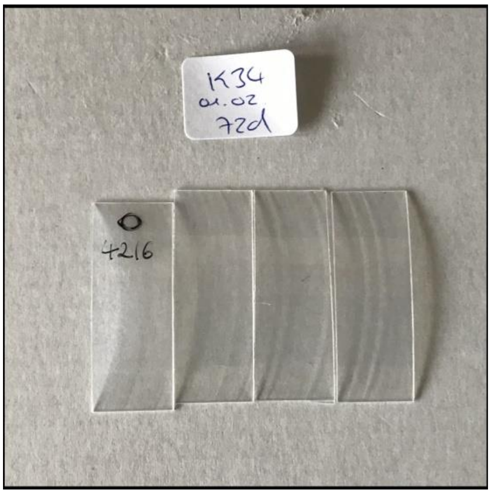

100 d

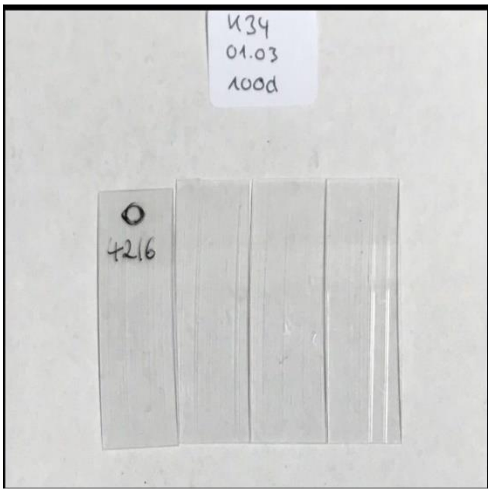

127 d

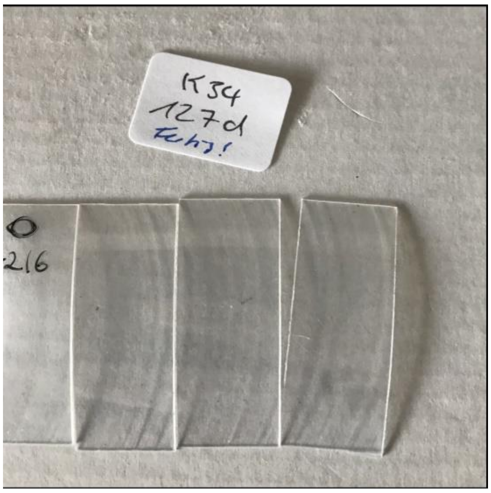

7 Contact with real foods

7.1 Milk, cream and olive oil 60 d at 20 °C

Milk	Cream	Olive oil
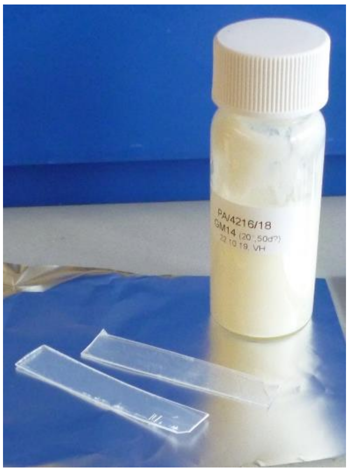	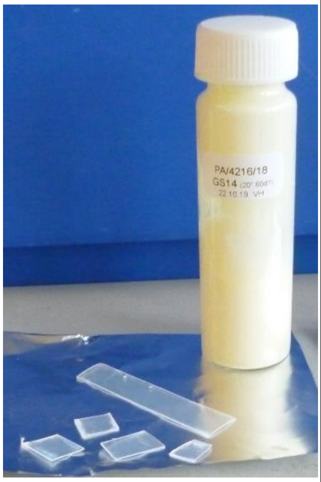	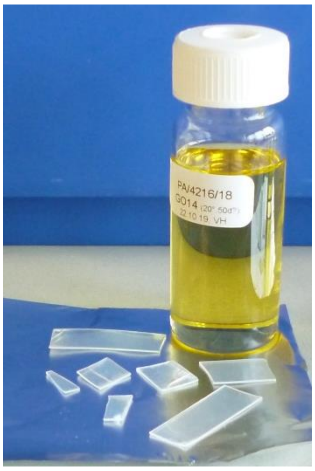

7.2 Milk, cream and olive oil 10 d at 40 °C

Milk	Cream	Olive oil
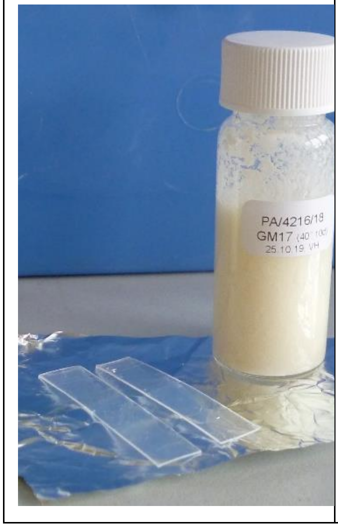	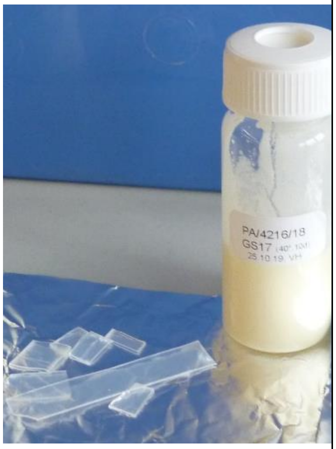	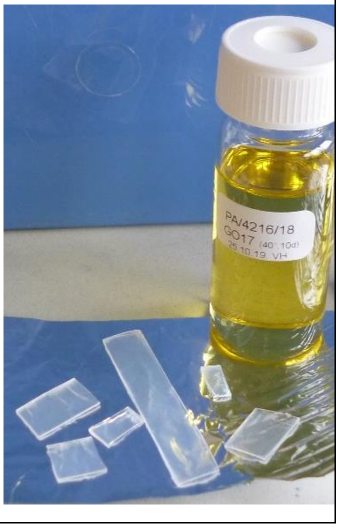

7.3 Lard at 5 °C

10 d	20 d	50 d
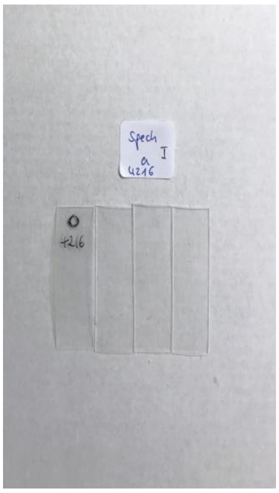	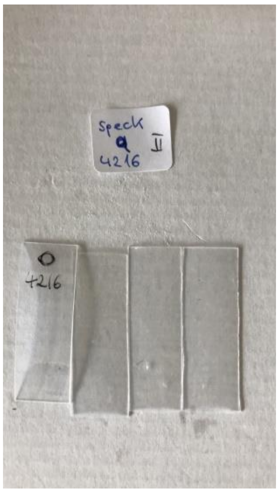	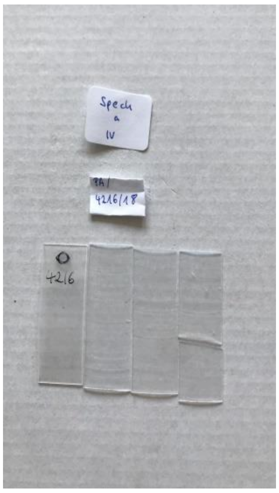

7.4 Butter at 5 °C

10 d	20 d	50 d
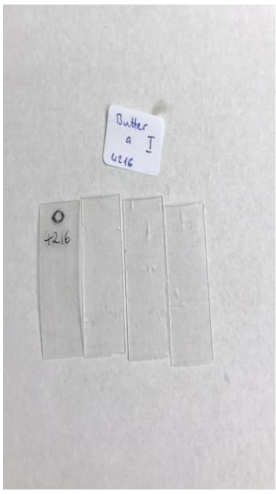	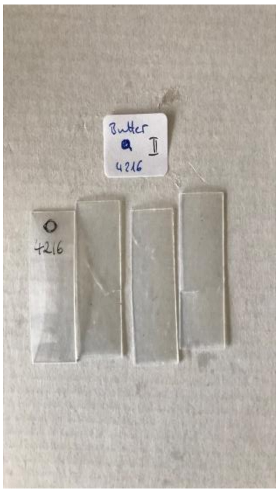	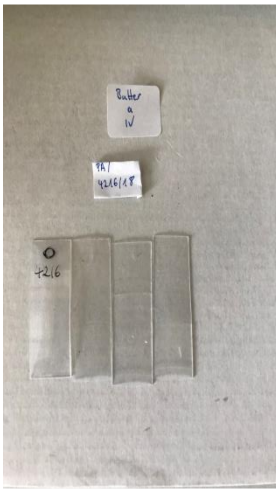

7.5 Fish oil at 20 °C

10 d	20 d	50 d
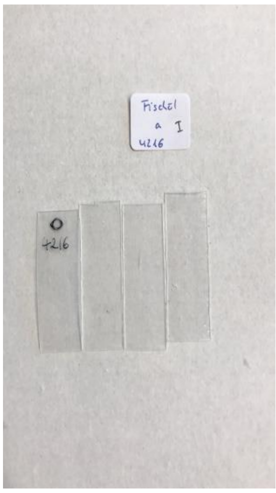	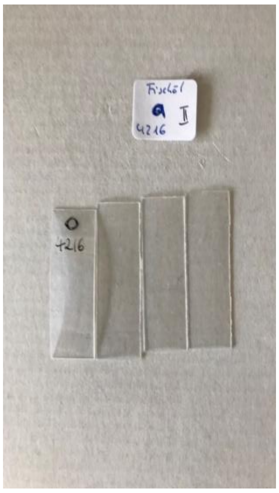	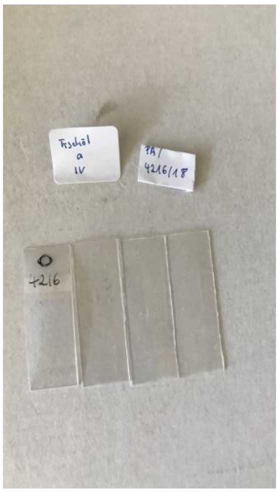

7.6 Miglyol® 812 at 20 °C

10 d	20 d	50 d
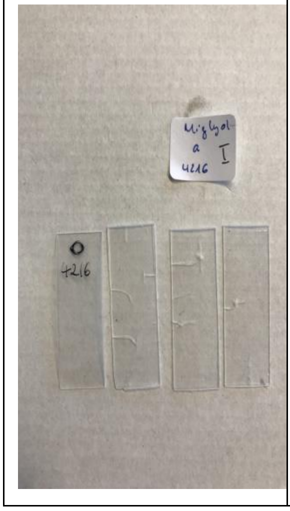	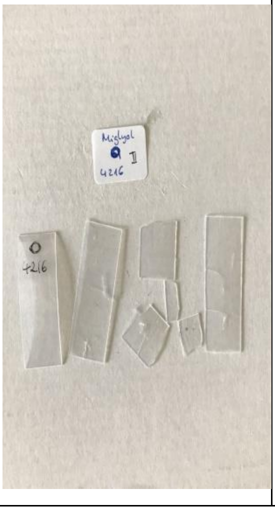	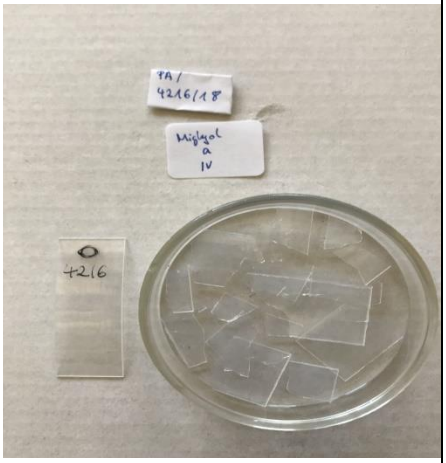

7.7 Clear orange juice at 20 °C

10 d	20 d	50 d
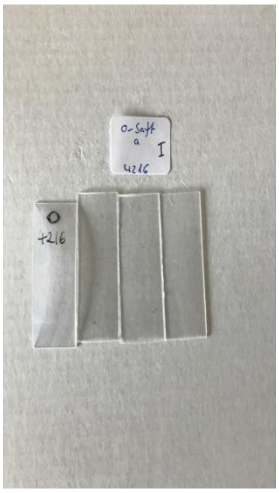	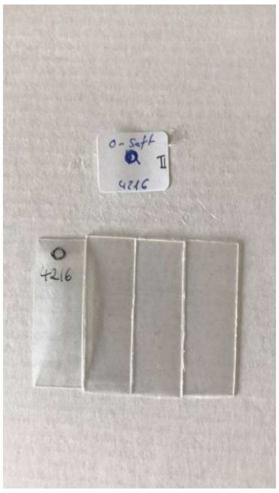	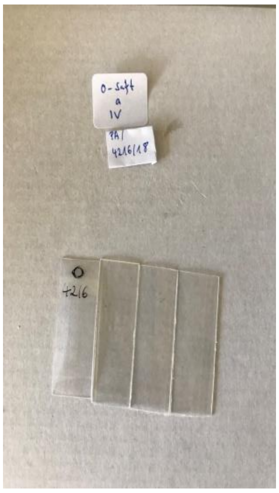

7.8 Ground coffee beans at 20 °C

10 d	20 d	50 d
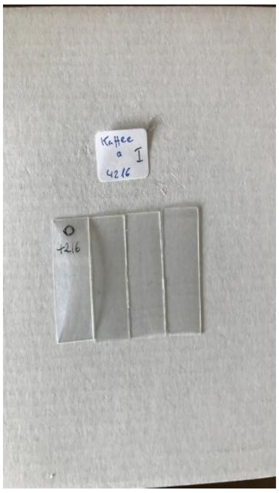	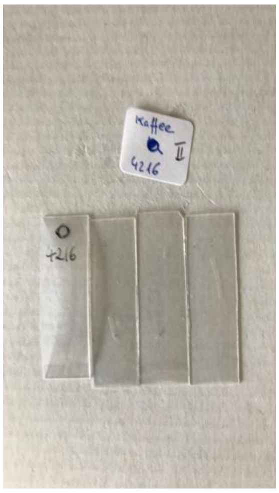	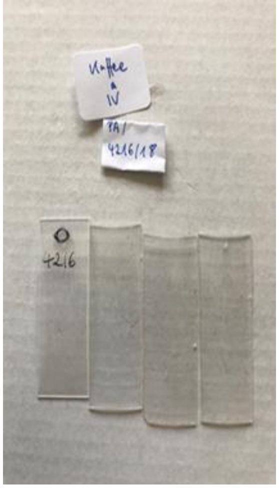

7.9 Noodles at 20 °C

10 d	20 d	50 d
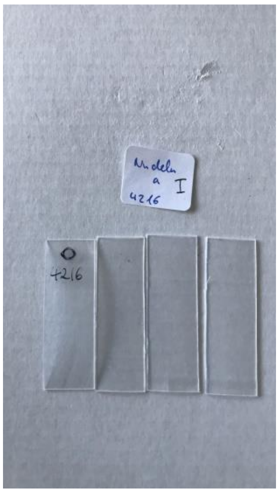	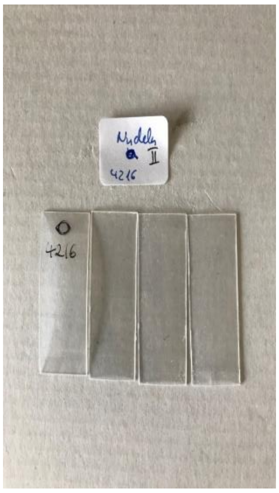	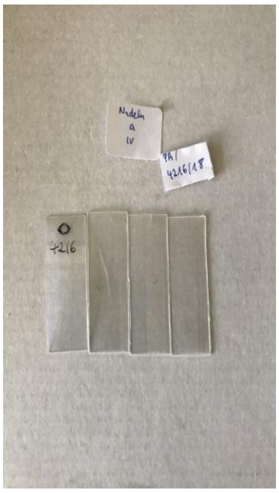

7.10 Oat flakes at 20 °C

10 d	20 d	50 d
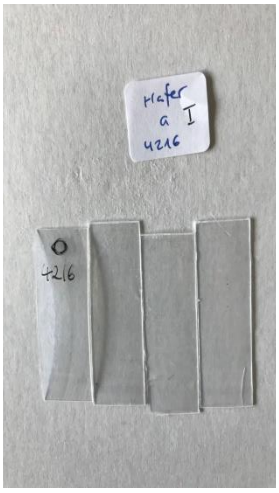	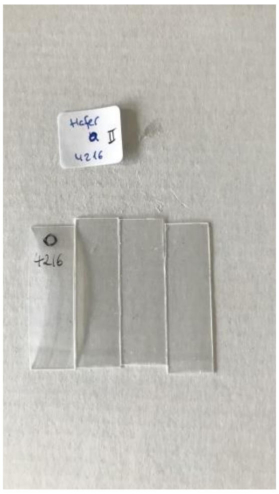	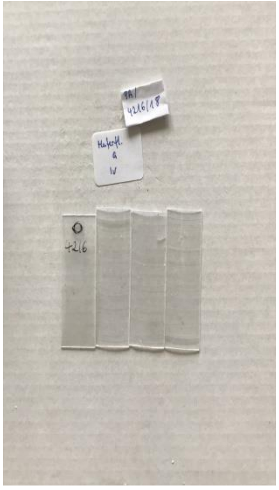

7.11 Wheat loops at 20 °C

10 d	20 d	50 d
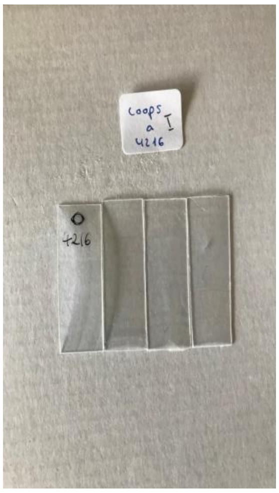	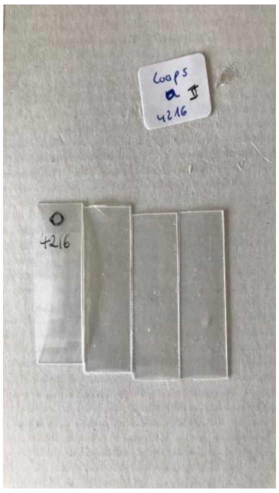	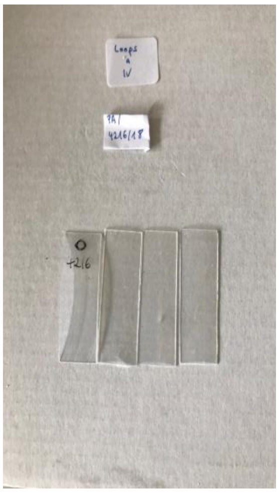

7.12 Water at 20 °C

10 d	20 d	50 d
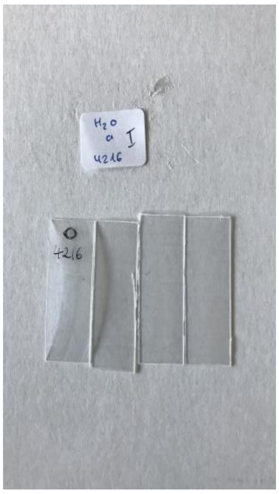	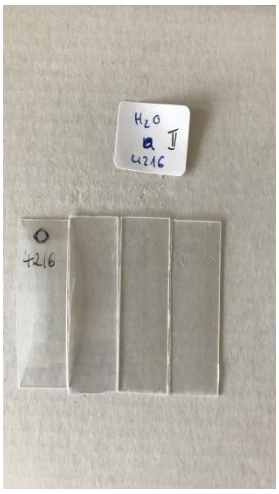	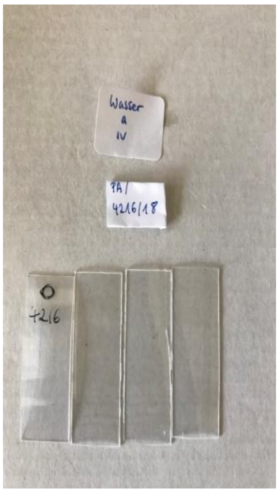

7.13 Water at 100 °C

10 min	20 min	30 min
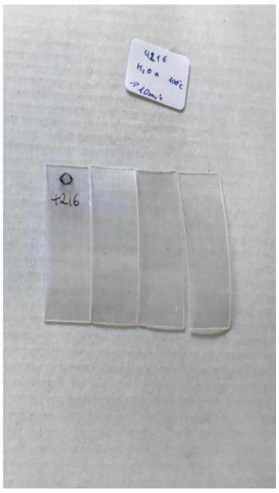	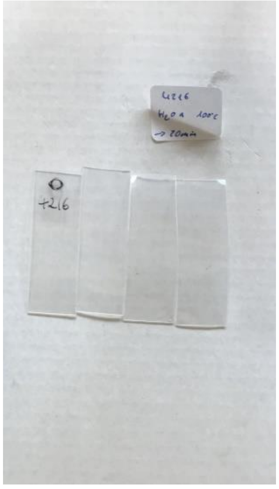	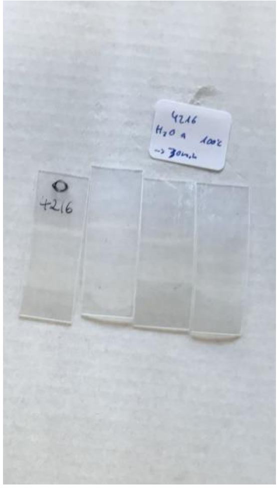

7.14 Water at 80 °C

10 min	20 min	30 min
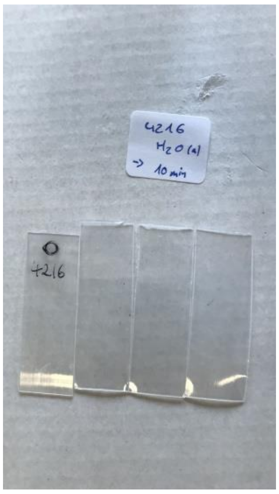	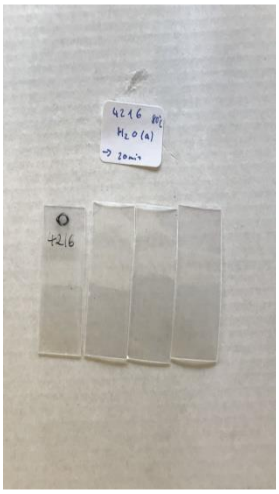	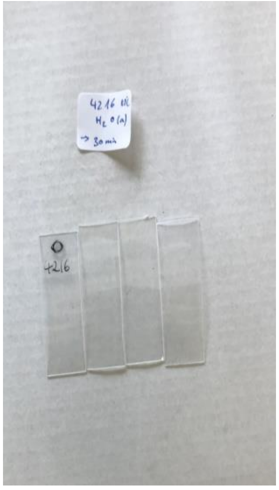

7.15 Water at 60 °C

10 min	20 min	30 min
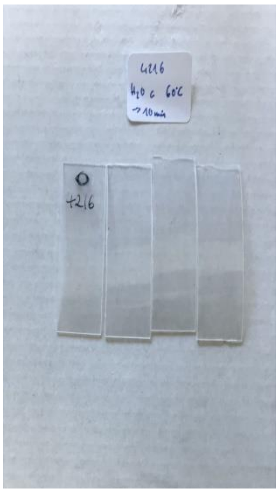	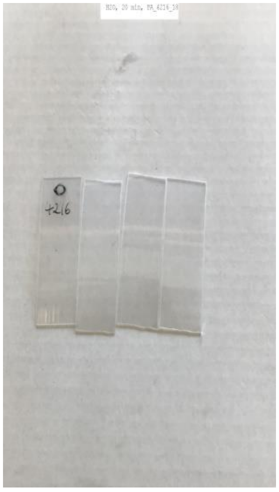	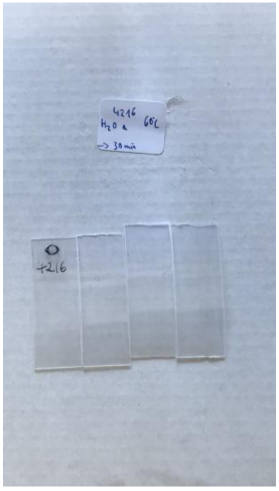

7.16 Water at 40 °C

10 min	20 min	30 min
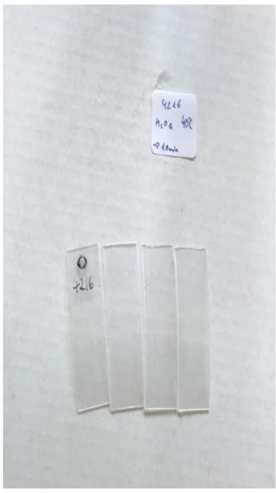	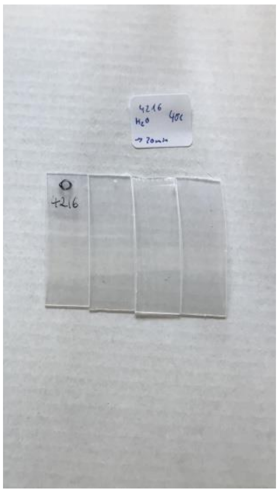	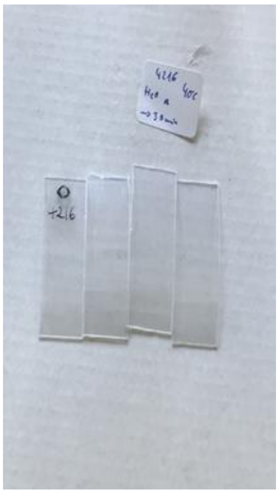

7.17 Brewed coffee at 90 °C

10 min 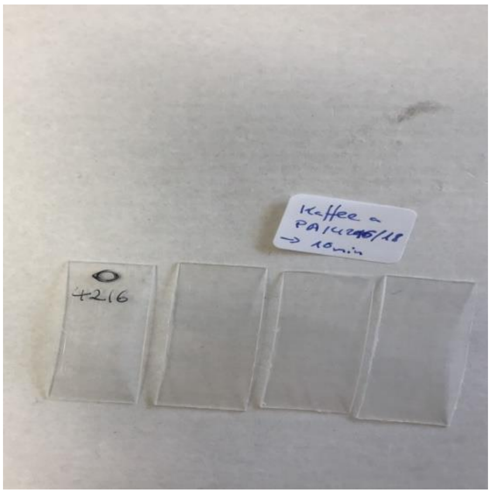	20 min 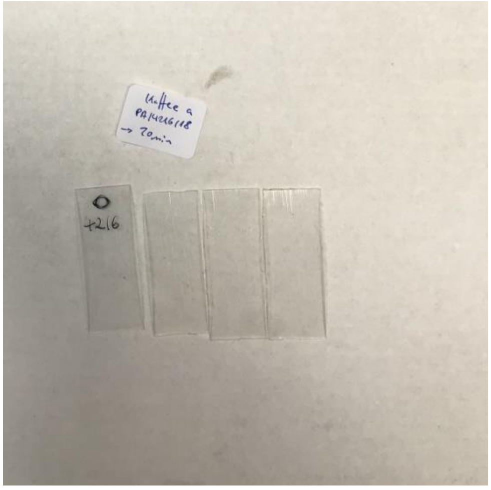
30 min 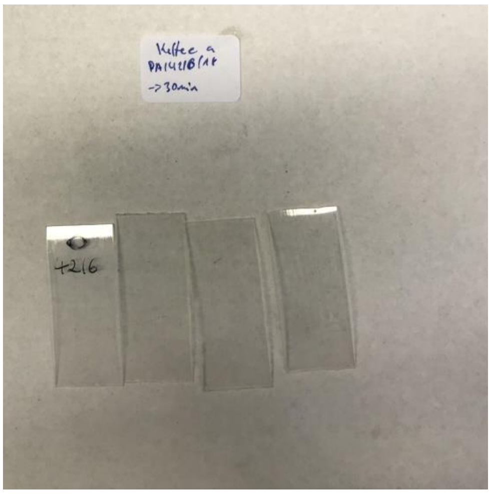	

HIPS (high impact polystyrene)

8 Contact with Isooctane

8.1 Isooctane at 60 °C

4 h 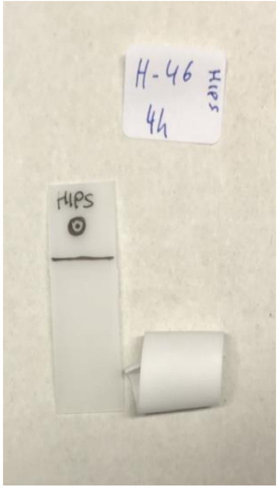	8 h 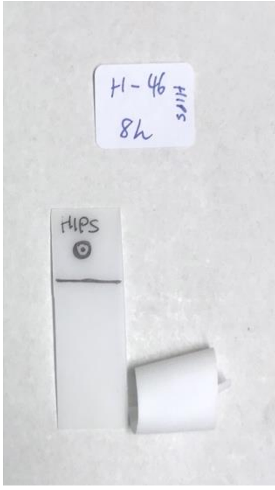	2 d 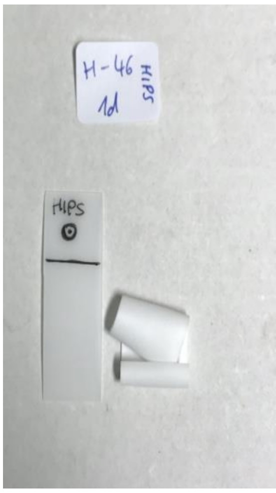
5 d 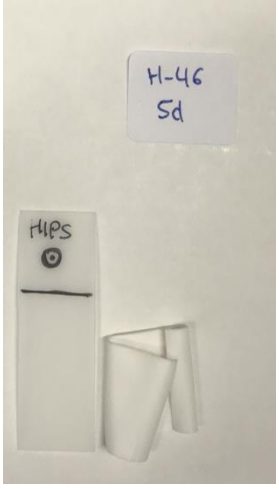	12 d 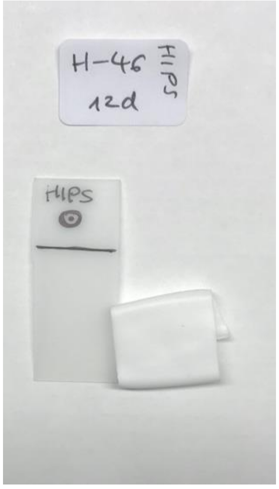	16 d 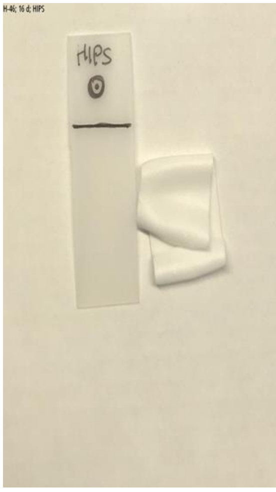

30 d 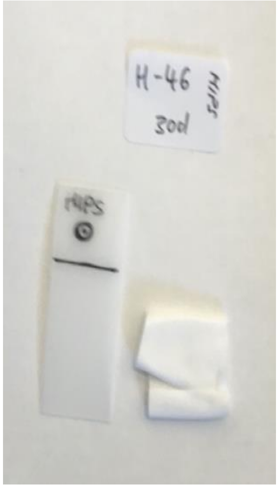	44 d 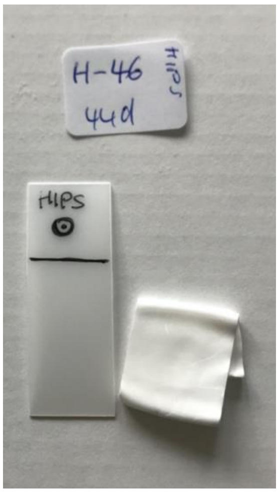	64 d 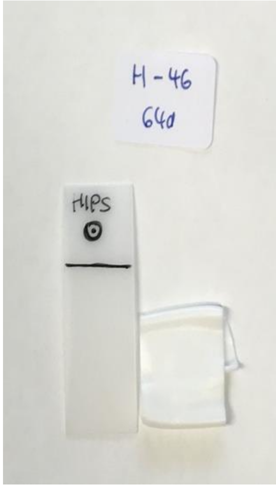
92 d 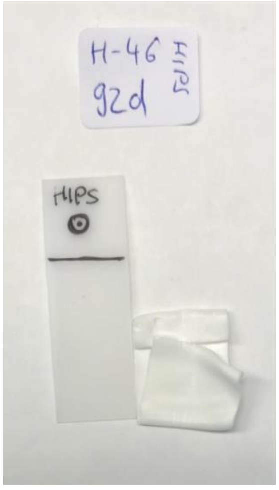	126 d 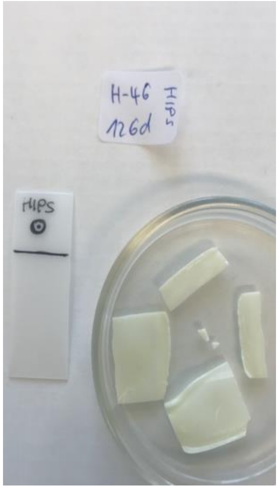	159 d 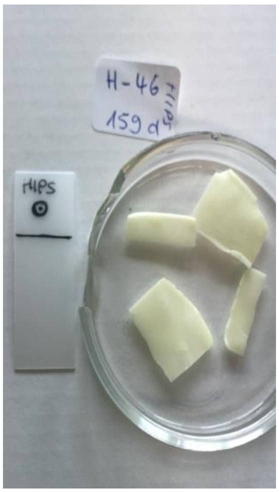
201 d 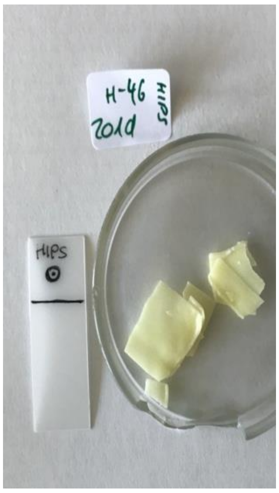	273 d 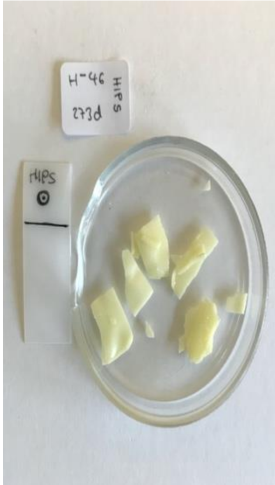	

8.2 Isooctane at 40 °C

<table>
<tr>
<td>4 h
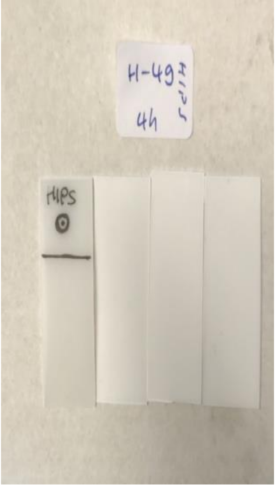
</td>
<td>8 h
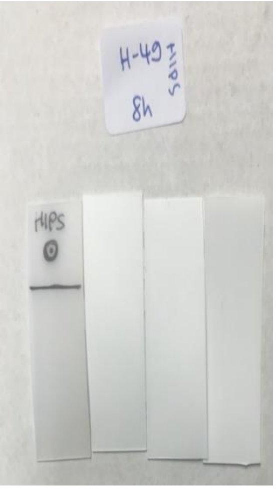
</td>
<td>2 d
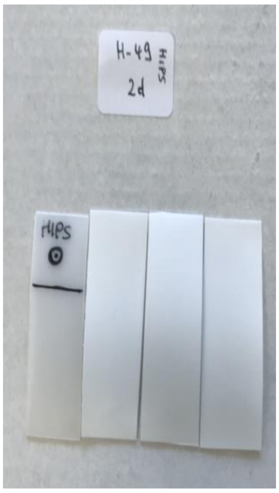
</td>
</tr>
<tr>
<td>5 d
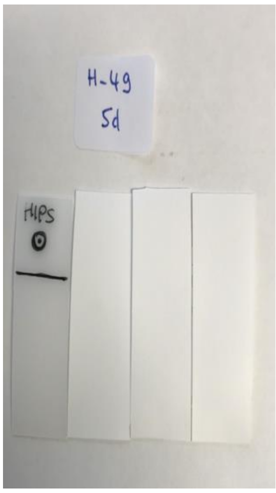
</td>
<td>12 d
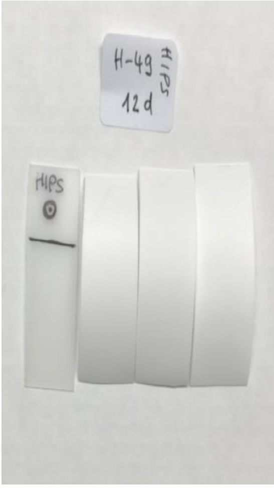
</td>
<td>16 d
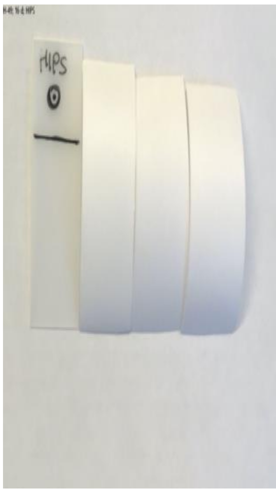
</td>
</tr>
<tr>
<td>30 d
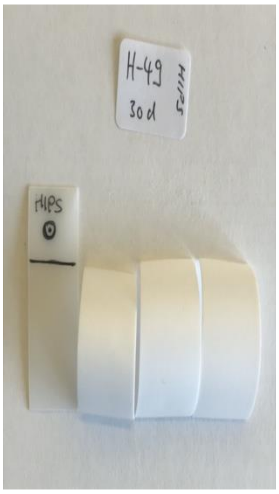
</td>
<td>44 d
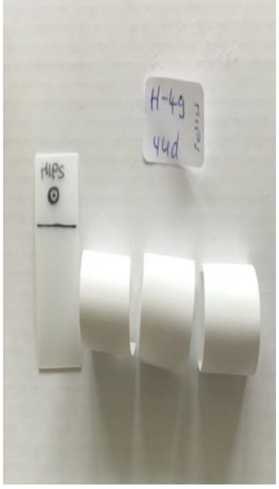
</td>
<td>64 d
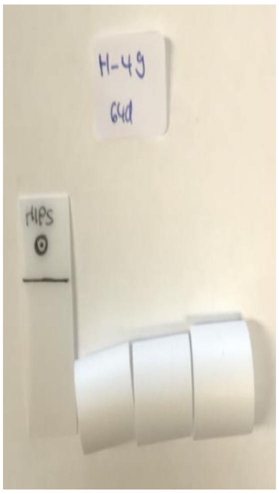
</td>
</tr>
</table>

92 d	126 d	159 d
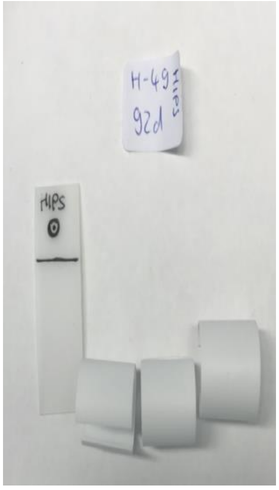	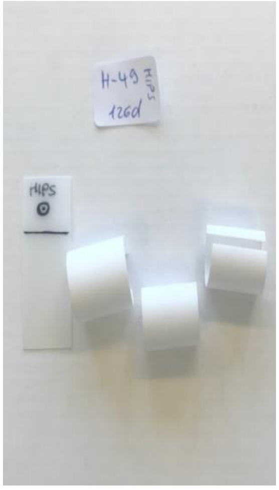	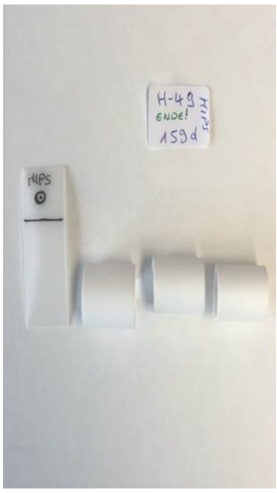

8.3 Isooctane at 20 °C

<table>
<tr>
<td>2 d
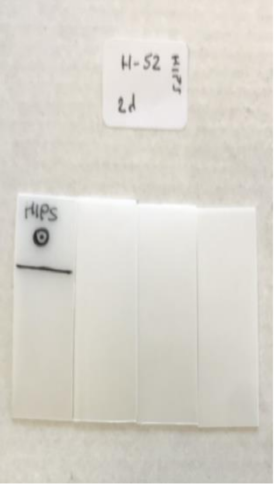</td>
<td>5 d
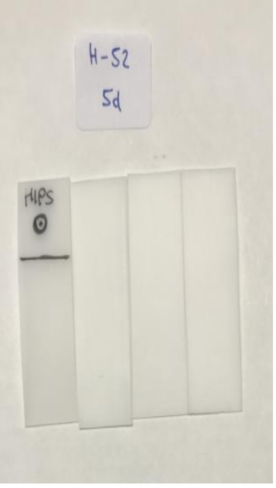</td>
<td>12 d
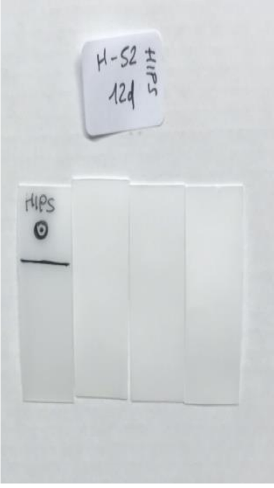</td>
</tr>
<tr>
<td>16 d
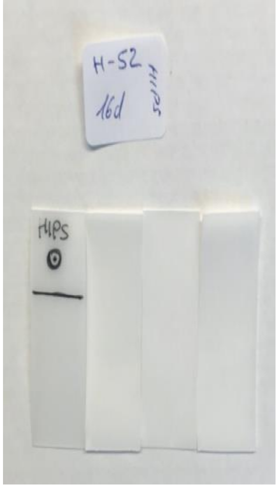</td>
<td>30 d
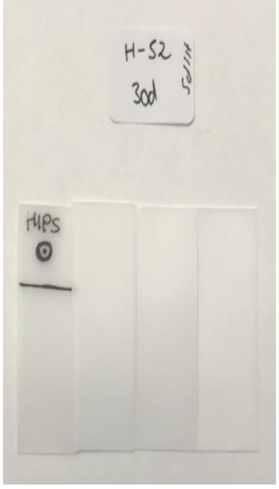</td>
<td>44 d
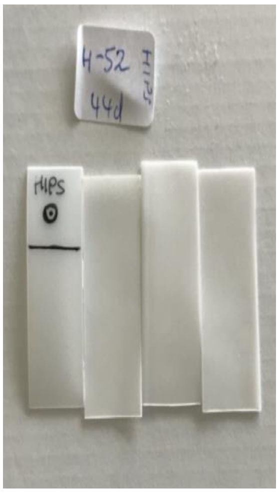</td>
</tr>
<tr>
<td>64 d
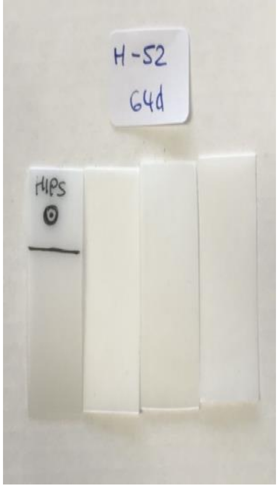</td>
<td>92 d
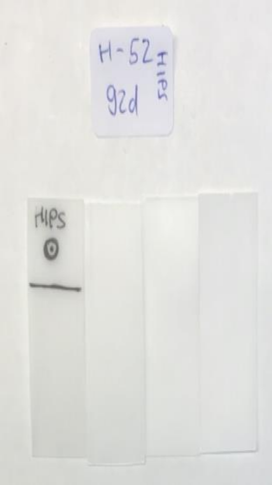</td>
<td>126 d
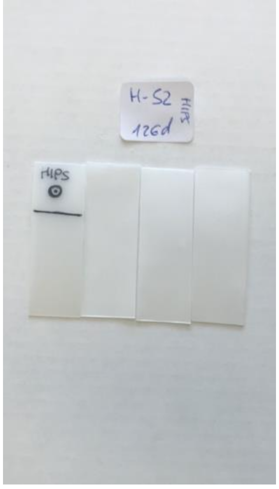</td>
</tr>
</table>

159 d	201 d	273 d
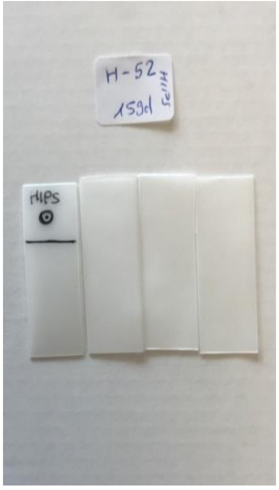	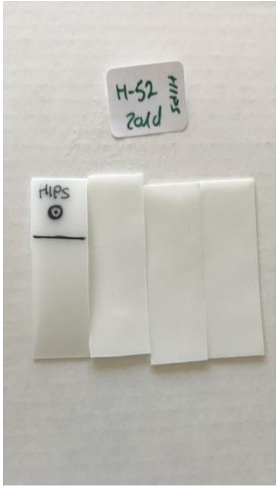	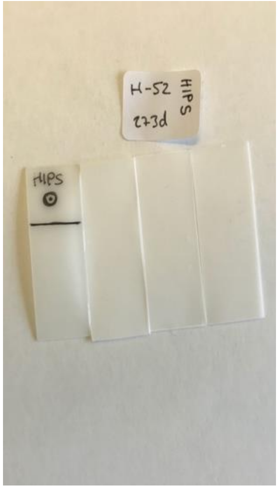

9 Contact with 95% Ethanol

9.1 95% Ethanol at 60 °C

<table>
<tr>
<td>4 h
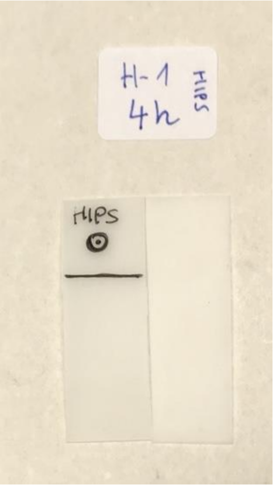
</td>
<td>8 h
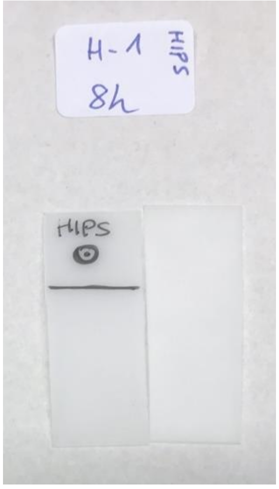
</td>
<td>1 d
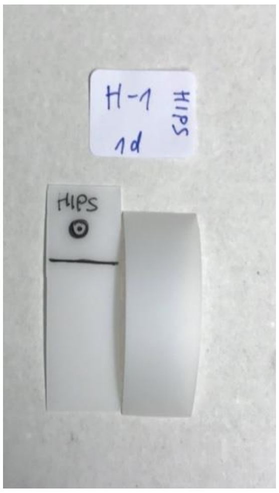
</td>
</tr>
<tr>
<td>2 d
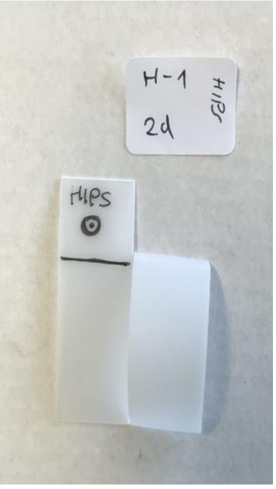
</td>
<td>5 d
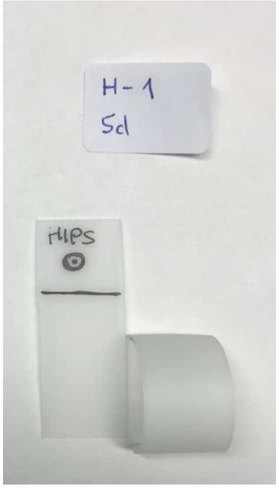
</td>
<td>12 d
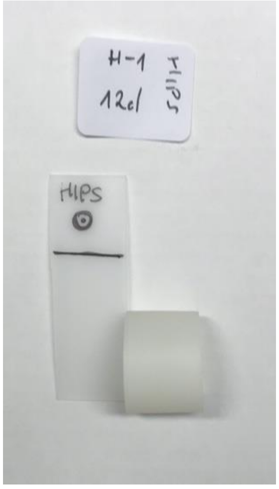
</td>
</tr>
</table>

16 d 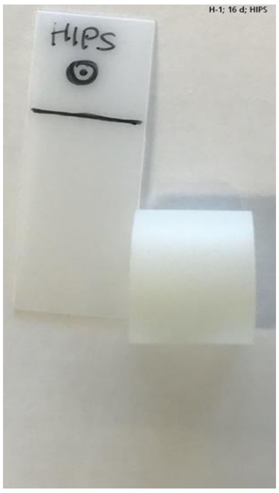	30 d 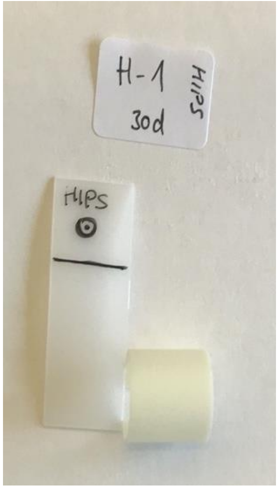	44 d 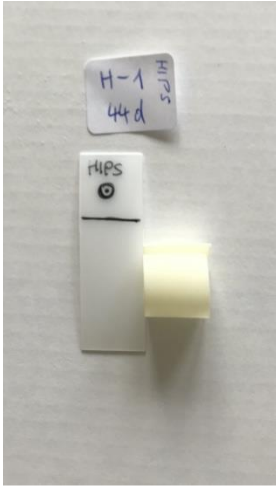
64 d 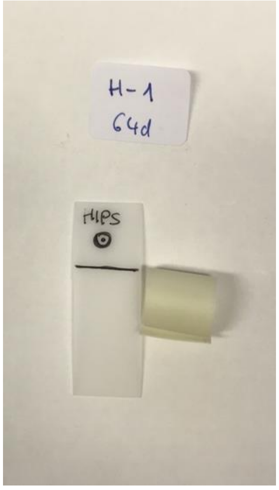	92 d 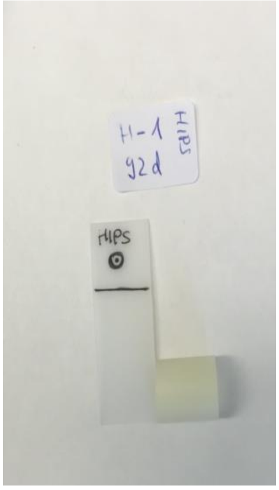	126 d 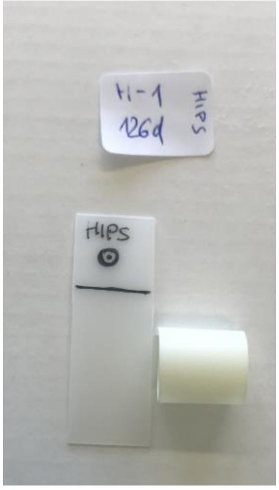
159 d 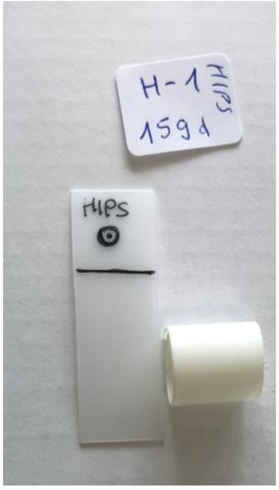	201 d 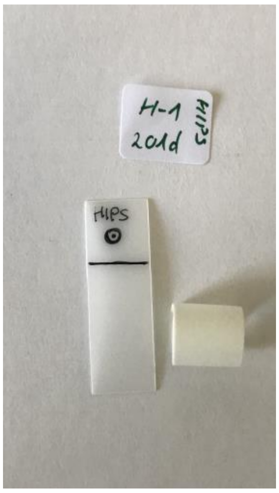	273 d 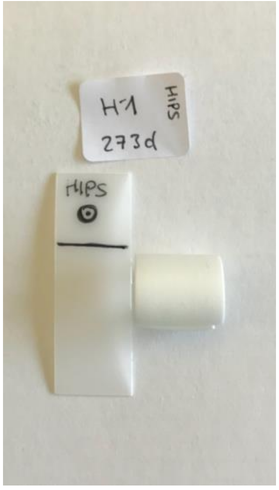

9.2 95% Ethanol at 40 °C

4 h 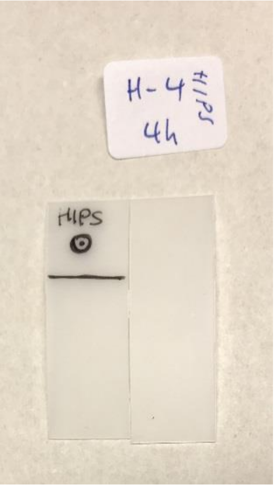	8 h 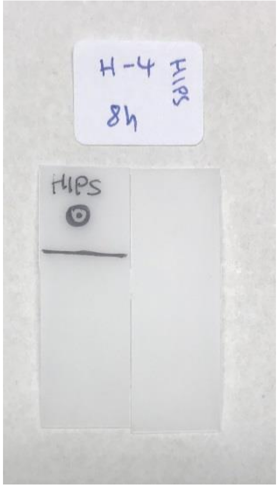	1 d 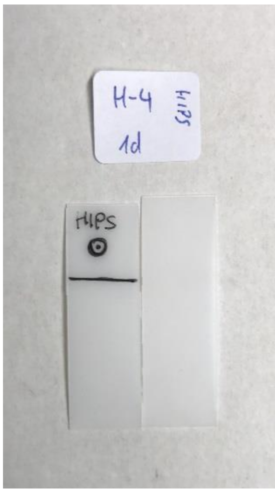
2 d 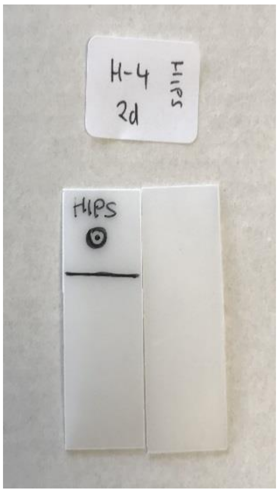	5 d 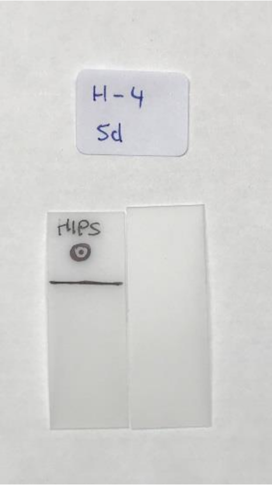	12 d 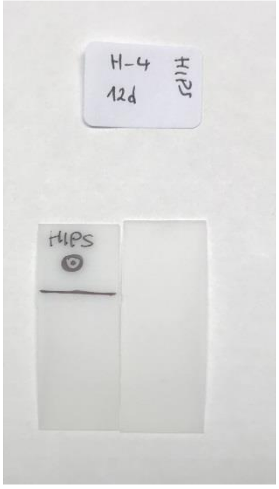
16 d 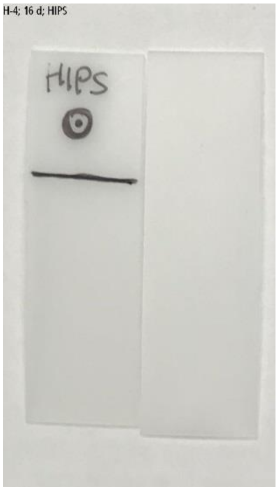	30 d 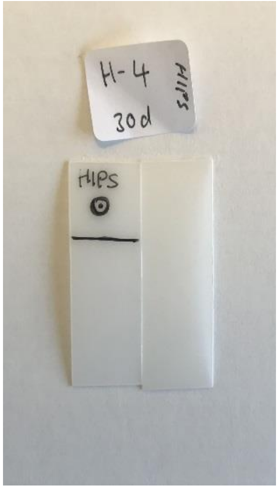	44 d 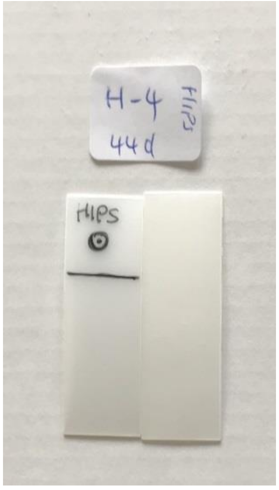

<table>
<tr>
<td>64 d
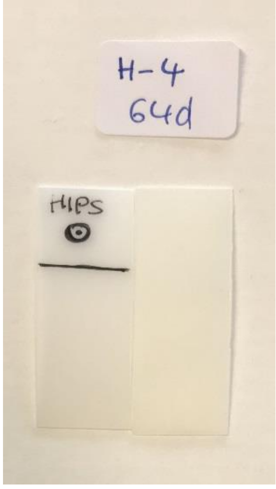
</td>
<td>92 d
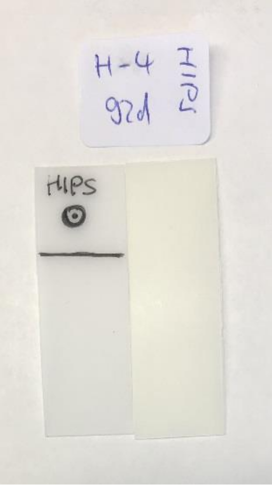
</td>
<td>126 d
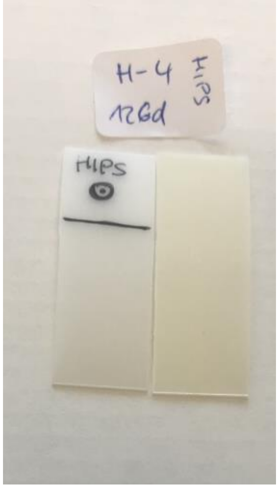
</td>
</tr>
<tr>
<td>159 d
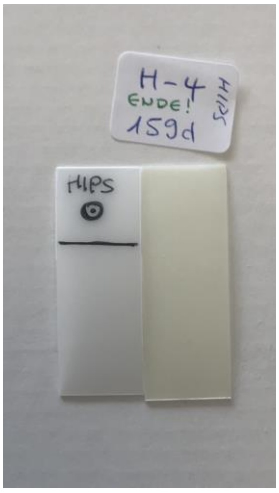
</td>
<td></td>
<td></td>
</tr>
</table>

9.3 95% Ethanol at 20 °C

<table>
<tr>
<td>2 d
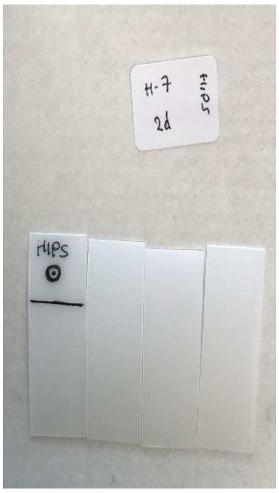
</td>
<td>5 d
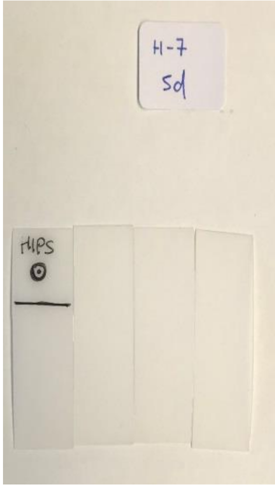
</td>
<td>12 d
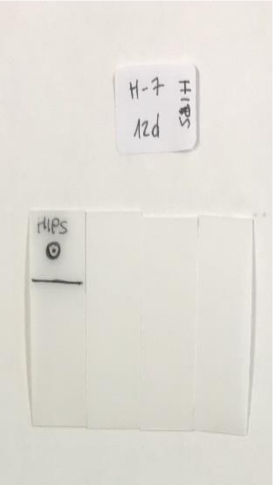
</td>
</tr>
<tr>
<td>16 d
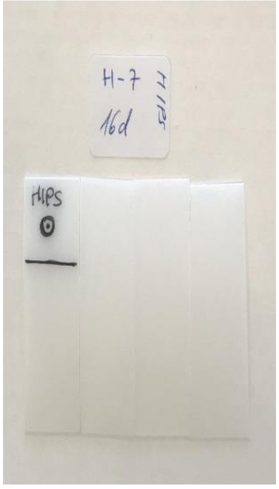
</td>
<td>30 d
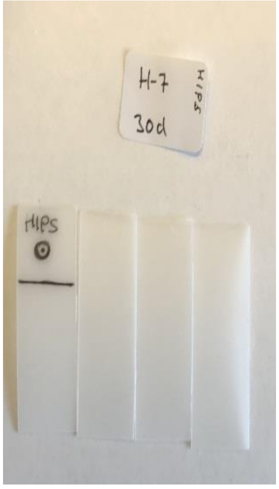
</td>
<td>44 d
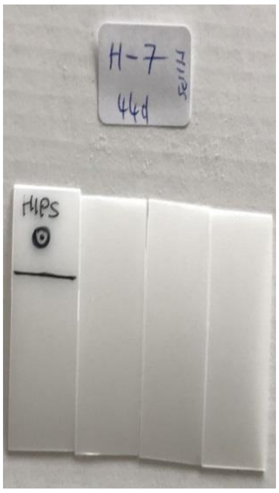
</td>
</tr>
<tr>
<td>64 d
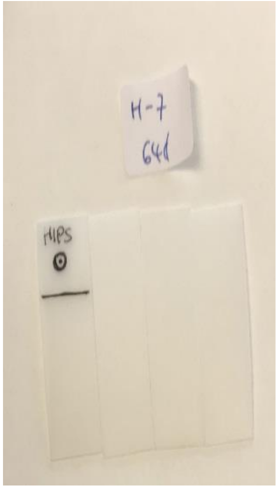
</td>
<td>92 d
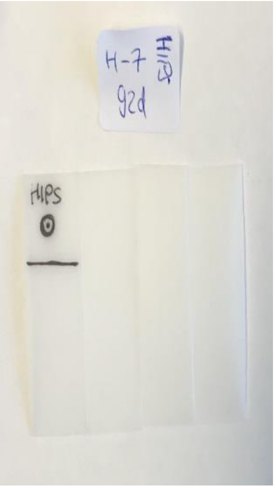
</td>
<td>126 d
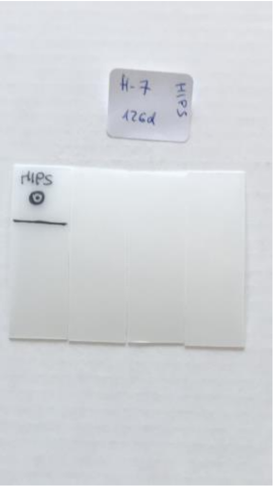
</td>
</tr>
</table>

159 d 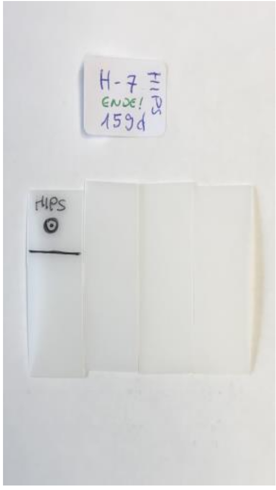		

10 Contact with 50% Ethanol

10.1 50% Ethanol at 60 °C

<table>
<tr>
<td>4 h
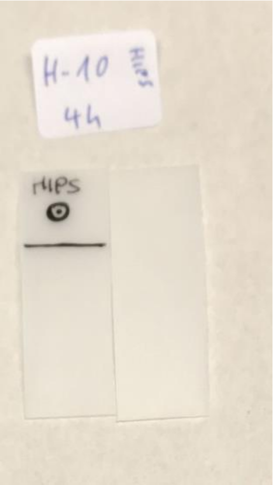</td>
<td>8 h
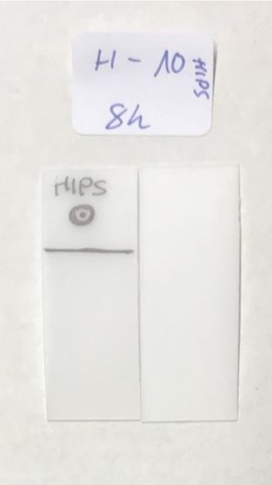</td>
<td>1 d
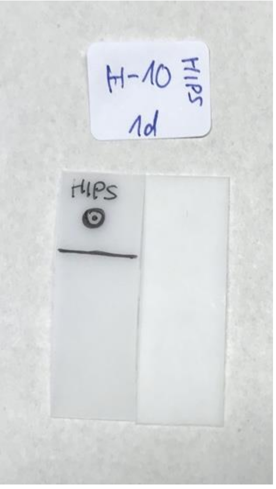</td>
</tr>
<tr>
<td>2 d
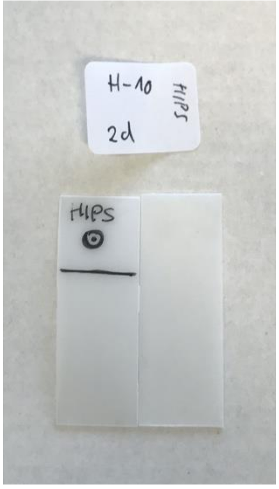</td>
<td>5 d
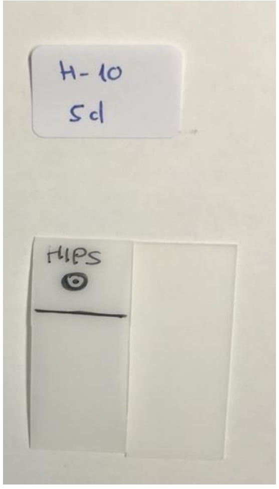</td>
<td>12 d
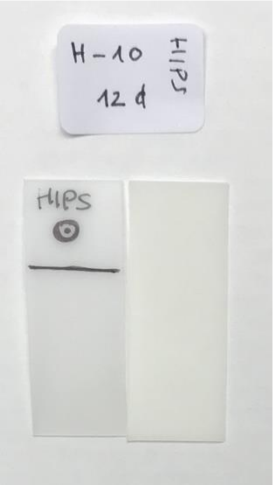</td>
</tr>
</table>

16 d

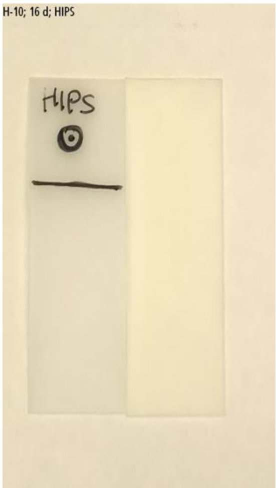

30 d

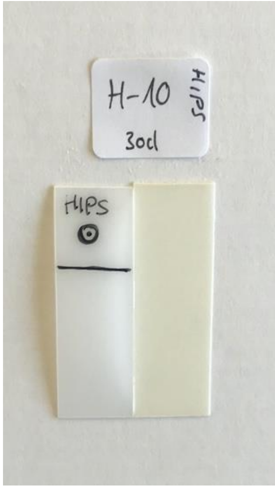

44 d

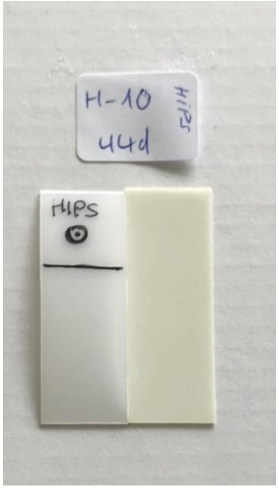

64 d

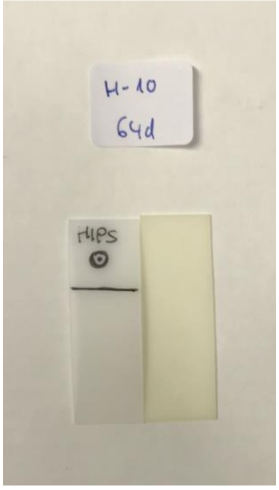

92 d

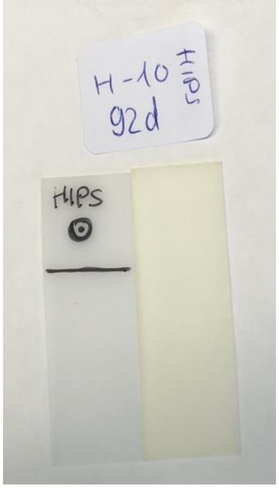

126 d

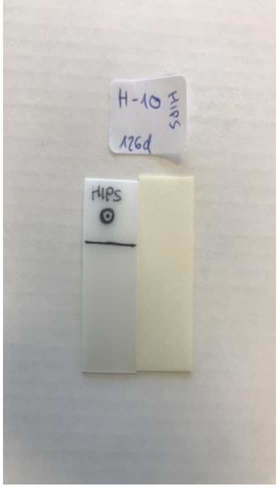

159 d

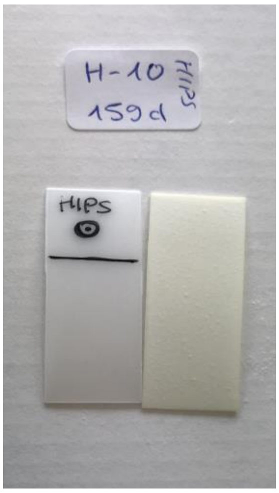

201 d

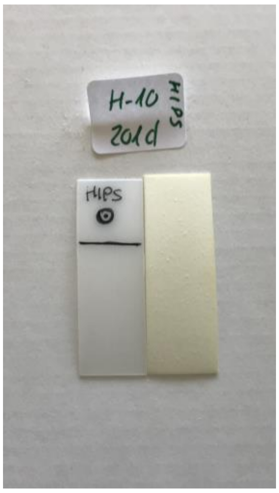

273 d

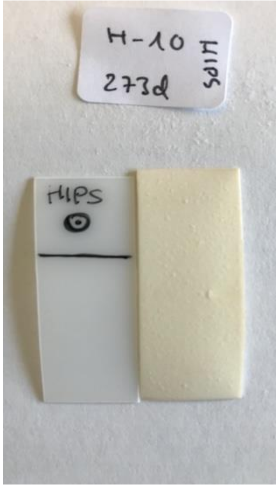

10.2 50% Ethanol at 40 °C

<table>
<tr>
<td>1 d
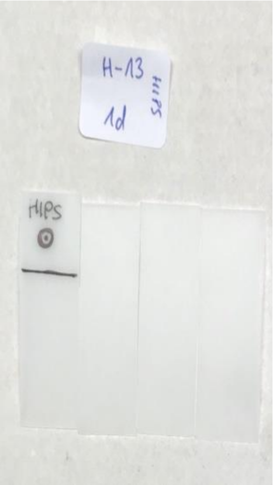</td>
<td>2 d
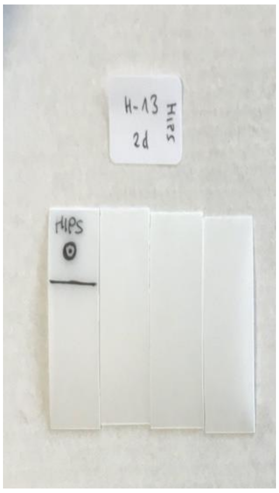</td>
<td>5 d
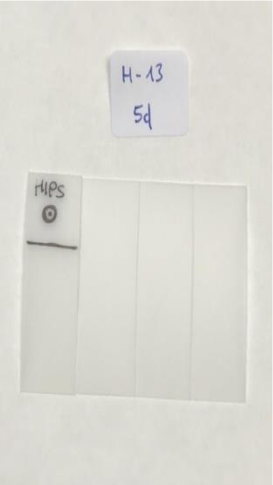</td>
</tr>
<tr>
<td>12 d
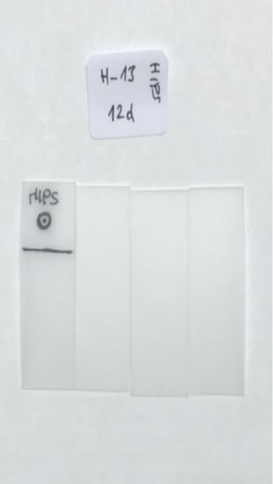</td>
<td>16 d
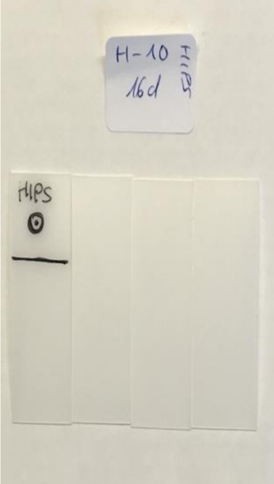</td>
<td>30 d
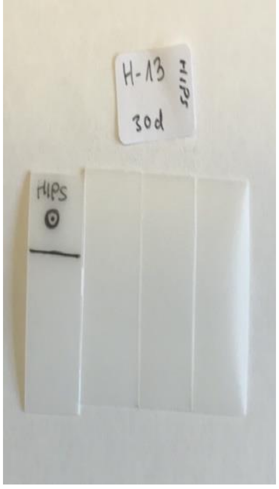</td>
</tr>
<tr>
<td>44 d
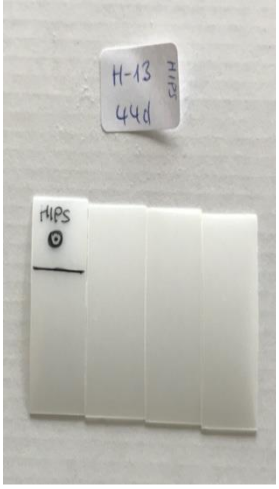</td>
<td>64 d
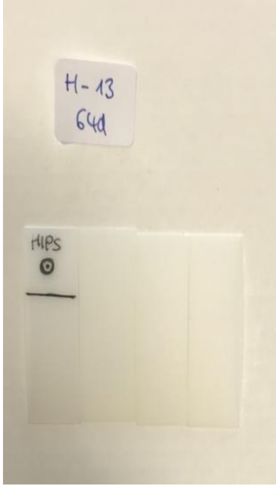</td>
<td>92 d
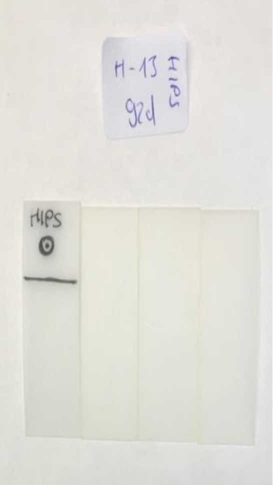</td>
</tr>
</table>

126 d	159 d	
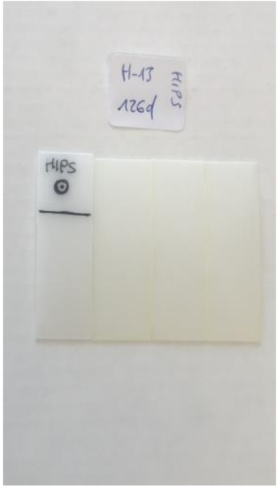	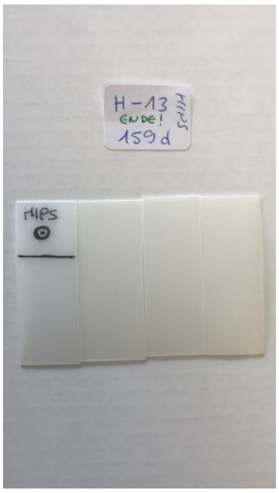	

10.3 50% Ethanol at 20 °C

<table>
<tr>
<td>2 d
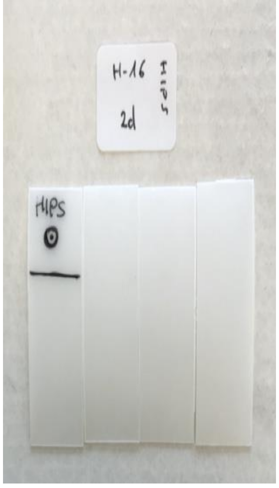
</td>
<td>5 d
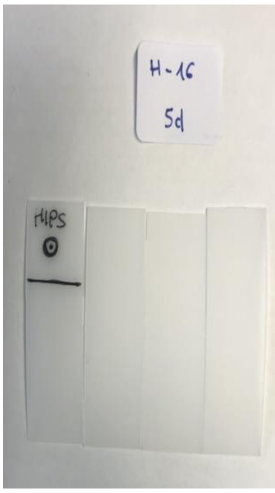
</td>
<td>12 d
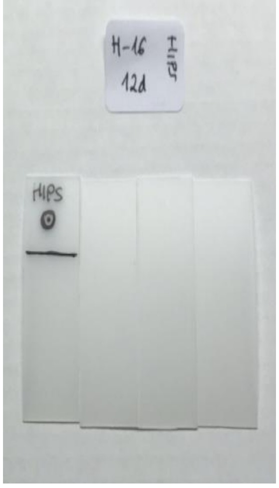
</td>
</tr>
<tr>
<td>16 d
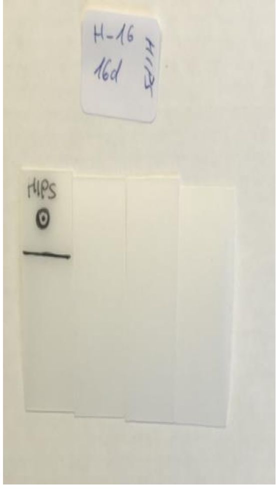
</td>
<td>30 d
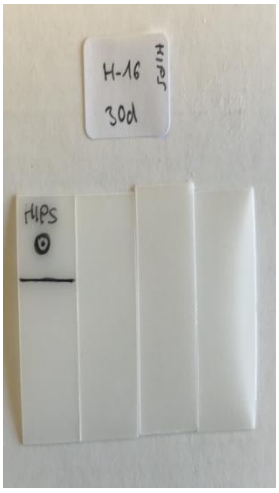
</td>
<td>44 d
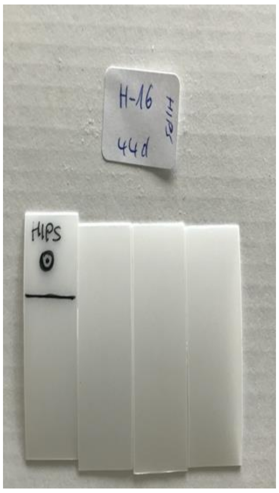
</td>
</tr>
<tr>
<td>64 d
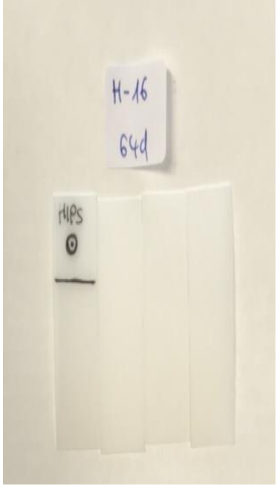
</td>
<td>92 d
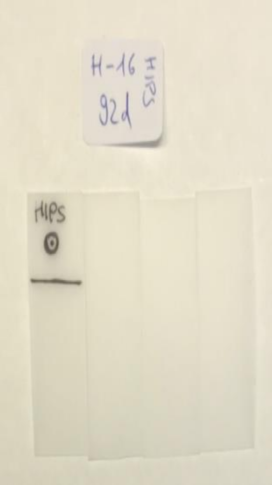
</td>
<td>126 d
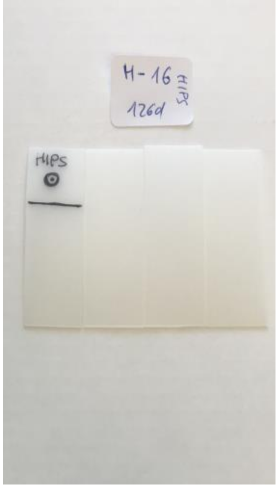
</td>
</tr>
</table>

<table>
<tr><td>159 d
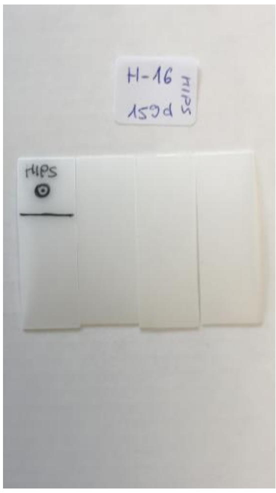
</td><td>201 d
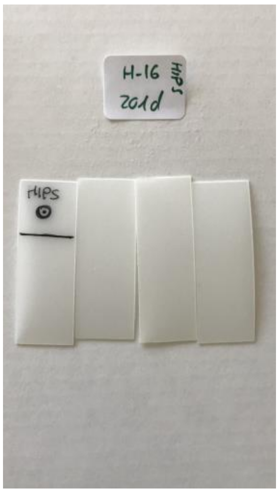
</td><td>273 d
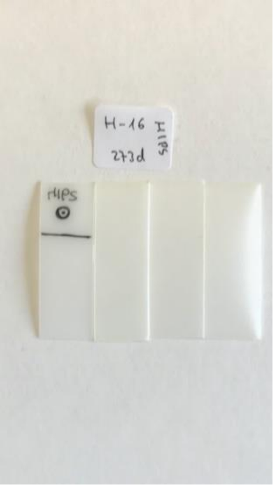
</td></tr>
</table>

11 Contact with 20% Ethanol

11.1 20% Ethanol at 60 °C

2 d

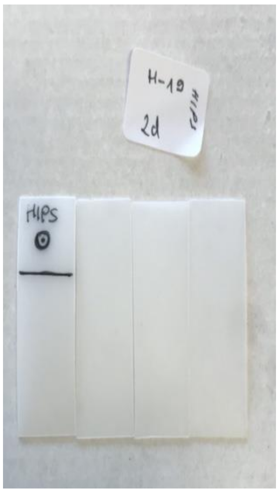

5 d

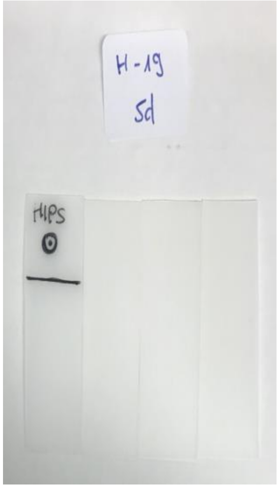

12 d

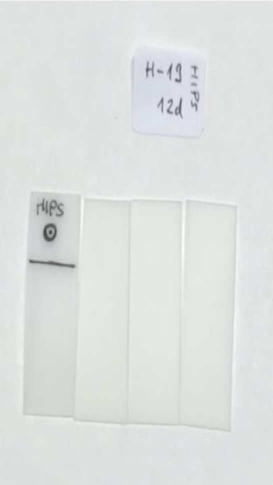

16 d

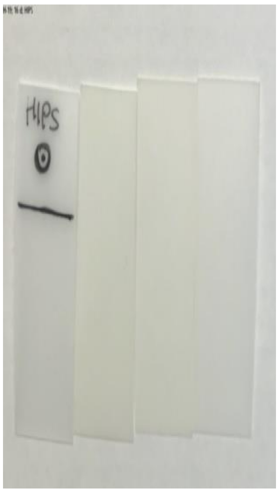

30 d

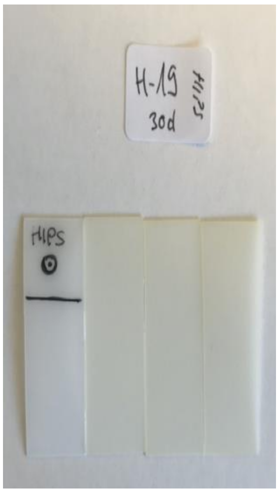

44 d

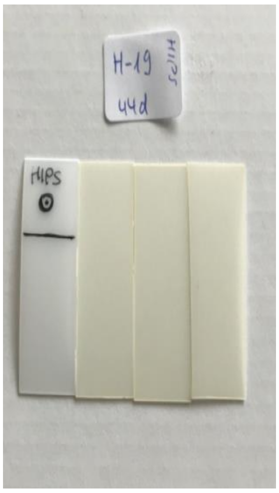

64 d

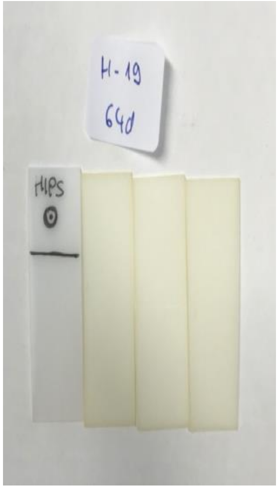

92 d

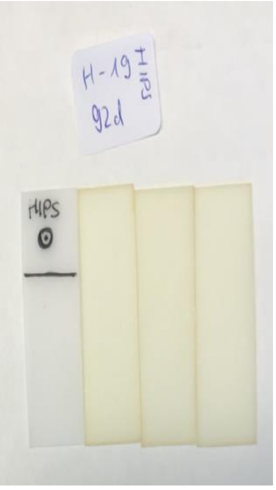

126 d

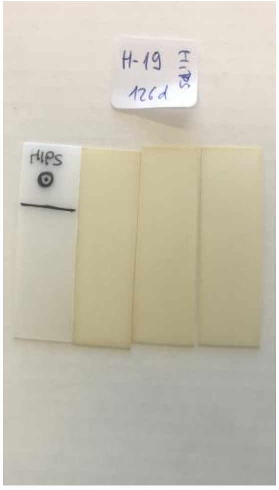

159 d

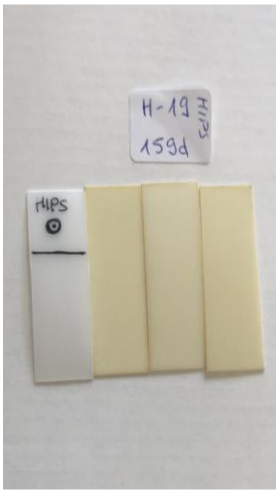

201 d

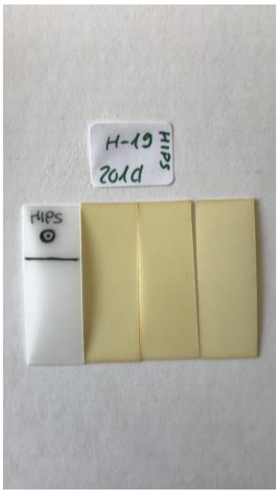

273 d

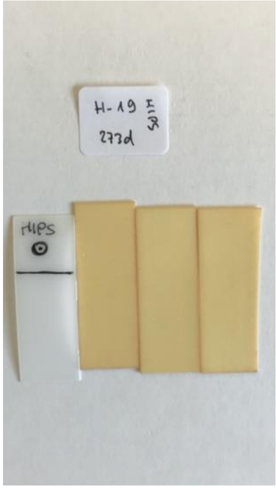

11.2 20% Ethanol at 40 °C

2 d 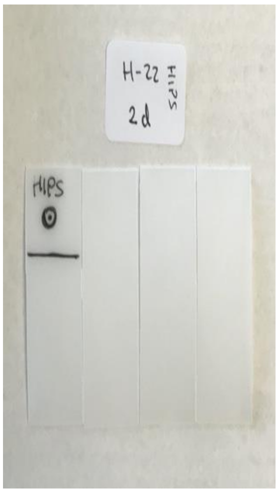	5 d 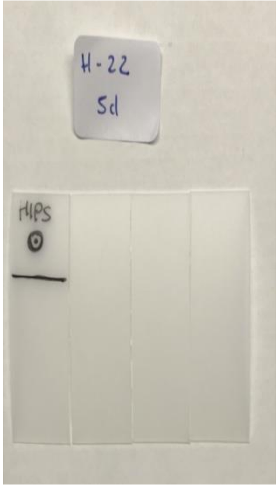	12 d 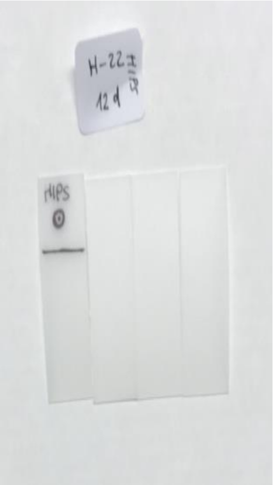
16 d 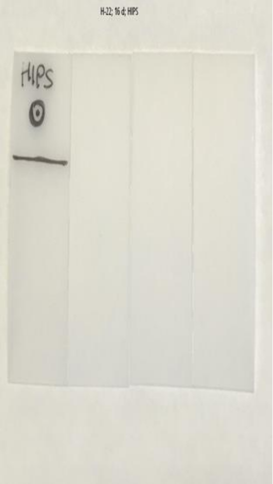	30 d 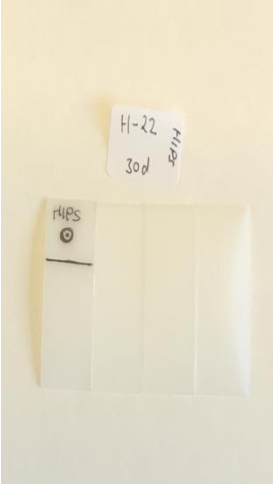	44 d 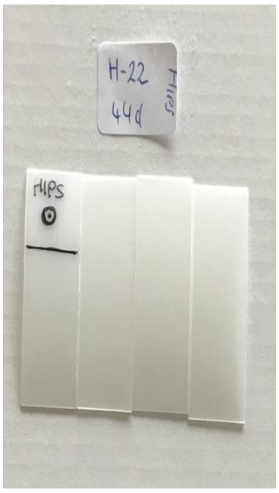
64 d 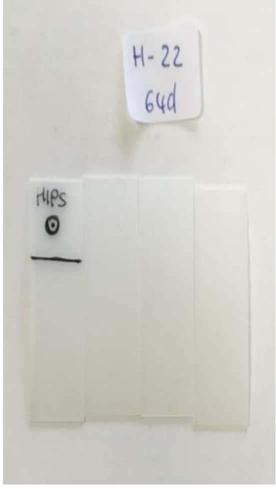	92 d 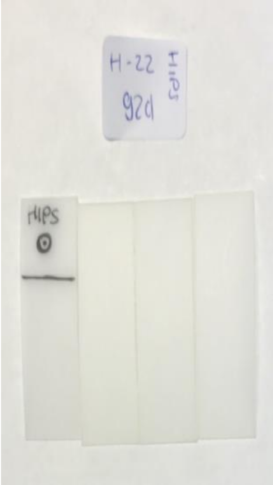	126 d 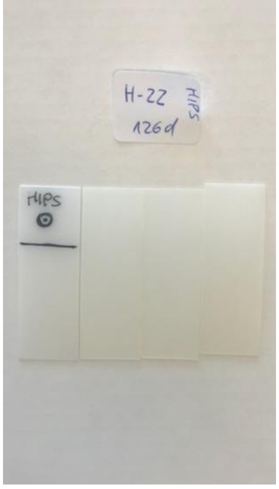

<table>
<tr>
<td>159 d
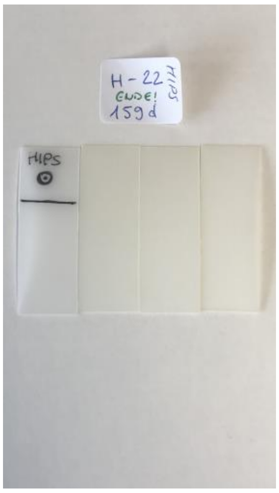
</td>
<td></td>
<td></td>
</tr>
</table>

11.3 20% Ethanol at 20 °C

2 d

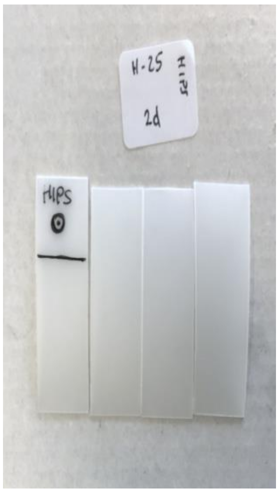

5 d

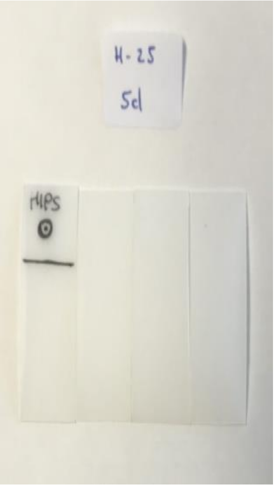

12 d

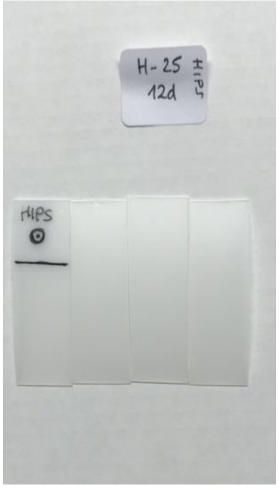

16 d

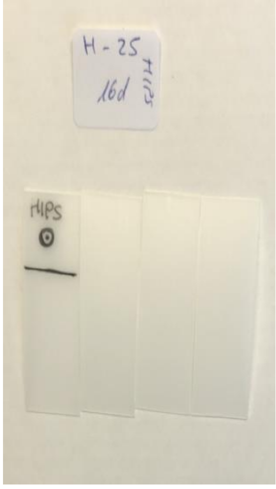

30 d

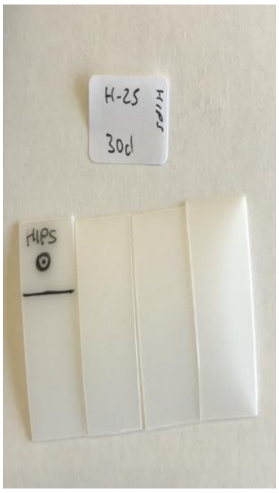

44 d

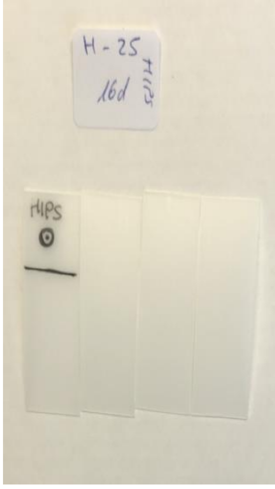

64 d

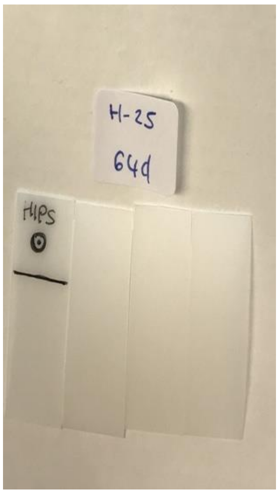

92 d

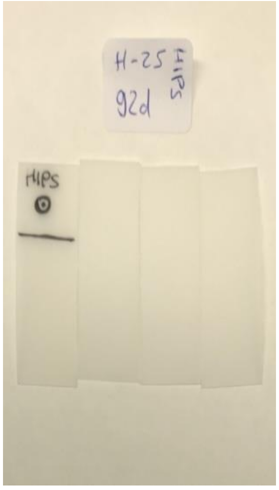

126 d

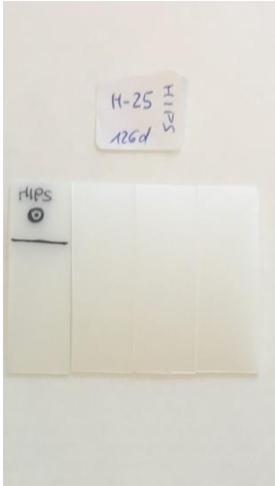

159 d 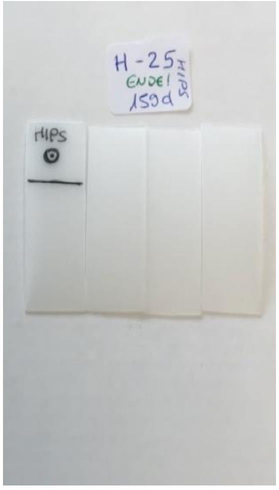		

12 Contact with 10% Ethanol

12.1 10% Ethanol at 60 °C

2 d 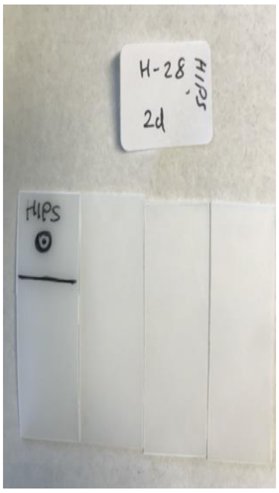	5 d 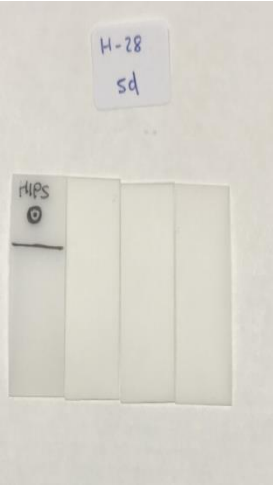	12 d 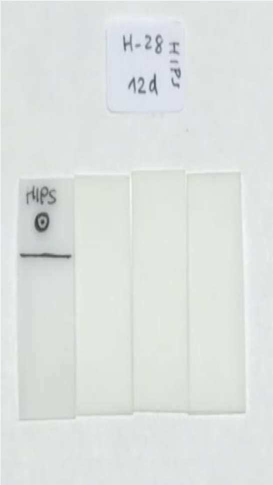
16 d 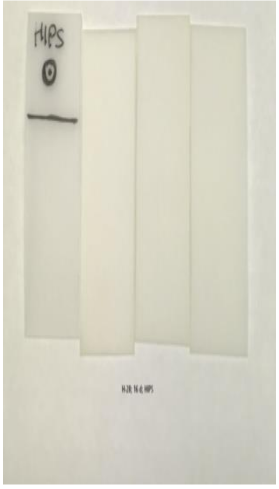	30 d 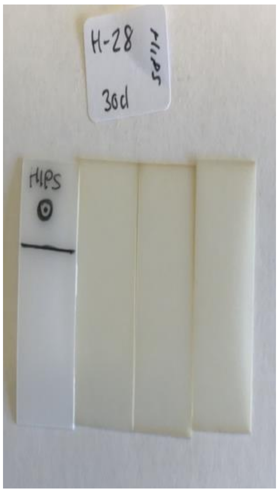	44 d 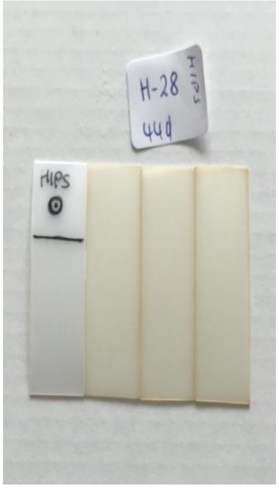

<table>
<tr>
<td>64 d
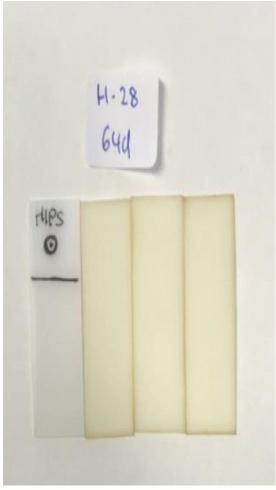</td>
<td>92 d
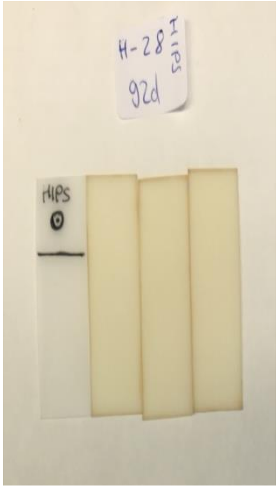</td>
<td>126 d
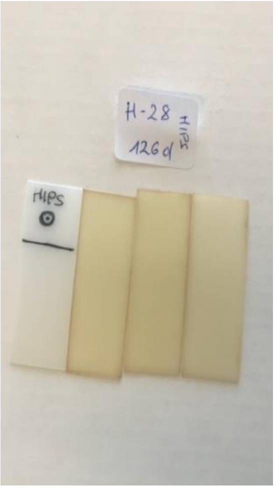</td>
</tr>
<tr>
<td>159 d
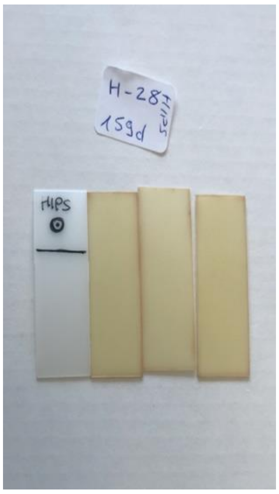</td>
<td>201 d
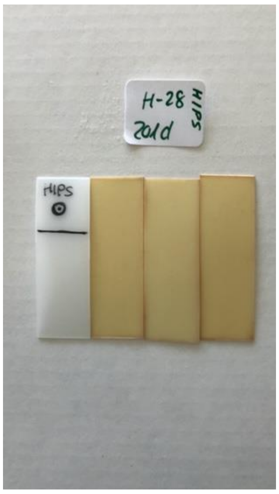</td>
<td>273 d
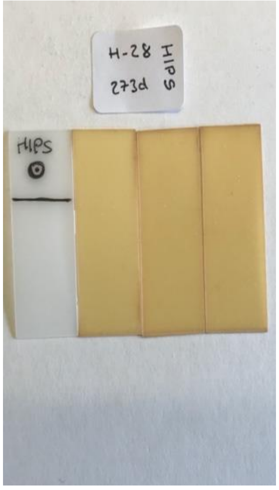</td>
</tr>
</table>

12.2 10% Ethanol at 40 °C

<table>
<tr>
<td>2 d
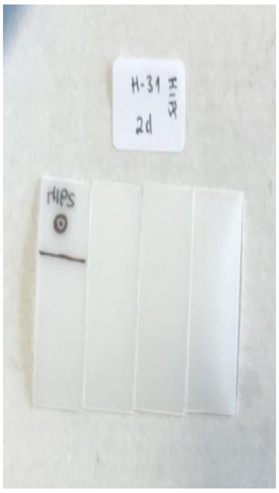
</td>
<td>5 d
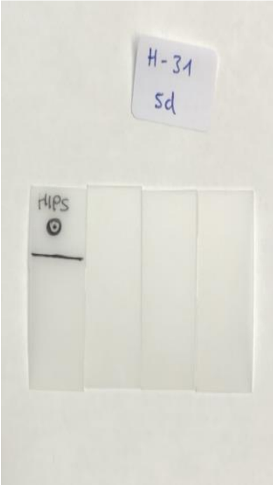
</td>
<td>12 d
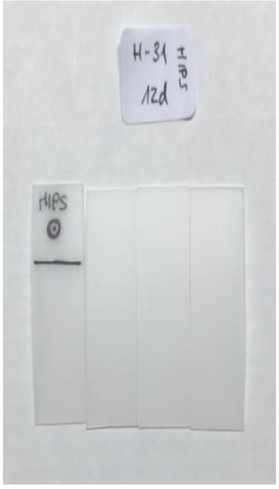
</td>
</tr>
<tr>
<td>16 d
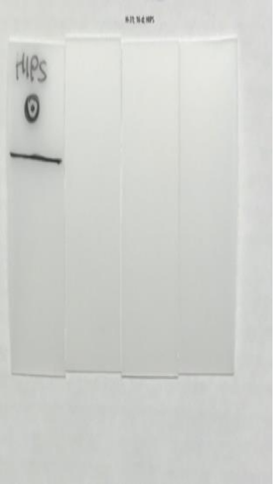
</td>
<td>30 d
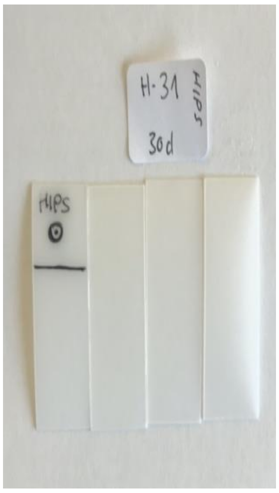
</td>
<td>44 d
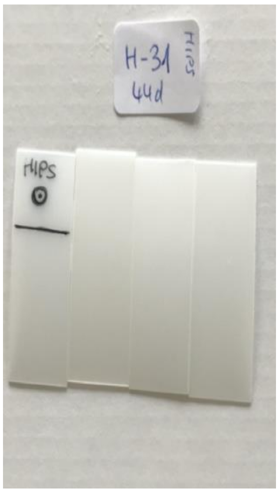
</td>
</tr>
<tr>
<td>64 d
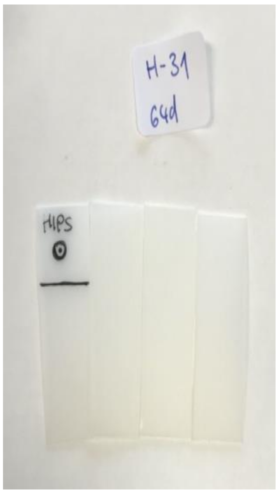
</td>
<td>92 d
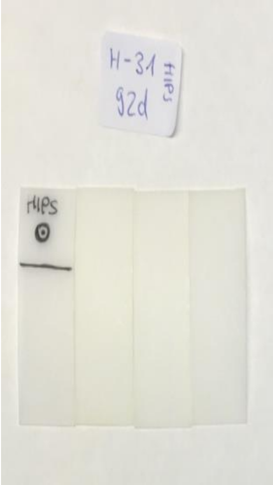
</td>
<td>126 d
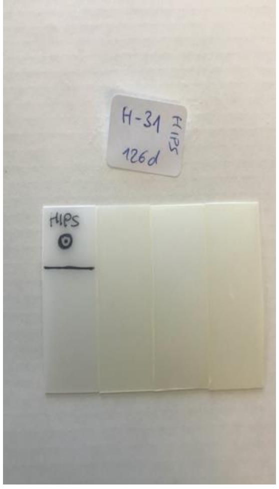
</td>
</tr>
</table>

159 d 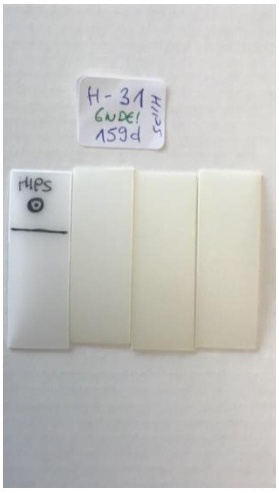		

12.3 10% Ethanol at 20 °C

<table>
<tr>
<td>2 d
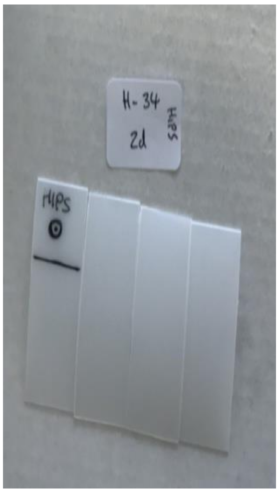</td>
<td>5 d
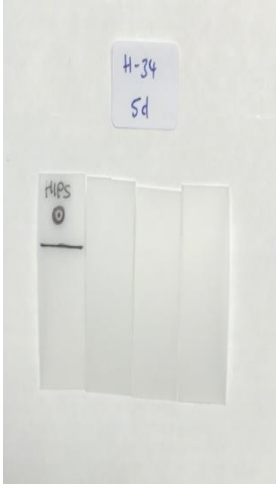</td>
<td>12 d
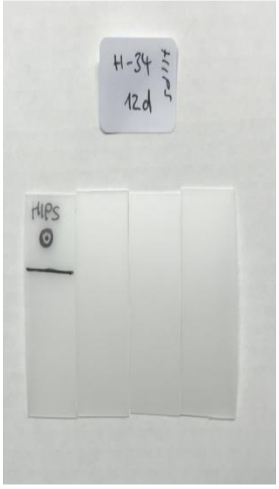</td>
</tr>
<tr>
<td>16 d
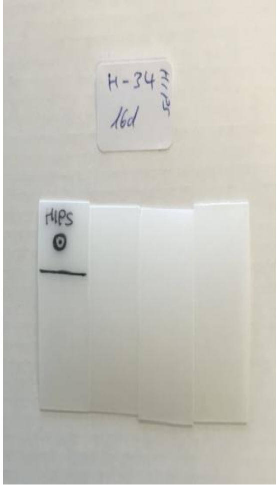</td>
<td>30 d
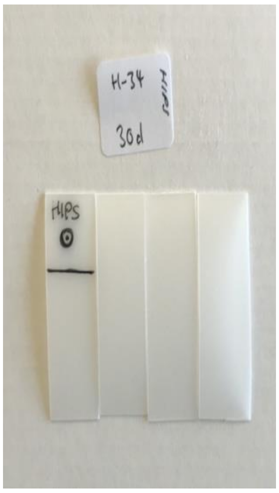</td>
<td>44 d
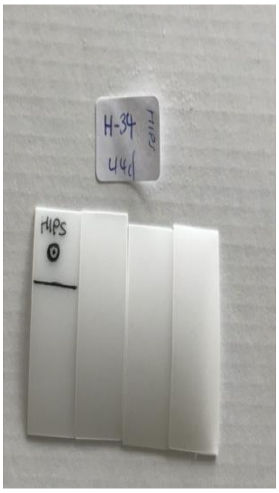</td>
</tr>
<tr>
<td>64 d
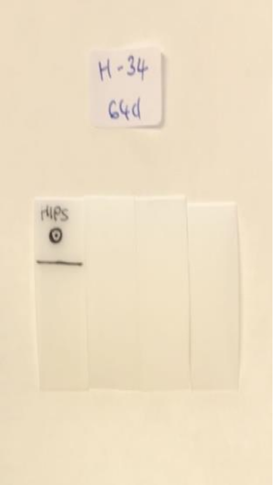</td>
<td>92 d
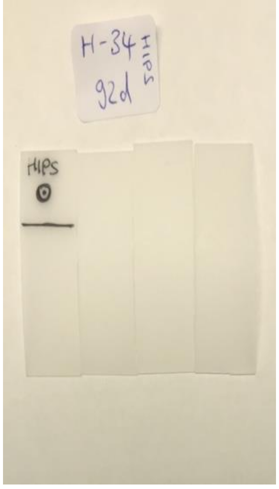</td>
<td>126 d
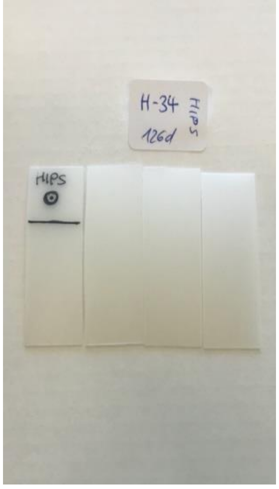</td>
</tr>
</table>

159 d 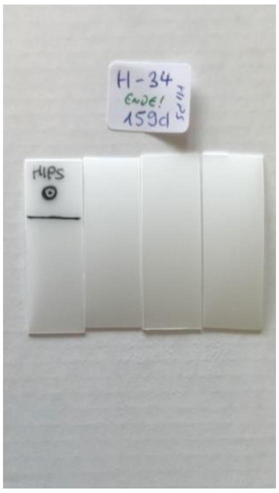		

13 Contact with 3% acetic acid

13.1 3% Acetic acid at 60 °C

2 d 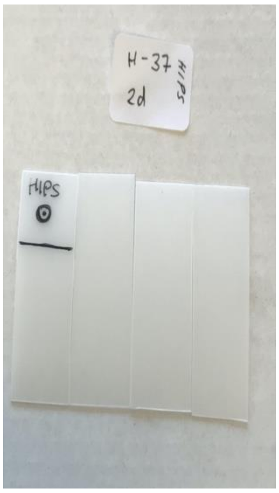	5 d 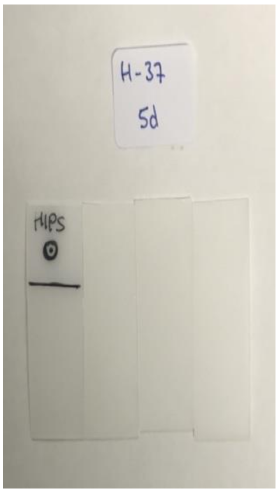	12 d 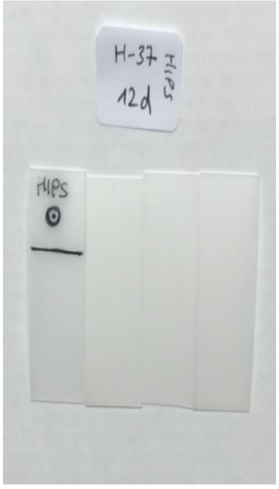
16 d 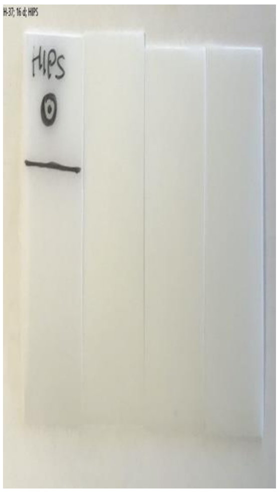	30 d 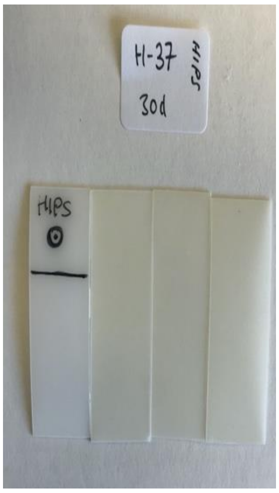	44 d 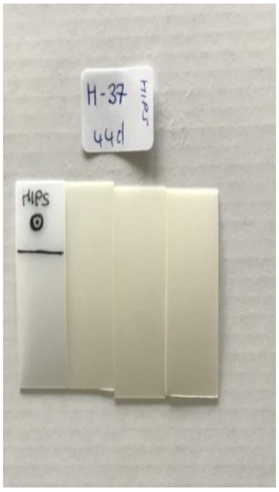

64 d

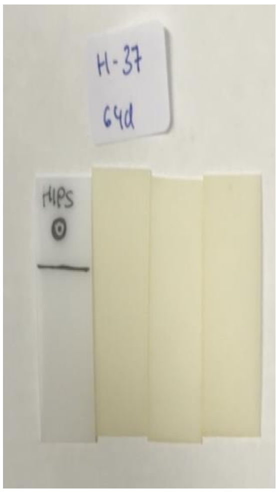

92 d

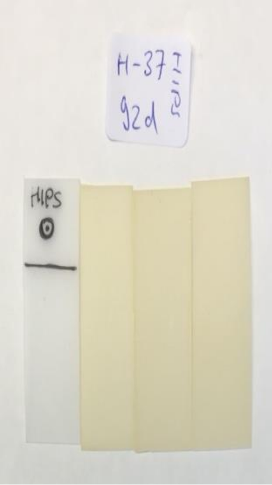

126 d

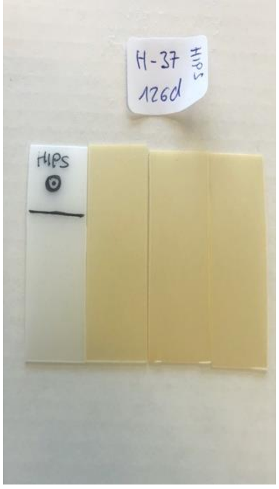

159 d

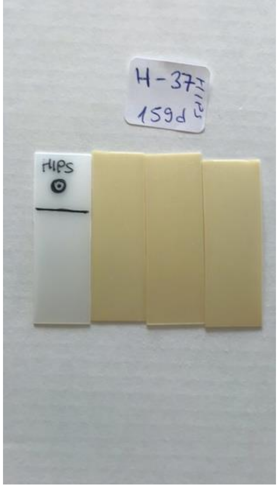

201 d

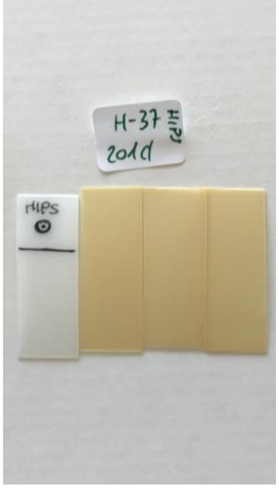

273 d

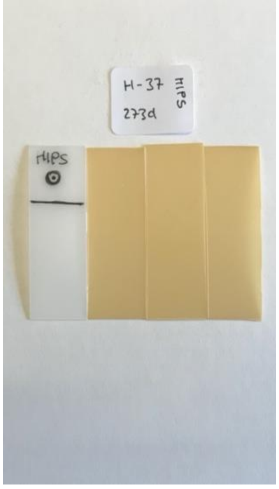

13.2 3% Acetic acid at 40 °C

<table>
<tr>
<td>2 d
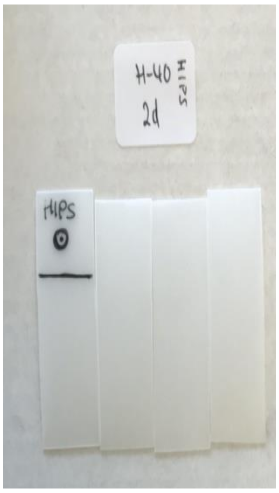</td>
<td>5 d
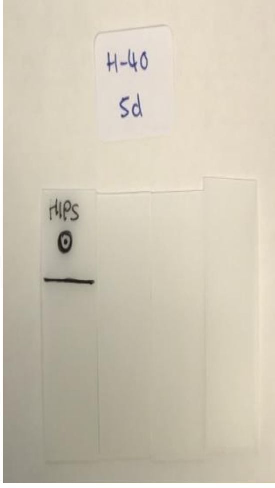</td>
<td>12 d
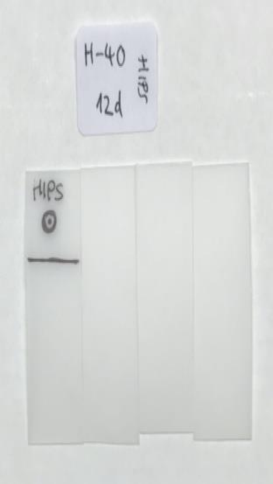</td>
</tr>
<tr>
<td>16 d
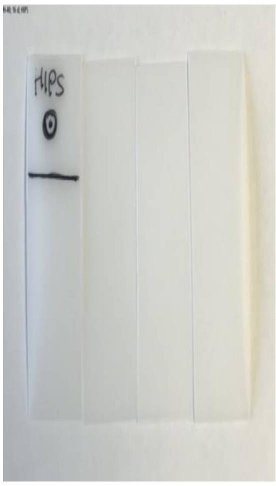</td>
<td>30 d
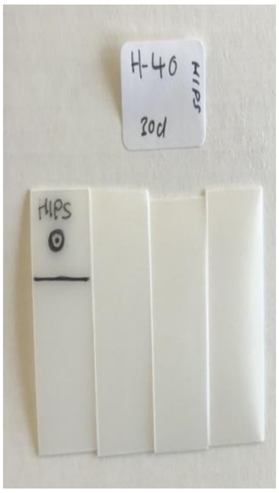</td>
<td>44 d
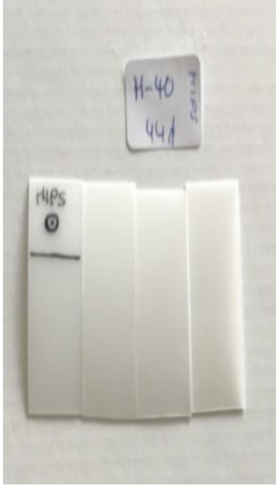</td>
</tr>
<tr>
<td>64 d
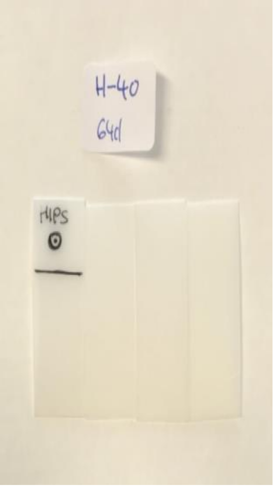</td>
<td>92 d
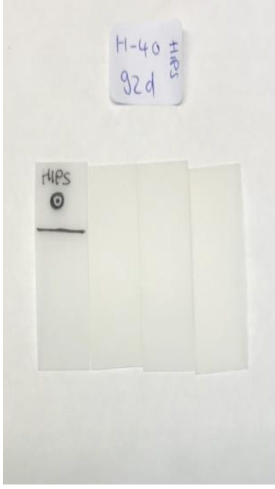</td>
<td>126 d
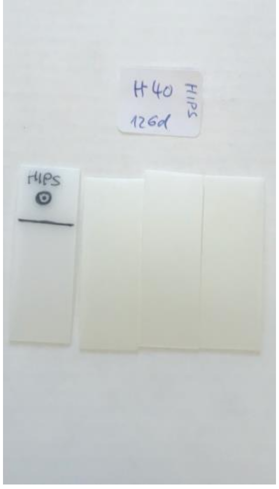</td>
</tr>
</table>

159 d

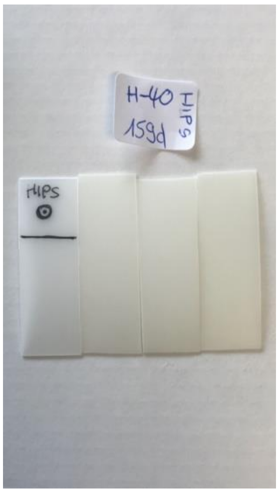

201 d

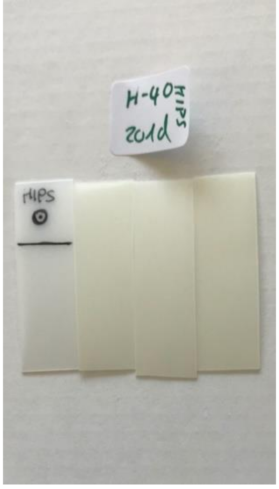

273 d

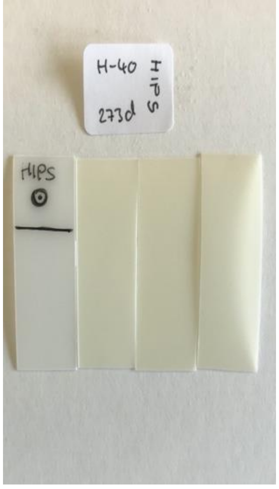

13.3 3% Acetic acid at 20 °C

2 d

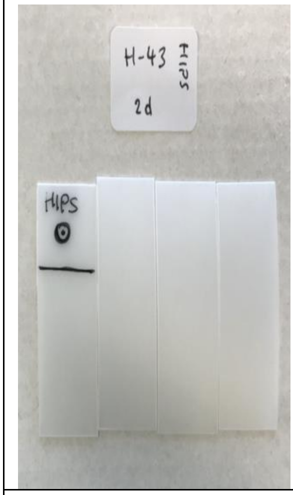

5 d

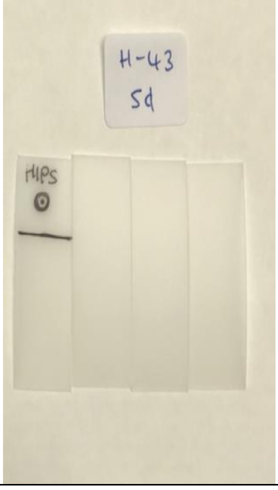

12 d

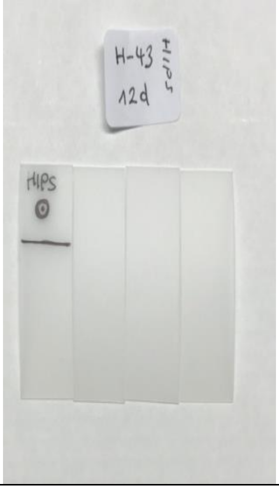

16 d

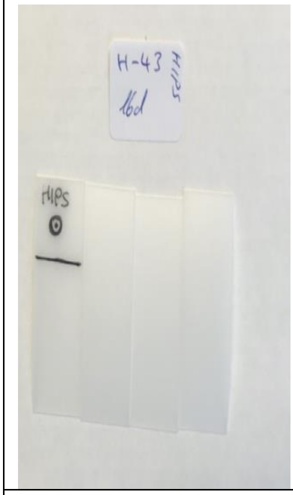

30 d

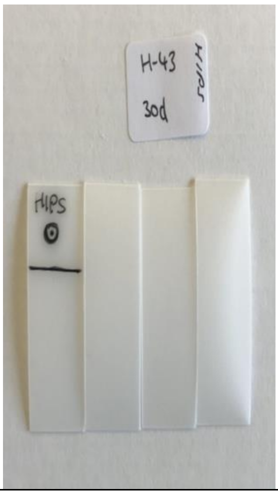

44 d

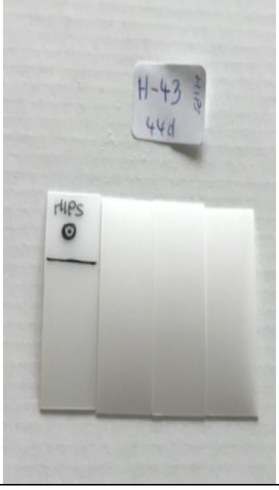

64 d

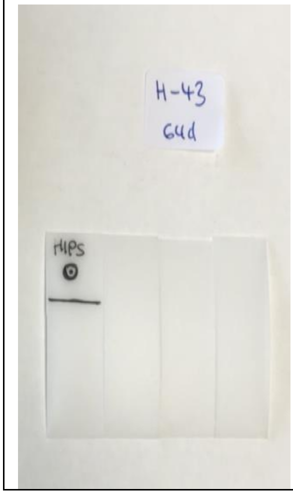

92 d

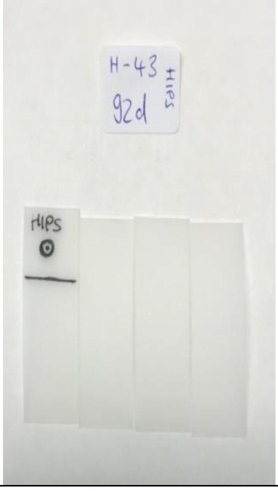

126 d

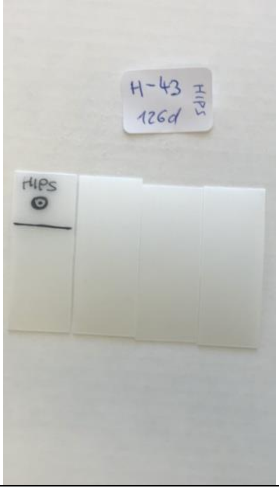

159 d 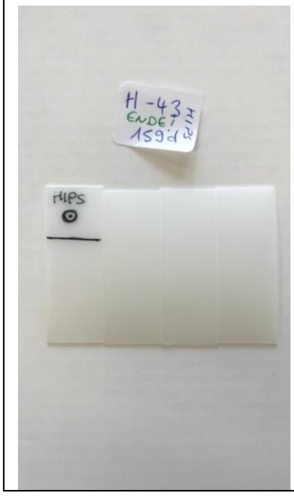		

14 Contact with real foods

14.1 Milk, cream and olive oil 60 d at 20 °C

Milk	Cream	Olive oil
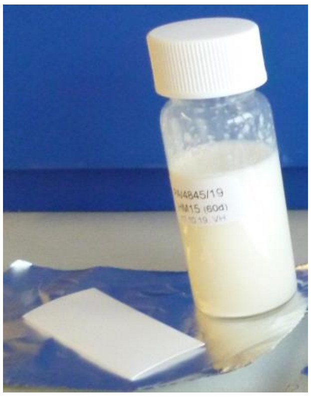	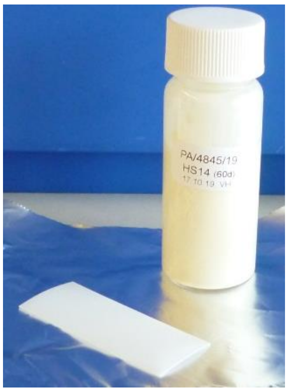	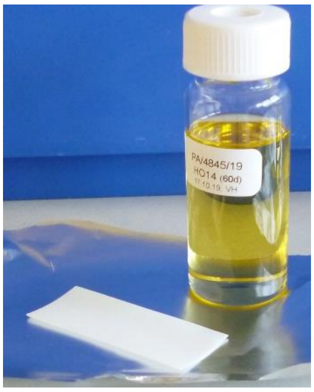

14.2 Milk, cream and olive oil 10 d at 40 °C

Milk	Cream	Olive oil
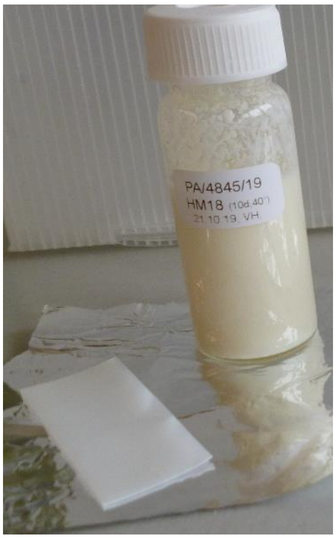	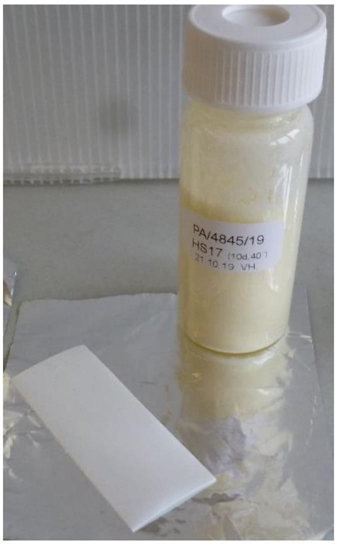	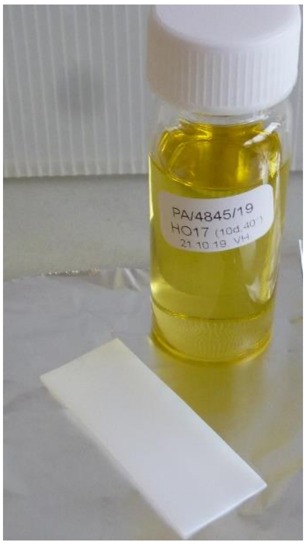

14.3 Lard at 5 °C

<table>
<tr><th>10 d</th><th>20 d</th><th>30 d</th></tr>
<tr>
<td>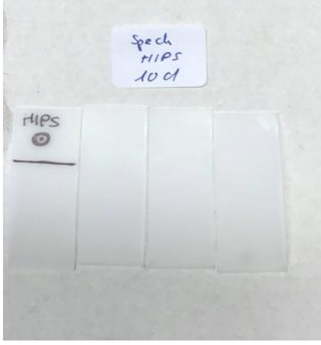
</td>
<td>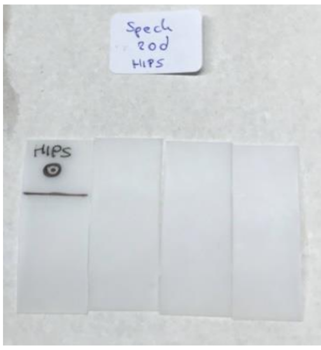
</td>
<td>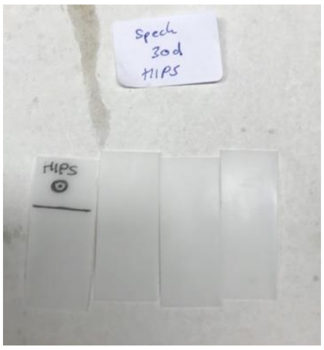
</td>
</tr>
</table>

14.4 Butter at 5 °C

<table>
<tr><th>10 d</th><th>20 d</th><th>30 d</th></tr>
<tr>
<td>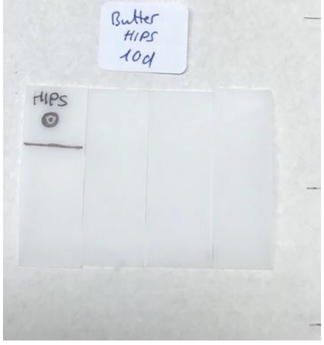
</td>
<td>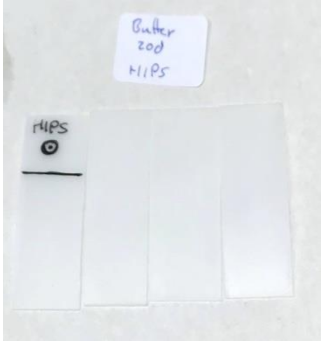
</td>
<td>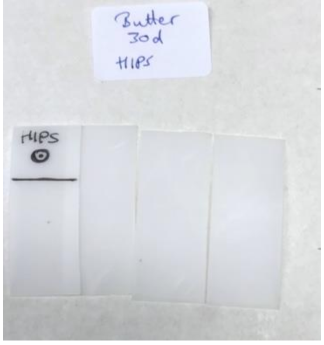
</td>
</tr>
</table>

14.5 Fish oil at 20 °C

<table>
<tr><th>10 d</th><th>20 d</th><th>30 d</th></tr>
<tr>
<td>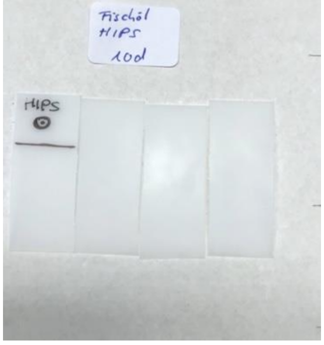
</td>
<td>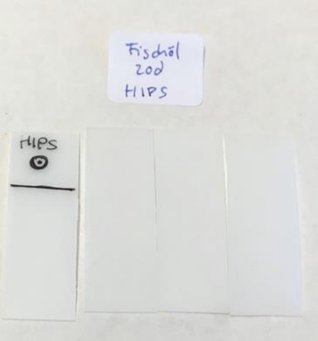
</td>
<td>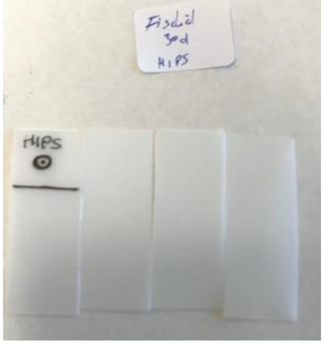
</td>
</tr>
</table>

14.6 Miglyol® 812 at 20°C

10 d (1)	20 d (1)	30 d
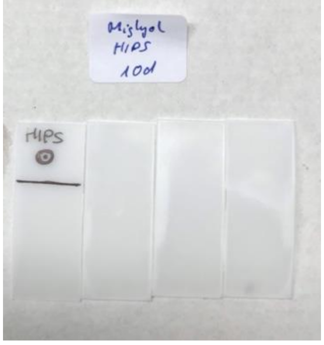	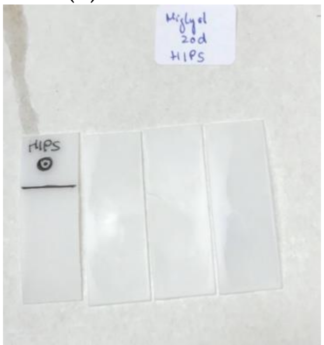	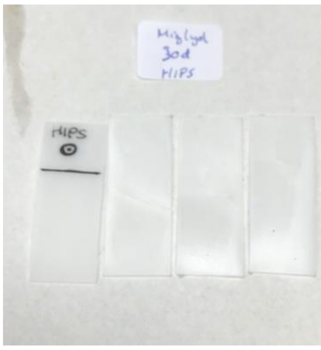
10 d (2)	20 d (2)	30 (2)
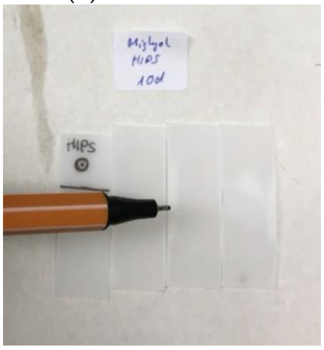	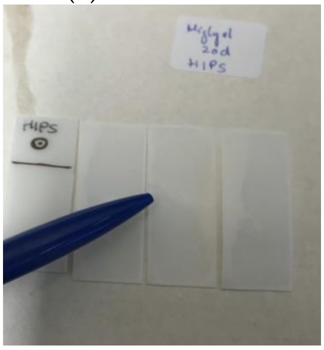	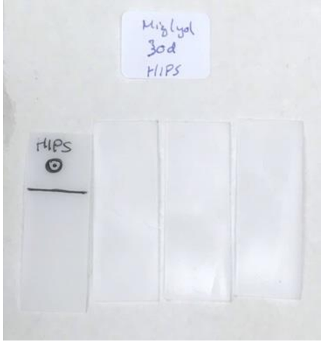

Note: for Miglyol, photos for two replicates (1) and (2) are reported

14.7 Clear orange juice at 20 °C

10 d	20 d	30 d
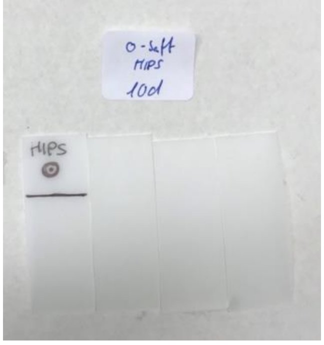	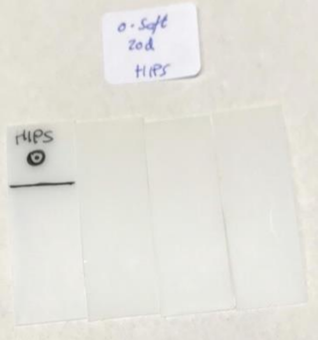	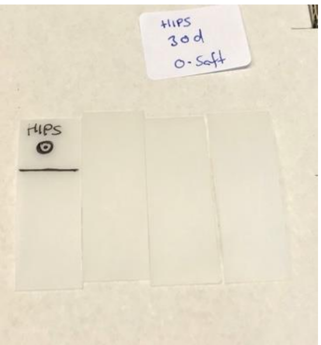

14.8 Ground coffee beans at 20 °C

10 d	20 d	30 d
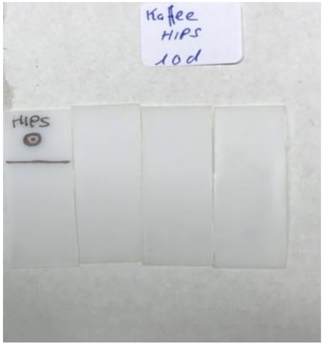	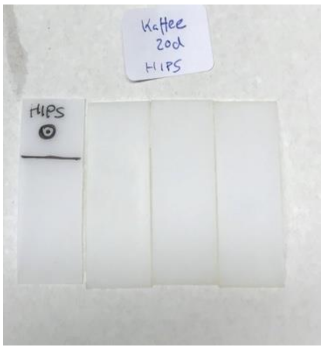	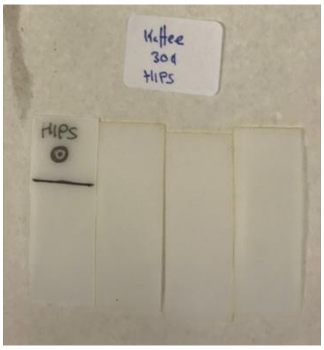

14.9 Noodles at 20 °C

10 d	20 d	30 d
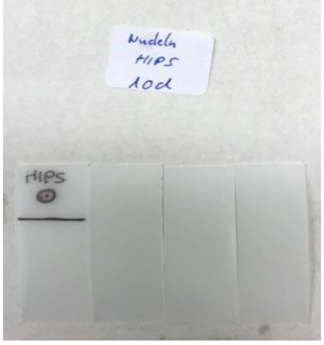	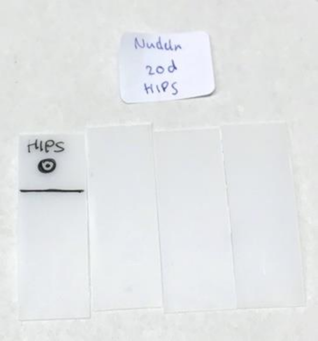	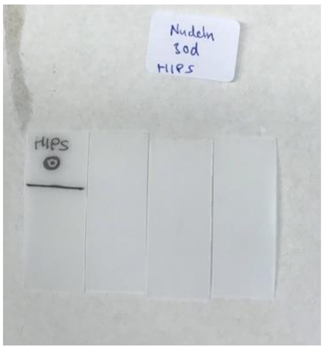

14.10 Oat flakes at 20 °C

10 d	20 d	30 d
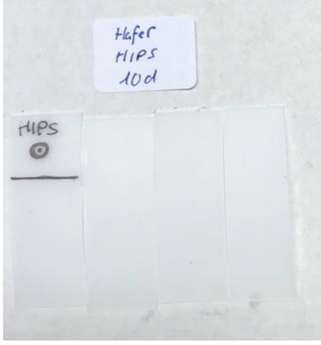	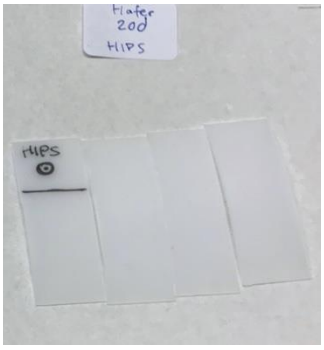	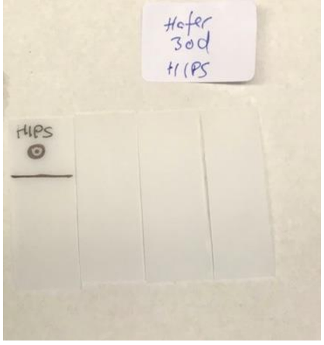

14.11 Wheat loops at 20 °C

10 d	20 d	30 d
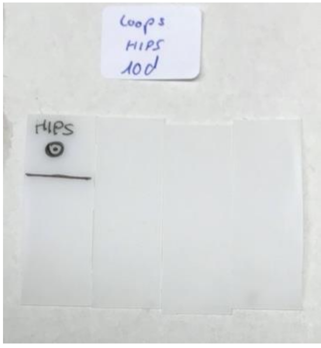	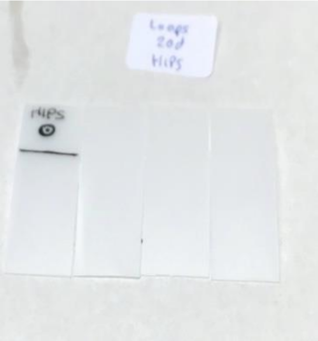	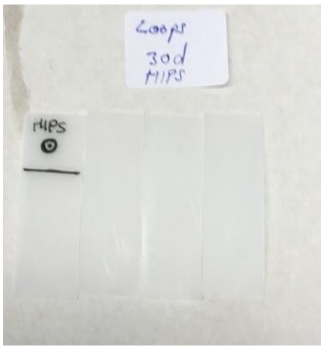

14.12 Water at 20 °C

10 d	20 d	30 d
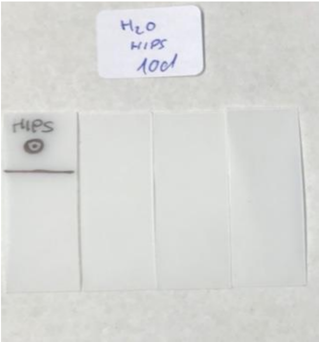	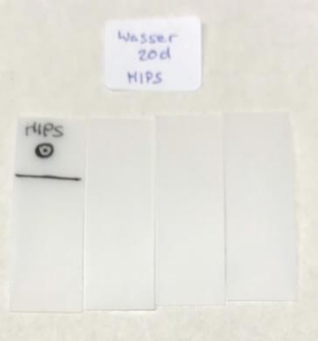	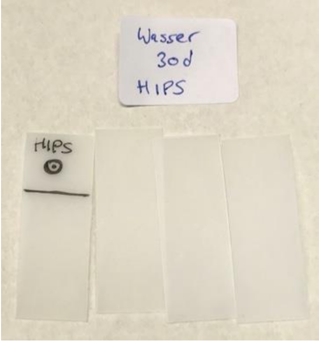

14.13 Water at 40 °C

10 min	20 min	30 min
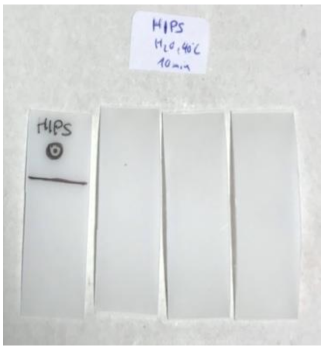	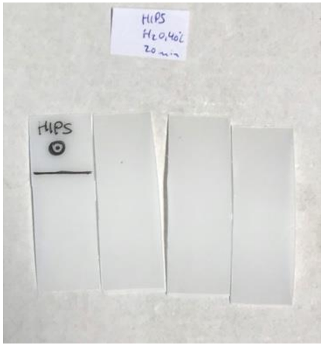	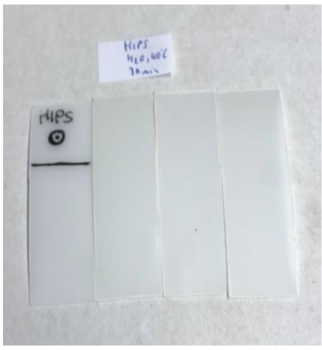

14.14 Water at 60 °C

10 min	20 min	30 min
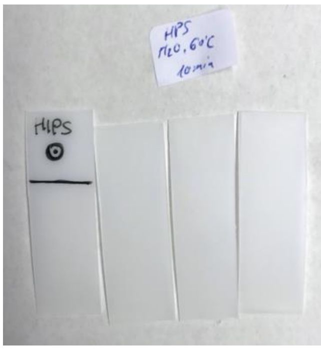	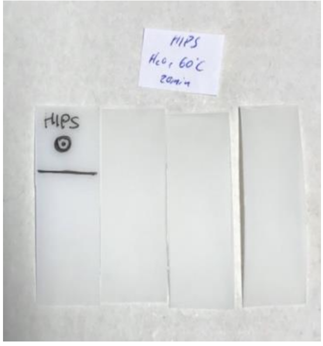	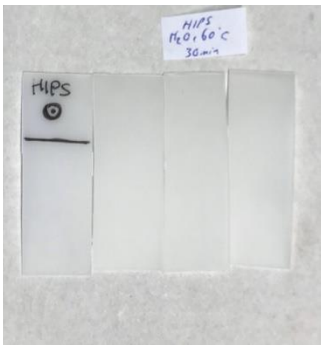

14.15 Water at 80 °C

10 min	20 min	30 min
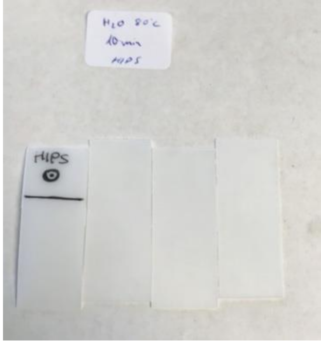	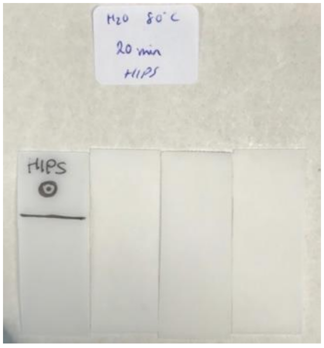	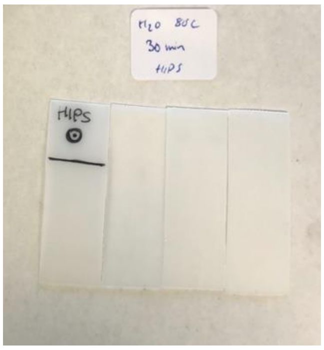

14.16 Water at 100 °C

10 min	20 min	30 min
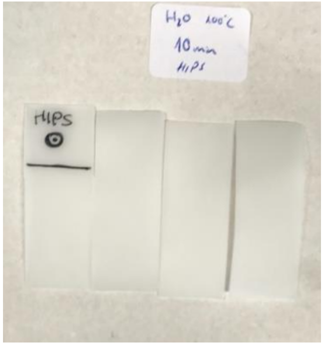	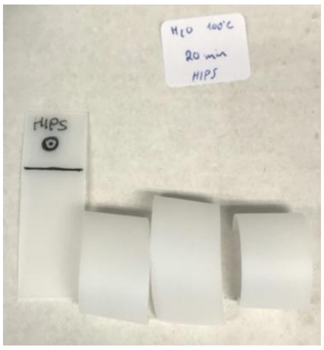	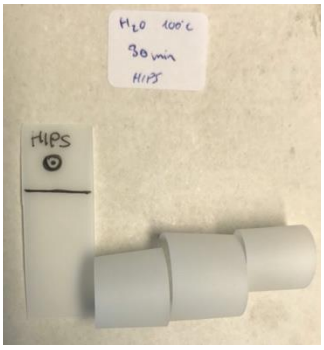

14.17 Brewed coffee at 80 °C

10 min	20 min	30 min
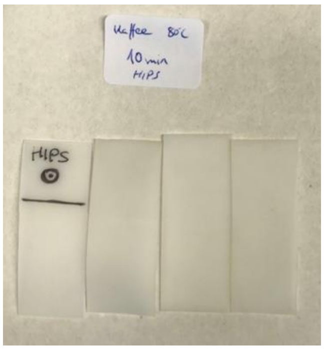	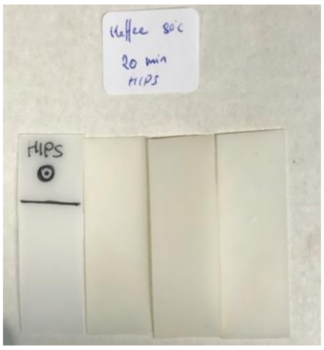	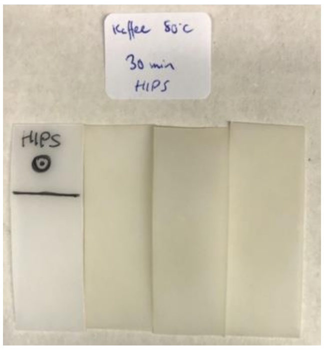

14.18 Brewed coffee at 100 °C ($T_{gradient}$)

10 min	20 min	30 min
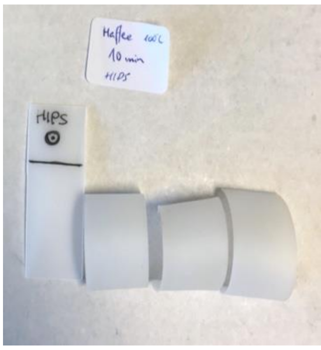	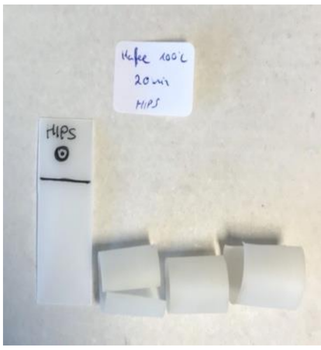	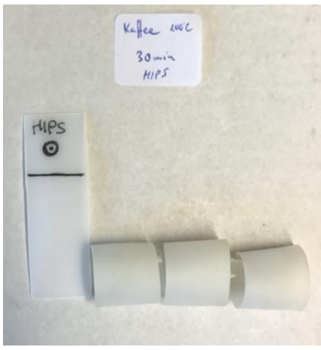

14.19 Brewed coffee at 100 °C ($T_{constant}$)

10 min	20 min	30 min
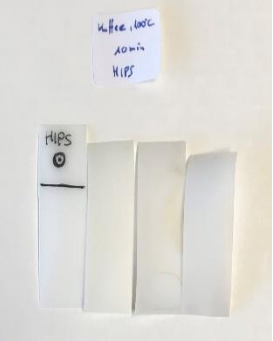	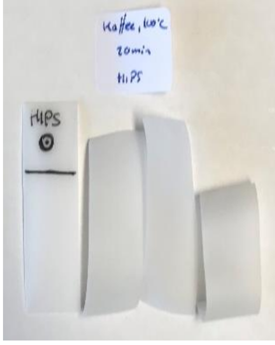	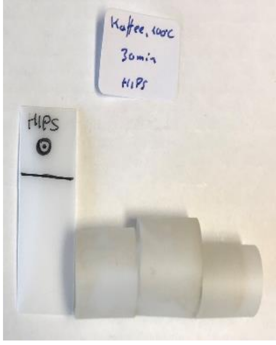
